# A multiscale cross attention framework for predicting the electromagnetic coupling characteristics of low dimensional In-Pb heterostructures fabricated by hot pressing

**DOI:** 10.3389/fchem.2026.1974299

**Published:** 2026-10-16

**Authors:** YingHao Xi, Lin Wang

**Affiliations:** School of Materials Science and Engineering, Shanghai University, Shanghai, China

**Keywords:** electromagnetic coupling tensor, hot pressing, low dimensional In-Pb heterostructures, multiscale cross attention, Onsager reciprocity constraint, process window inverse design

## Abstract

Low dimensional In-Pb heterostructures fabricated by hot pressing offer distinctive potential in superconducting quantum devices, spintronics, and flexible electromagnetic functional materials. Their electromagnetic coupling response is governed by atomic layer spin-orbit coupling, the In-Pb interfacial proximity effect, mesoscale texture, and macroscopic processing conditions acting together, and a predictive framework combining physical consistency with inverse process design has been lacking. This study proposes the Multiscale Cross Attention Electromagnetic Coupling Network, MSCA-EMNet. A hierarchical encoder embeds the four scales into independent, nonoverlapping channels, and a bidirectional cross scale attention module, BS-CrossAttn, places the Query at scale k and the Key and Value at scale k±1 , running upward aggregation and downward constraint feedback in parallel. A FiLM mechanism maps hot pressing parameters and the applied magnetic field to channel level affine parameters supporting inverse determination of feasible windows, and a scale availability mask lets records of unequal scale coverage train together through a learnable absent process embedding. Onsager reciprocity and magnetoelectric reciprocity between the direct and converse responses keep the nine component tensor physically consistent, the tensor being defined at a specified magnetic field and including three components of the interfacial magnetoelectric susceptibility. On 1,200 samples from DFT calculations and hot pressing experiments, MSCA-EMNet reaches an overall normalized mean absolute error of 0.041 and a coefficient of determination of 0.948, improvements of 43% and 7.2% over the strongest baseline. The extrapolation degradation rate is 48.8%, the proportion of samples violating the Onsager relation falls from the baseline range of 12%–41% to 1.7%, and cross system transfer reaches 0.86 to 0.89. An inverse design case maps a target tensor to a window of 168°C–174 °C, 62–70 MPa, 25–35 min and 8–12 μm, and six confirmatory experiments reproduce the target within 7.6% on every component.

## 1 Introduction

Low dimensional In-Pb heterostructures combine the strong spin-orbit coupling of Pb with the conductive channels of In, and therefore offer value in superconducting quantum devices, spintronics, flexible electromagnetic shielding, and high density packaging interconnects. Hot pressing consolidates them near the melting point of In, approximately 156 °C, forming layered, particle/matrix and alternating lamellar morphologies, and is a feasible route to scalable fabrication. Device performance depends on the coupled response of electrical conductivity, magnetic susceptibility, Hall response, and the interfacial magnetoelectric susceptibility that arises from local inversion symmetry breaking at the In-Pb boundary. These quantities depend on atomic layer SOC, the In-Pb interface, mesoscale texture, and macroscopic processing parameters at once, so reliable prediction of the electromagnetic coupling response determines whether the materials can move from laboratory research to engineering application. MSCA-EMNet is developed around this bottleneck, and its overall framework is shown in Figure 1.

**FIGURE 1:**
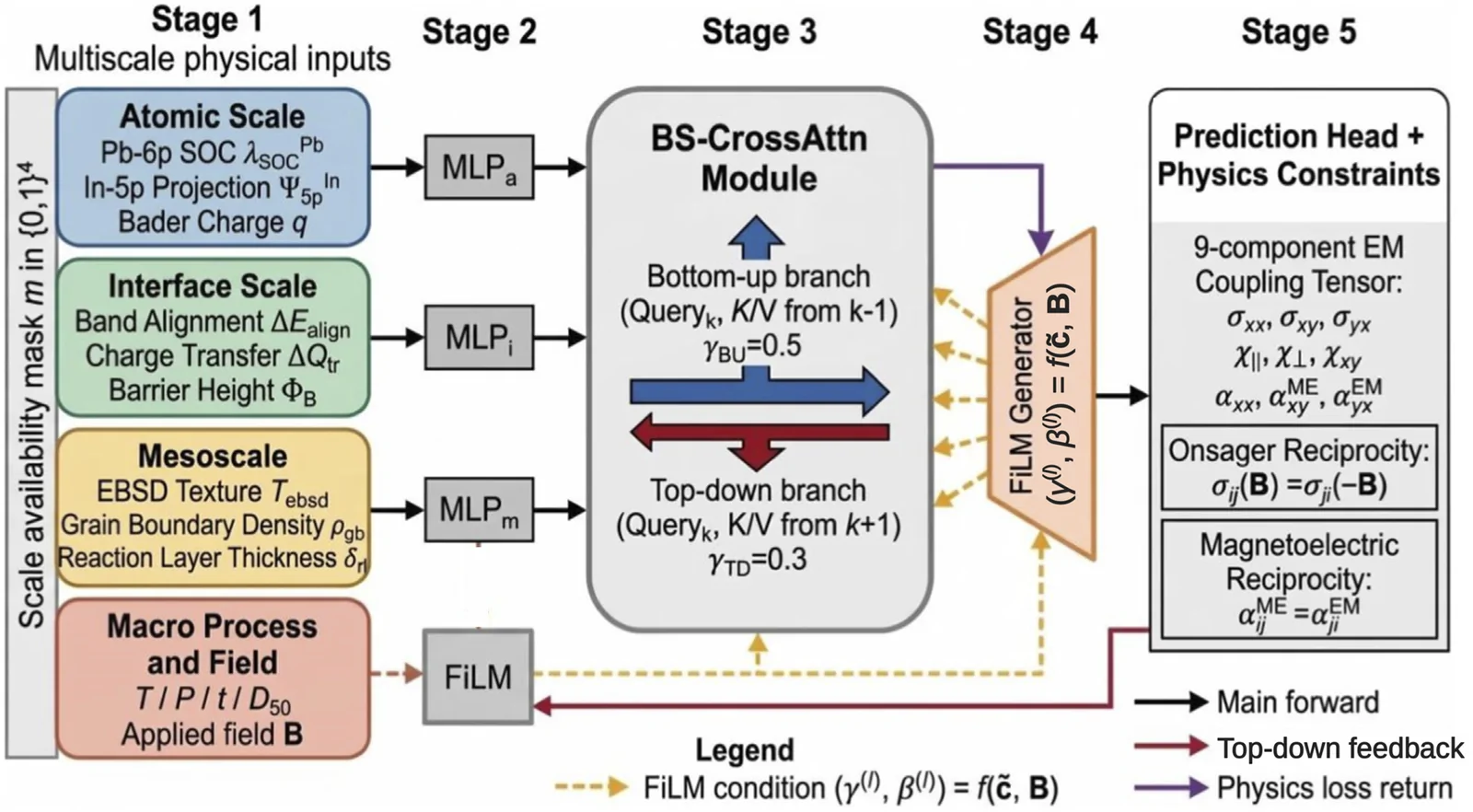
Overall research framework of MSCA-EMNet.

Research on SOC in heavy metals such as Pb has focused on ultrathin epitaxial films and van der Waals heterostructures, revealing spin momentum locking and Rashba splitting (Yoshizawa et al., 2021), and gate controlled proximity effects show that the interfacial spin structure governs the macroscopic response (Bobkov et al., 2024; Ianovskaia et al., 2024). Systematic modeling remains unavailable for hot pressed polycrystalline In-Pb containing reaction layers. Studies of liquid Ga based alloys and their In-Sn derivatives provide a reference for In in metal/metal bonding (Babatain et al., 2024; Hillaire et al., 2024), and two dimensional heterostructure synthesis is well developed (Zhang et al., 2023), yet no quantitative electromagnetic mapping exists for hot pressing induced textures and reaction layers. Machine learning Hamiltonian frameworks reduce the cost of relativistic SOC calculations (Zhong et al., 2024) but remain bound to unit cell scales and cannot cover mesoscale texture. Graph networks and attention mechanisms predict scalar properties well (Choudhary and DeCost, 2021; Wang et al., 2021) and equivariant tensor networks deliver piezoelectric and dielectric components (Batzner et al., 2022; Dong et al., 2025), yet they predict single properties, neglect processing conditions, and cannot output a complete coupling tensor. Multiscale attention transferred from computer vision degenerates into multiresolution convolution without physical correspondence (Massa et al., 2024), and feature concatenation dilutes the processing signal. Onsager reciprocity is experimentally established for transport tensors (Guo et al., 2023) but is rarely embedded as a soft constraint in deep learning losses (Madani et al., 2025). Together these gaps have prevented a framework offering physical consistency, tensor completeness and process inversion at once.

To address this gap, this study proposes MSCA-EMNet, whose architecture is shown in Figure 1. A hierarchical encoder embeds the atomic scale Pb-6p SOC strength, In-5p orbital projection and Bader charge, the interfacial band alignment, charge transfer and barrier height, the mesoscale EBSD texture index, grain boundary density and reaction layer thickness, and the macroscopic parameters T , P , t and $D_{50}$ into four nonoverlapping channels. Because the two data branches do not carry identical scale coverage, every record also carries a scale availability mask, and the processing channel is driven by a learnable absent process embedding whenever hot pressing parameters do not exist, so such a record contributes no gradient to the processing dependent parameters. BS-CrossAttn places the Query at scale k and the Key and Value at scale k±1 , running an upward aggregation branch and a downward process constraint feedback branch in parallel so that macroscopic parameters revise atomic layer embeddings through the composition of adjacent scale attention maps. The prediction head outputs nine tensor components, defined at a specified out of plane field B and comprising the longitudinal and transverse conductivities, three susceptibility components, and three components of the interfacial magnetoelectric susceptibility in ps·m^‒1^. Onsager reciprocity, $\sigma_{ij}\left(B\right)=\sigma_{ji}\left(-B\right)$ , and magnetoelectric reciprocity, $\alpha_{ij}^{ME}=\alpha_{ji}^{EM}$ , enter the loss as soft constraints evaluated on destandardized physical values, so the constraint weights carry a unit independent meaning. FiLM converts the processing parameters into channel level affine modulation, establishing a differentiable path from processing conditions to performance that supports inverse determination of feasible windows. A worked inverse design case is reported in Section 4.5. These designs give MSCA-EMNet physical interpretability, tensor completeness, and a closed process loop.

## 2 Related work

### 2.1 Transferability of strong spin-orbit coupling descriptions to hot pressed polycrystals

Research on spin-orbit coupling in Pb and heavy metal systems has relied on clean, epitaxial or van der Waals heterostructures with well defined interfaces. First principles extraction of SOC parameters assumes perfect symmetry (Zollner et al., 2025), proximity effect interpretations depend on single crystal interface quality (Flokstra et al., 2023), and van der Waals magnetic heterostructures are attributed to subatomic interface control (Ma et al., 2025). The resulting Rashba framework (Bihlmayer et al., 2022) does not transfer to hot pressed systems containing reaction layers, randomly oriented particles and intermetallic compounds, and In based layered ferroelectrics (Sui et al., 2023) likewise rely on ordered geometries. In hot pressed In-Pb the SOC signal combines grain interiors, proximity effects and reaction layer electronic structure, which couple nonlinearly under field and temperature, and existing theories offer neither a quantitative decoupling framework nor a transferable bridge between characterization and modeling.

### 2.2 Absence of a traceable mapping from processing history to mesoscale structure

Hot pressing couples thermal, mechanical, electrical and chemical fields. Multiphysics simulations are limited to particle scale density and grain growth (Semenov et al., 2023), phase field models reproduce vacancy diffusion and boundary migration but not intermetallic nucleation at In-Pb interfaces (Yi et al., 2023), and coupled pressure diffusion models require a full new solution for each parameter combination (Zhao et al., 2022). Universal machine learning potentials (Chen and Ong, 2022; Deng et al., 2023) and equivariant local representations (Musaelian et al., 2023) are trained on static structures without hot pressing time or loading paths. A traceable mapping from processing parameters to mesoscale texture therefore remains unavailable.

### 2.3 Joint regression of cross response tensors and the origin of the magnetoelectric channel

The response of real In-Pb systems couples susceptibility, conductivity, anomalous Hall response and magnetoelectric coefficients, as in multiferroics and heterostructures (Geng et al., 2022; Wang et al., 2023; Xu and Yang, 2024). The magnetoelectric channel of a hot pressed polycrystal needs care, because a spontaneous linear magnetoelectric effect demands simultaneous breaking of spatial inversion and time reversal symmetry, which a nonmagnetic centrosymmetric polycrystal does not satisfy in the bulk. The relevant response is interfacial and field induced: inversion symmetry is broken locally at the In/Pb boundary and within the reaction layer, the strong Pb-6p SOC converts this asymmetry into Rashba type spin momentum locking, and first principles calculations show that the resulting current induced spin polarization at metallic interfaces constitutes a magnetoelectric susceptibility of measurable magnitude even in nonmagnetic metals (Droghetti and Tokatly, 2023). Whether it survives polycrystalline averaging is controlled by texture rather than chemistry, since contributions of differently oriented interfaces cancel only for a fully random distribution, and measurements on polycrystalline heavy metal films confirm that the conversion efficiency varies with texture (Kim et al., 2023). Hot pressing controls exactly this quantity. Existing methods treat electrical and magnetic properties as independent scalars or give equivariant predictions for single channels (Mao et al., 2024), so cross response tensors are absent from training sets, outputs cannot satisfy Onsager or magnetoelectric reciprocity, and correlated errors are amplified in device design.

### 2.4 Degeneration of multiscale architectures into multiresolution feature extraction

The architecture of materials models adds a further difficulty. Multi-head self attention and graph attention have replaced message passing in crystal property prediction (Schmidt et al., 2021; Xu et al., 2024), but their multiscale character arises from receptive fields or hop counts rather than a physical hierarchy. Transformers for metal-organic frameworks (Kang et al., 2023) and GNN reviews (Reiser et al., 2022) fuse modalities by concatenation or gating, flattening processing conditions and microscopic features into one space, and large scale training shows that capacity alone does not decouple physical hierarchies (Merchant et al., 2023). Information across scales lacks directionality, process constraints cannot feed back to atomic embeddings, and attention weights are hard to associate with physical mechanisms. These limitations motivate BS-CrossAttn and the FiLM modulation module.

## 3 Design of the MSCA-EMNet prediction framework

### 3.1 Problem formulation and overall architecture

MSCA-EMNet formulates the prediction as a multi-output tensor regression over an input space spanning several physical scales. Given the atomic input $X_{a}\in R^{d_{a}}$ , the interfacial input $X_{i}\in R^{d_{i}}$ , the mesoscale input $X_{m}\in R^{d_{m}}$ and the hot pressing vector $c=\left(T,P,t,D_{50}\right)$ , together with the scale availability mask m and the out of plane field B at which the response is defined, the model learns the differentiable mapping given in Equation 1:

$$\hat{Y}=f_{\theta }\left(X_{a},X_{i},X_{m},m;c,B\right)$$ (1)

where $Y=\left\{\sigma_{xx},\sigma_{xy},\sigma_{yx},\chi_{∥},\chi_{⊥},\chi_{xy},\alpha_{xx},\alpha_{xy}^{ME},\alpha_{yx}^{EM}\right\}\in R^{9}$ covers electrical conductivity, magnetic susceptibility and interfacial magnetoelectric susceptibility; θ denotes the learnable parameters; and T , P , t , $D_{50}$ are the hot pressing temperature, pressure, holding time and median powder particle size. The mask $m=\left(m_{a},m_{i},m_{m},m_{c}\right)\in {\left\{0,1\right\}}^{4}$ records which of the four blocks is genuinely available for each sample, and a masked block is replaced by a learnable absent scale embedding rather than by an imputed value, as given in Equation 2:

$${\tilde{X}}_{s}=m_{s}X_{s}+\left(1-m_{s}\right)u_{s},s\in \left\{a,i,m,c\right\}$$ (2)

where $u_{s}$ is a trainable vector of the same dimension as the block. The gradient with respect to a masked block vanishes, so a record without hot pressing parameters trains the encoders, BS-CrossAttn and the prediction head but not the process dependent modulation. The field is treated separately from c and is available for every record. The composition of the mask across the two data sources is documented in Section 4.1.

Equation 1 replaces the degeneration of multiscale into multiresolution representation criticized in Section 2.4 by an explicit separation of physical scales. The parameter c does not enter the input vector but acts on each layer through the FiLM channel, preventing dilution by high dimensional atomic features, and the same $f_{\theta }$ outputs all nine components rather than nine regressors. Because the response is field conditioned, one configuration evaluated at +B and at −B is a single paired record. Onsager and magnetoelectric reciprocity are embedded in the loss and the pipeline stays differentiable, so processing parameters can be inverted through backward gradients. Figure 2 marks the dimensions and targets of every transformation: the yellow path is the FiLM channel for processing conditions and the applied field, the blue path the backbone flow, and the red line the downward constraint loop.

**FIGURE 2:**
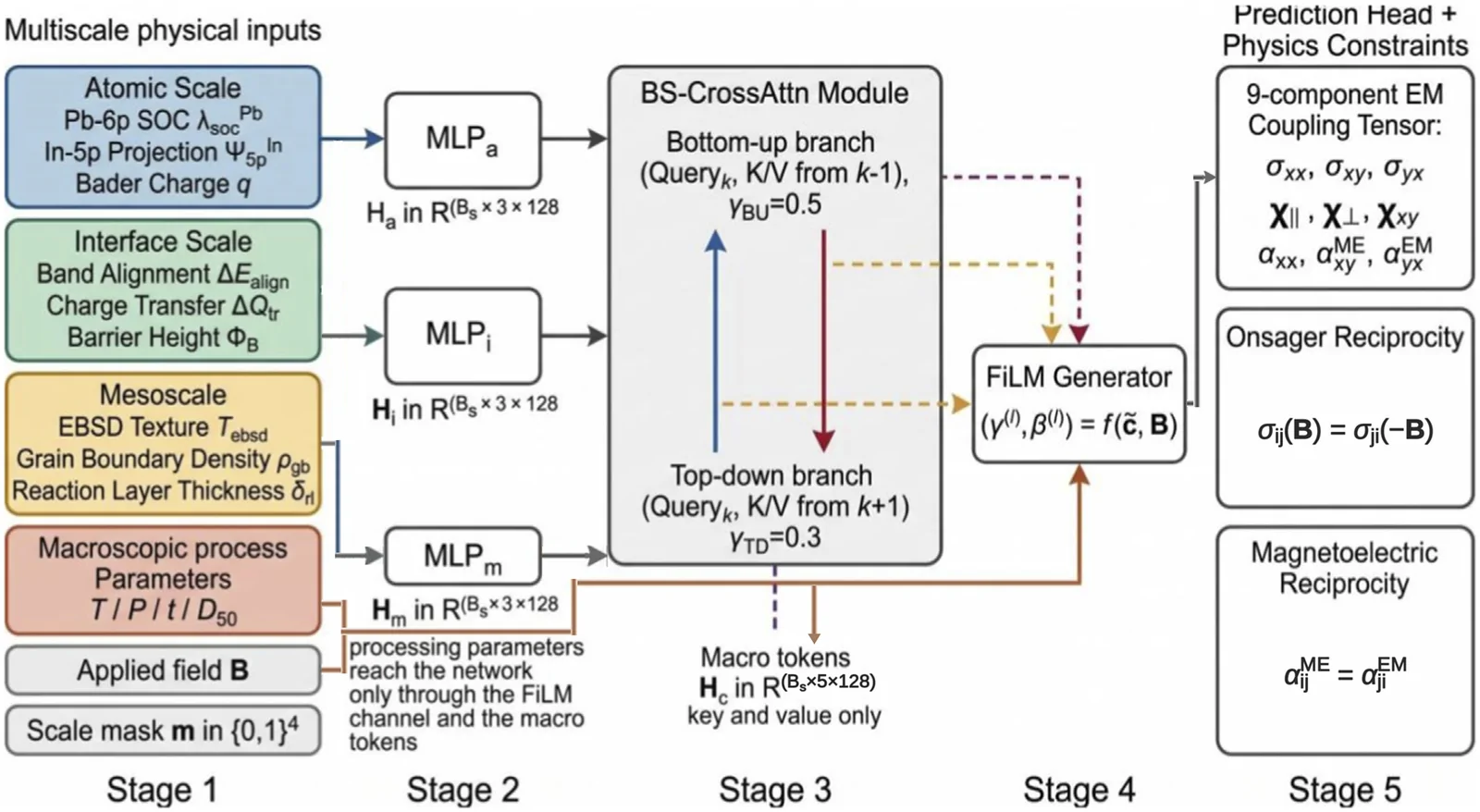
Detailed data flow and module interactions in MSCA-EMNet.

### 3.2 Hierarchical physical scale feature encoder

The four categories of physical inputs do not overlap, and their encoding follows Equations 3–8. The atomic encoder takes the Pb-6p SOC strength $\lambda_{SOC}^{Pb}$ , the In-5p orbital projection $\Psi_{5p}^{In}$ and the Bader charge q , all extracted from DFT plus SOC calculations of the powders before hot pressing:

$$h_{a}={MLP}_{a}\left(\left[\lambda_{SOC}^{Pb},\Psi_{5p}^{In},q\right]\right)\in R^{3\times d_{h}}$$ (3)

where $MLP_{a}$ is a two layer fully connected network applied to each of the three quantities separately and $d_{h}$ is the unified hidden dimension, set to 128 throughout this work. The interfacial input comprises the band alignment $\Delta E_{align}$ , charge transfer $\Delta Q_{tr}$ and barrier height $\Phi_{B}$ at the In-Pb interface, which reflect the modification of the local electronic structure by the proximity effect:

$$h_{i}={MLP}_{i}\left(\left[\Delta E_{align},\Delta Q_{tr},\Phi_{B}\right]\right)\in R^{3\times d_{h}}$$ (4)

The mesoscale input comprises the EBSD texture index $T_{ebsd}$ , grain boundary density $\rho_{gb}$ and reaction layer thickness $\delta_{rl}$ , the texture index being the excess of the orientation distribution over its random value, so that it vanishes for a random texture and rises as a preferred orientation develops:

$$h_{m}=MLP_{m}\left(\left[T_{ebsd},\rho_{gb},\delta_{rl}\right]\right)\in R^{d_{h}}$$ (5)

The macroscopic scale consists of the hot pressing parameters, which are routed to the FiLM channel and do not enter the main encoder path:

$$c=\left(T,P,t,D_{50}\right)\in R^{4}$$ (6)

Each scale block is tokenized at the level of individual physical quantities rather than compressed into a single vector, so that attention operates over a genuine token axis. The three quantities of scale k are embedded separately and stacked, which gives

$$H_{k}=\left[h_{k}^{\left(1\right)},h_{k}^{\left(2\right)},h_{k}^{\left(3\right)}\right]\in R^{B_{s}\times n_{k}\times d_{h}}$$ (7)

where $B_{s}$ is the batch size and $n_{k}$ = 3. The macroscopic scale is embedded in the same token form and serves exclusively as Key and Value in the downward branch, preserving the rule that processing conditions never enter the upward main path:

$$H_{c}=MLP_{c}\left(\left[\tilde{c},B\right]\right)\in R^{B_{s}\times n_{c}\times d_{h}}$$ (8)

The number of macroscopic tokens is $n_{c}$ = 5, corresponding to T , P , t , $D_{50}$ and B , and $\tilde{c}$ is the masked processing vector of Equation 2. Table 1 lists the input variables, symbols, units and sources, so the correspondence among physical levels can be audited, and Figure 3 shows the encoder, in which the three branches are mapped by separate MLPs into hidden spaces of dimension $d_{h}$ .

**TABLE 1:** Input variables across four physical scales in MSCA-EMNet.

Scale	Physical quantity	Symbol	Unit	Data source
Atomic level	Pb-6p SOC strength	$\lambda_{SOC}^{Pb}$	eV	DFT plus SOC calculations
Atomic level	In-5p orbital projection	$\Psi_{5p}^{In}$	Dimensionless	DFT orbital projection
Atomic level	Bader charge	q	e	Bader charge analysis
Interfacial level	Band alignment	$\Delta E_{align}$	eV	DFT interface band calculations
Interfacial level	Charge transfer	$\Delta Q_{tr}$	e·nm^−2^	Differential charge density integration
Interfacial level	Barrier height	$\Phi_{B}$	eV	Interface barrier energy estimation
Mesoscale level	Texture index	$T_{ebsd}$	Dimensionless	EBSD orientation distribution, excess over the random value
Mesoscale level	Grain boundary density	$\rho_{gb}$	μm^‒1^	EBSD image analysis
Mesoscale level	Reaction layer thickness	$\delta_{rl}$	nm	STEM/HAADF characterization
Macroscopic level	Hot pressing temperature	T	°C	Experimental setting
Macroscopic level	Hot pressing pressure	P	MPa	Experimental setting
Macroscopic level	Holding time	t	min	Experimental setting
Macroscopic level	Powder particle size	$D_{50}$	μm	Laser particle size analyzer
Measurement condition	Applied magnetic field	B	T	PPMS transport and magnetization setting; reversed spin quantization axis in DFT plus SOC
Availability	Scale availability mask	m	Dimensionless	Dataset construction record, Section 4.1

**FIGURE 3:**
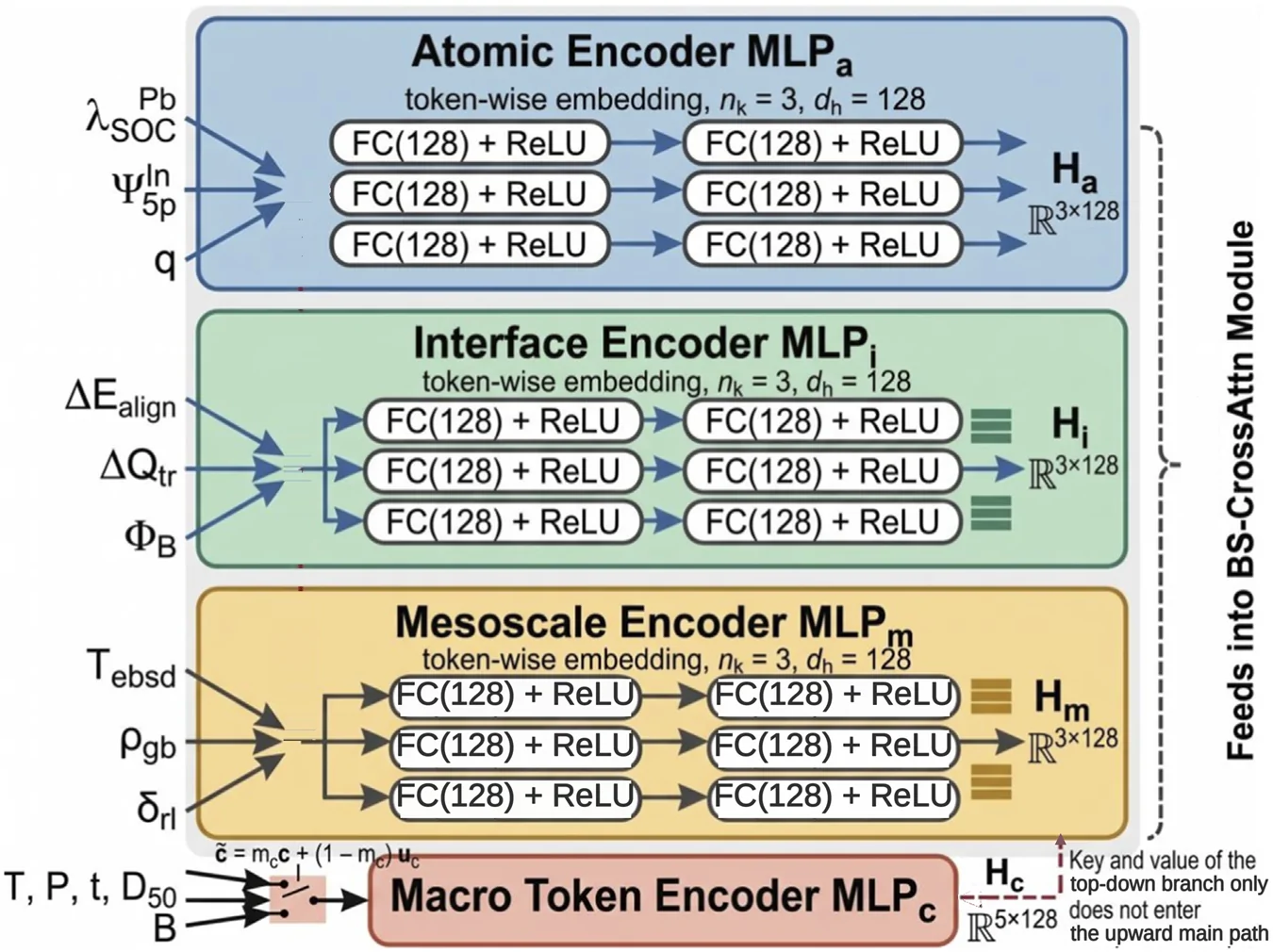
Architecture of the hierarchical physical scale feature encoder.

### 3.3 Bidirectional cross scale attention module, BS-CrossAttn

BS–CrossAttn, formulated in Equations 9–14, is the central architectural innovation. The Query always originates from scale k and the Key and Value from scale k±1 , which enforces genuine cross scale flow, and an upward aggregation branch and a downward feedback branch run in parallel, lifting the conventional restriction that information may flow only from fine to coarse scales. The dot product cross attention is

$$\text{Attention}\left(Q,K,V\right)=\text{softmax}\left(\frac{QKT}{\sqrt{d_{k}}}\right)V$$ (9)

where Q , K and V are the query, key and value matrices. Attention uses $n_{head}$ = 4 heads of dimension $d_{k}$ = 32, so that $n_{head}d_{k}=d_{h}$ . In the upward branch the Query at scale k aggregates the Key and Value from the finer scale k−1 , propagating atomic electronic structure information to the interfacial and mesoscale levels:

$$Q_{k}^{BU}=H_{k}W_{Q}^{BU},K_{k-1}^{BU}=H_{k-1}W_{K}^{BU},V_{k-1}^{BU}=H_{k-1}W_{V}^{BU}$$ (10)

$$H_{k}^{BU}=\text{Attention}\left(Q_{k}^{BU},K_{k-1}^{BU},V_{k-1}^{BU}\right)$$ (11)

where $W_{Q}^{BU}$ , $W_{K}^{BU}$ and $W_{V}^{BU}$ are learnable projections and $H_{k}$ is the token tensor from the encoder at scale k . The downward branch lets scale k absorb the Key and Value from scale k+1 , feeding macroscopic conditions and mesoscale texture back to the finer embeddings:

$$Q_{k}^{TD}=H_{k}W_{Q}^{TD},K_{k+1}^{TD}=H_{k+1}W_{K}^{TD},V_{k+1}^{TD}=H_{k+1}W_{V}^{TD}$$ (12)

$$H_{k}^{TD}=\text{Attention}\left(Q_{k}^{TD},K_{k+1}^{TD},V_{k+1}^{TD}\right)$$ (13)

At the mesoscale level the downward Key and Value come from the macroscopic token tensor $H_{c}$ of Equation 8, and at the atomic level the upward branch is inactive. The query has shape ( $B_{s}$ , $n_{head}$ , $n_{k}$ , $d_{k}$ ) and the key and value ( $B_{s}$ , $n_{head}$ , $n_{k\pm 1}$ , $d_{k}$ ), so the score matrix $Q_{k}{K^{T}}_{k\pm 1}$ has shape $B_{s}$ , $n_{head}$ , $n_{k}$ , $n_{k\pm 1}$ ). The softmax acts on the last axis, the key token axis, and the weighted sum returns ( $B_{s}$ , $n_{head}$ , $n_{k}$ , $d_{k}$ ) merged back to ( $B_{s}$ , $n_{k}$ , $d_{h}$ ). No operation is performed along the batch axis at any step, so samples never exchange information during training or inference, and the reported maps are per sample quantities of size $n_{k}\times n_{k\pm 1}$ .

The two branch outputs are fused with the original representation through learnable gating scalars:

$$H_{k}^{new}=H_{k}+\gamma_{BU}H_{k}^{BU}+\gamma_{TD}H_{k}^{TD}$$ (14)

where $\gamma_{BU}$ and $\gamma_{TD}$ are initialized to 0.5 and 0.3 to prevent gradient imbalance in early training. Because Key and Value are restricted to adjacent scales, a coupling between non adjacent levels is the composition of the intermediate maps rather than a single weight, in both directions. Denoting by $A_{k\leftarrow k-1}^{BU}$ and $A_{k\leftarrow k+1}^{TD}$ the per sample attention matrices at scale k ,

$${\bar{A}}_{m\leftarrow a}^{BU}=A_{m\leftarrow i}^{BU}A_{i\leftarrow a}^{BU}\in R^{n_{m}\times n_{a}},{\bar{A}}_{a\leftarrow c}^{TD}=A_{a\leftarrow i}^{TD}A_{i\leftarrow m}^{TD}A_{m\leftarrow c}^{TD}\in R^{n_{a}\times n_{c}}$$ (15)

Both composed matrices are row stochastic and supply the atomic to mesoscale and process to atomic weights of Figure 4. Figure 5 shows the complete data flow. The upward branch transfers electronic structure to macroscopic response, while the downward branch corrects from processing conditions to microstructure and then to electronic structure along the composed path of Equation 15 rather than waiting for FiLM. The stack depth is $L_{bs}$ = 2 in all experiments, quantified by the depth ablation in Section 4.3. Gates are shared across layers whereas projections are not, extending depth capacity at linear parameter cost. A reference implementation is given in Supplementary Section S14.

**FIGURE 4:**
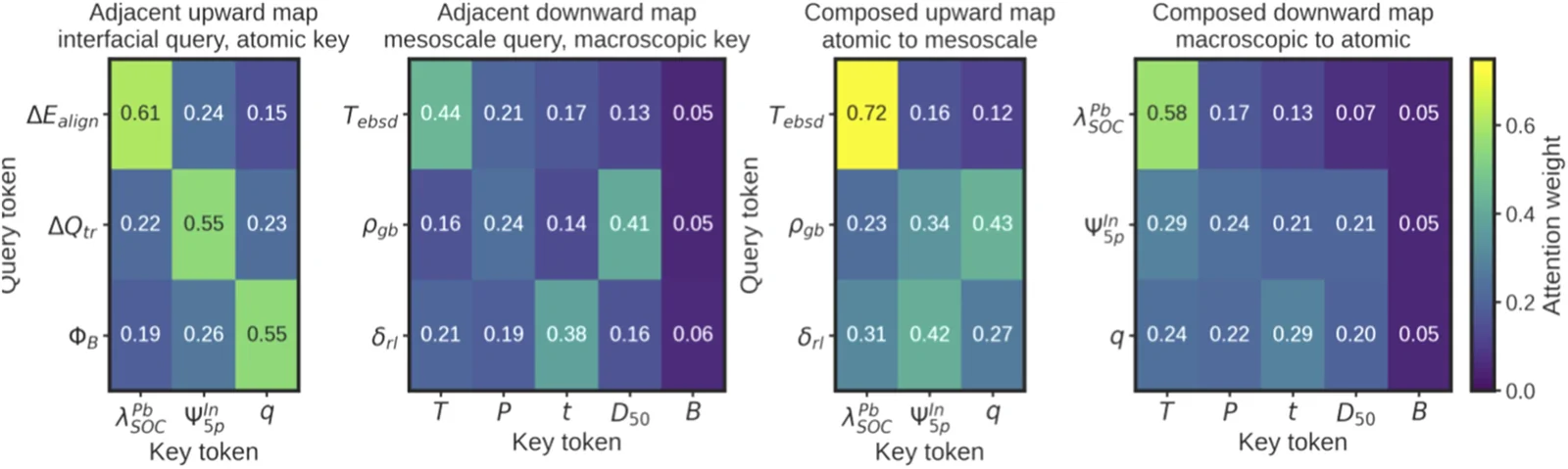
Token level cross scale attention maps of BS-CrossAttn for a representative sample at layer 2, comprising the adjacent upward map, the adjacent downward map, the composed atomic to mesoscale map and the composed macroscopic to atomic map.

**FIGURE 5:**
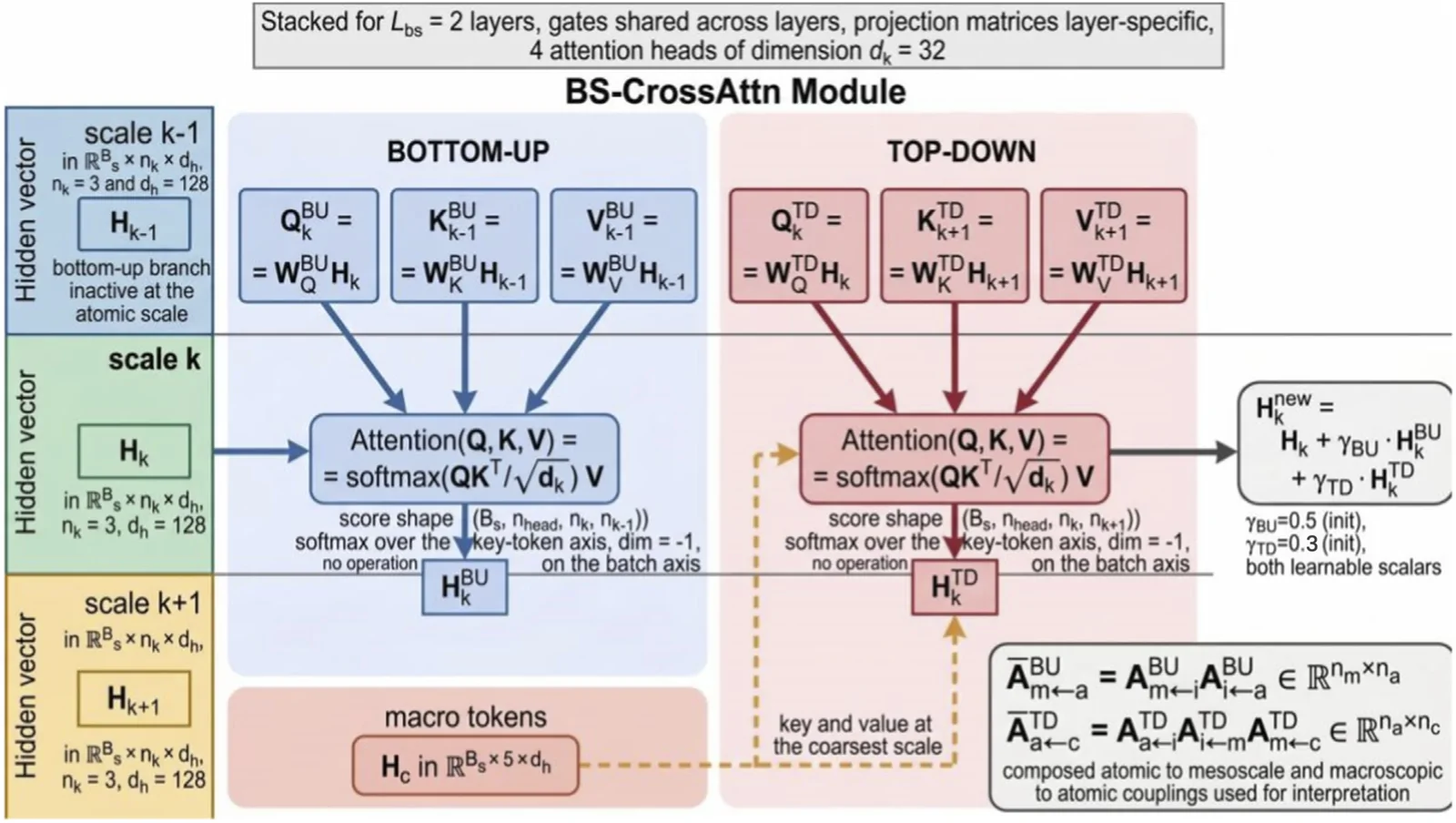
Architecture of the BS-CrossAttn bidirectional cross scale attention module.

### 3.4 FiLM modulation mechanism for processing parameters

Processing parameters are injected through FiLM, as formulated in Equations 16, 17, replacing parameter concatenation. Two compact MLPs map the conditioning vector $\xi =\left(\tilde{c},B\right)\in R^{5}$ , formed by the masked processing vector of Equation 2 and the applied field, to the channel level affine parameters $\left(\gamma^{\left(l\right)},\beta^{\left(l\right)}\right)$ of each layer:

$$\gamma^{\left(l\right)}=MLP_{\gamma }^{\left(l\right)}\left(\xi \right),\beta^{\left(l\right)}=MLP_{\beta }^{\left(l\right)}\left(\xi \right)$$ (16)

where l is the layer index and $\gamma^{\left(l\right)}$ and $\beta^{\left(l\right)}$ have the channel dimension of layer l . The intermediate feature $z^{\left(l\right)}$ then becomes

$${\tilde{z}}^{\left(l\right)}=\gamma^{\left(l\right)}⊙z^{\left(l\right)}+\beta^{\left(l\right)}$$ (17)

where ⊙ is channelwise multiplication. Channels sensitive to processing conditions are amplified and insensitive channels suppressed, so the processing signal enters again at every layer instead of being a small diluted segment of the input vector. Records without a hot pressing history are handled by the same generator without imputation. When $m_{c}$ equals 0 the four processing components of ξ are replaced by the learnable absent process embedding $u_{c}$ , the field component stays active, and the gradient with respect to T , P , t and $D_{50}$ vanishes. Because the final layer producing γ is initialized with zero weights and unit bias while the final layer producing β is initialized to zero, the modulation starts as an identity mapping and departs from it only through the field channel and through $u_{c}$ . First principles records therefore train the encoders, the cross scale module and the prediction head, and the process dependent modulation is learned only from records that possess a hot pressing history. The FiLM channel is also decoupled from the backbone, which allows independent fine tuning for a new processing range, and it is differentiable for inverse design. Once the target indicator $Y^{*}$ is specified, the other components are fixed and c becomes a learnable variable, so a feasible window follows from gradient descent:

$$c^{*}={\mathrm{argmin}}_{c}\left\{{\left\|f_{\theta }\left(X_{a},X_{i},X_{m};c,B\right)-Y^{*}\right\|}_{2}^{2}+\lambda_{r}R\left(c\right)\right\}$$ (18)

where $R\left(c\right)$ is a soft regularizer for the feasible processing domain and $\lambda_{r}$ its weight. Equation 18 turns MSCA-EMNet from a forward predictor into a closed loop design tool and remedies the missing traceable mapping between processing parameters and mesoscale texture discussed in Section 2.2. The boundary settings, the value of $\lambda_{r}$ , the multi start protocol and the experimental confirmation are reported in Section 4.5. Figure 6 shows the parallel relationship between the FiLM generator and the backbone, with dashed lines for the conditional path and solid lines for the backbone flow. The implementation of the modulation layer is given in Supplementary Section S14.

**FIGURE 6:**
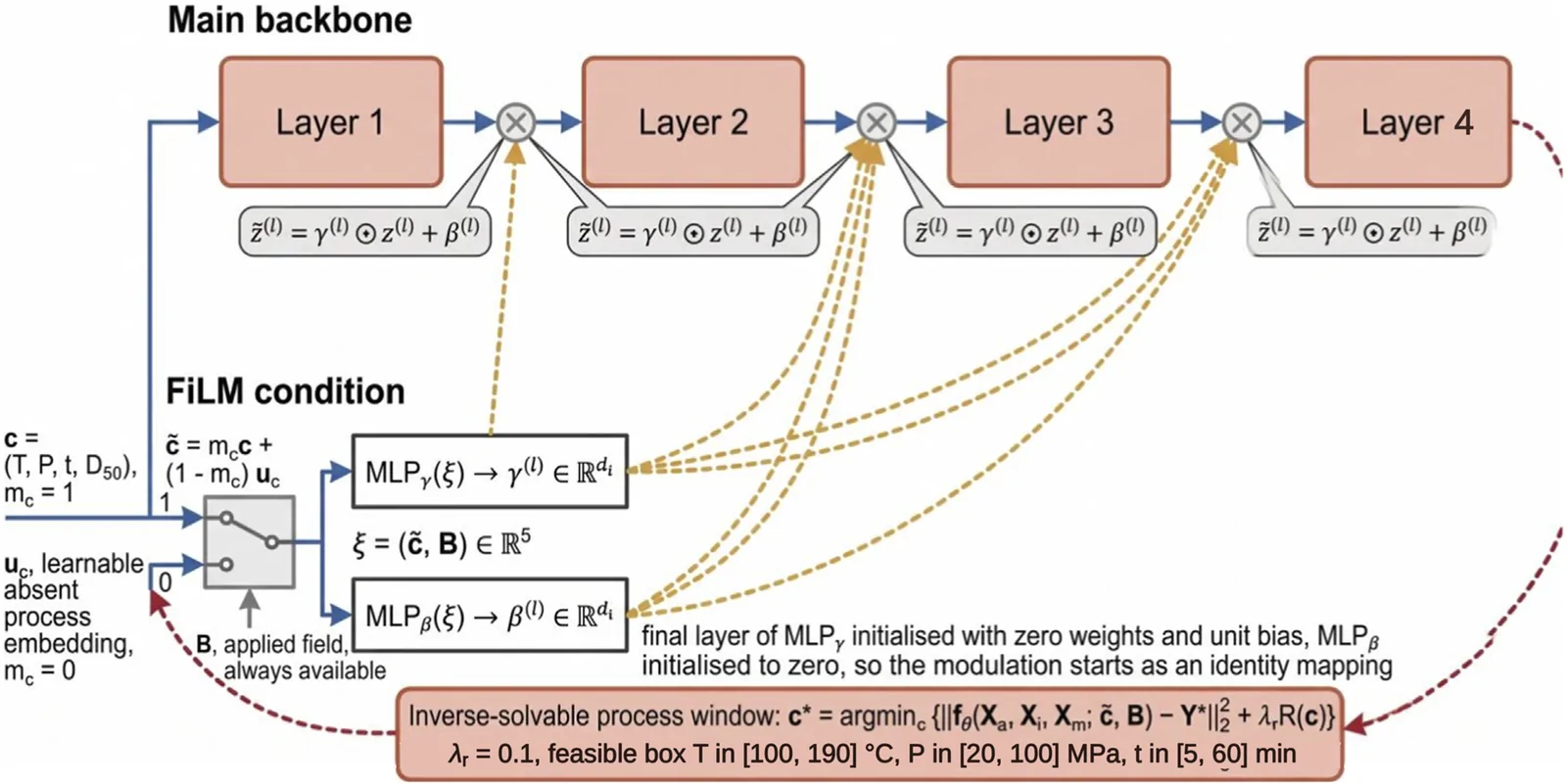
FiLM modulation mechanism for processing parameters.

### 3.5 Joint prediction head for the electromagnetic coupling tensor and physically constrained loss

The prediction head receives the fused representation $h_{fuse}$ and outputs the nine component tensor through a fully connected layer, as given in Equation 19:

$$\hat{Y}=W_{out}h_{fuse}+b_{out}\in R^{9}$$ (19)

where $W_{out}\in R^{9\times d_{h}}$ and $b_{out}\in R^{9}$ are learnable. The nine channels are $\sigma_{xx}$ , $\sigma_{xy}$ , $\sigma_{yx}$ , $\chi_{∥}$ , $\chi_{⊥}$ , $\chi_{xy}$ , $\alpha_{xx}$ , $\alpha_{xy}^{ME}$ and $\alpha_{yx}^{EM}$ , covering the conductivity tensor, the susceptibility tensor and the interfacial magnetoelectric susceptibility tensor. Joint regression of the full tensor lets downstream tasks obtain correlated errors without concatenating separate models, and it lets Onsager reciprocity and magnetoelectric reciprocity be written into the loss as differences between components.

The third response family is defined as follows. A spontaneous linear magnetoelectric effect is forbidden in a nonmagnetic polycrystal that is centrosymmetric on average, as discussed in Section 2.3, so what is predicted is the field induced interfacial magnetoelectric susceptibility. Inversion symmetry is broken locally at the In/Pb boundary and inside the reaction layer, time reversal symmetry is broken by the applied field, and the strong Pb-6p SOC turns this pair of conditions into a Rashba type response $\mu_{0}M_{i}=\alpha_{ij}^{EM}E_{j}$ and $P_{i}=\alpha_{ij}^{ME}H_{j}$ , symmetry admissible in the interface subsystem although forbidden in the bulk average (Droghetti and Tokatly, 2023). The net response is the orientation average of the interface contributions and vanishes only for a fully random distribution, so the measurable magnitude scales with the EBSD texture index rather than with composition alone (Kim et al., 2023). In a conductor the converse response is carried by the current induced magnetization, so

$$\alpha_{ij}^{EM}=\mu_{0}\chi_{ij}^{J}\sigma_{xx}$$ (20)

where $\chi_{ij}^{J}$ is the current induced magnetization coefficient, the magnetization density per unit charge current density, carrying the dimension of length. Inverting Equation 20 over the observed ranges places $\chi^{J}$ between 4.8 × 10^−14^ and 8.4 × 10^−14^ m, consistent with an interfacial Edelstein coefficient of order 10^−9^ m diluted by an interfacial volume fraction of about 2 × 10^−3^ and further reduced by incomplete orientation cancellation. Both coefficients are reported in ps·m^‒1^ and span 0.13–0.86 ps·m^‒1^, of the same order as though below prototypical single phase linear magnetoelectrics. Table 2 lists the definitions, units and ranges of all nine channels; label provenance is described in Section 4.1.

**TABLE 2:** Definitions, units, and observed ranges of the nine output components.

Channel	Symbol	Definition	Unit	Range in the dataset
1	$\sigma_{xx}$	Longitudinal conductivity at zero field	S·m^‒1^	1.8 × 10^6^ to 8.2 × 10^6^
2	$\sigma_{xy}$	Transverse conductivity at the assigned field +B	S·m^‒1^	2.0 × 10^2^ to 4.1 × 10^4^
3	$\sigma_{yx}$	Conjugate transverse conductivity at the same field	S·m^‒1^	−4.1 × 10^4^ to −2.0 × 10^2^
4	$\chi_{∥}$	Volume susceptibility along the pressing axis, measured at 0.05 T	Dimensionless	−1.62 × 10^−5^ to −0.58 × 10^−5^
5	$\chi_{⊥}$	Volume susceptibility normal to the pressing axis	Dimensionless	−1.44 × 10^−5^ to −0.62 × 10^−5^
6	$\chi_{xy}$	Off diagonal susceptibility component	Dimensionless	0.9 × 10^−7^ to 7.8 × 10^−7^
7	$\alpha_{xx}$	Diagonal interfacial magnetoelectric susceptibility	ps·m^‒1^	0.03 to 0.18
8	$\alpha_{xy}^{ME}$	Direct interfacial magnetoelectric coefficient, $\partial P_{x}/\partial H_{y}$	ps·m^‒1^	0.14 to 0.86
9	$\alpha_{yx}^{EM}$	Converse interfacial magnetoelectric susceptibility, $\partial \left(\mu_{0}M_{y}\right)/\partial E_{x}$	ps·m^‒1^	0.13 to 0.85

Two symmetry constraints are embedded in the loss. Both are evaluated on physical rather than standardized values, because a residual formed from standardized outputs would mix components of different magnitude and make the weights depend on the scaling of the training set. The prediction of channel k is mapped back to physical units and divided by the training standard deviation $s_{k}$ , giving a dimensionless residual operator, as defined in Equation 21:

$$r_{k}\left(\cdot \right)=\frac{D_{k}\left(\cdot \right)}{s_{k}},D_{k}\left(\hat{Y}\right)=s_{k}{\hat{Y}}_{k}+\mu_{k}$$ (21)

where $\mu_{k}$ and $s_{k}$ are the training mean and standard deviation of channel k . The residuals $\varepsilon_{Ons}$ and $\varepsilon_{me}$ reported in Section 4.3 are the quantities of Equations 22 and 23, so the violation threshold of 0.05 used in Section 4.4 has an unambiguous meaning. Time reversal symmetry requires $\sigma_{ij}\left(B\right)=\sigma_{ji}\left(-B\right)$ . The sample is propagated once under B and once under −B , and the corresponding components are subtracted:

$$L_{Ons}={\left\|r\left({\hat{\sigma }}_{xy}\left(+B\right)\right)-r\left({\hat{\sigma }}_{yx}\left(-B\right)\right)\right\|}_{2}^{2}$$ (22)

The two arguments come from two forward passes differing only in the sign of the field channel of Equation 16. An explicit field input is essential: if the two passes were identical the residual would collapse to the difference between ${\hat{\sigma }}_{xy}$ and ${\hat{\sigma }}_{yx}$ at the same field, forcing the antisymmetric Hall part towards zero and suppressing the anomalous Hall response that this work aims to predict. Equality of the mixed second derivatives of the free energy with respect to the electric and magnetic fields requires the direct and converse magnetoelectric tensors to be transposes of one another, $\alpha_{ij}^{ME}=\alpha_{ji}^{EM}$ , a thermodynamic identity rather than an assumption of tensor symmetry. Channels 8 and 9 hold this conjugate pair, so

$$L_{me}={\left\|r\left({\hat{\alpha }}_{xy}^{ME}\right)-r\left({\hat{\alpha }}_{yx}^{EM}\right)\right\|}_{2}^{2}$$ (23)

The diagonal component $\alpha_{xx}$ satisfies the identity trivially and is not constrained. The two channels are labelled by two different measurements described in Section 4.1, which agree to within about 5%; the soft constraint drives the model outputs closer than the raw labels agree, and $\lambda_{me}$ controls how far that tightening proceeds before the data fit suffers. The data term is a componentwise weighted mean squared error that compensates for the differing magnitudes, as given in Equation 24:

$$L_{data}=\sum_{k}w_{k}{\left({\hat{Y}}_{k}-Y_{k}^{true}\right)}^{2}$$ (24)

where $Y_{k}^{true}$ is the true value of component k and $w_{k}$ is the reciprocal of its training standard deviation. The total loss is given by Equation 25:

$$L_{total}=L_{data}+\lambda_{Ons}L_{Ons}+\lambda_{me}L_{me}$$ (25)

with $\lambda_{Ons}$ and $\lambda_{me}$ determined by grid search on the validation set over $\left[10^{-3},10^{0}\right]$ . Because both constraint terms are dimensionless by construction, the interval carries the same meaning for every component, and the selected values are reported in Section 4.4. Figure 7 shows the prediction head and the constrained loss, with the sample propagated once under positive and once under negative field and the direct and converse magnetoelectric components paired within the same forward output. The constraints do not alter the architecture and act only through backpropagation. The training loop is given in Supplementary Section S14.

**FIGURE 7:**
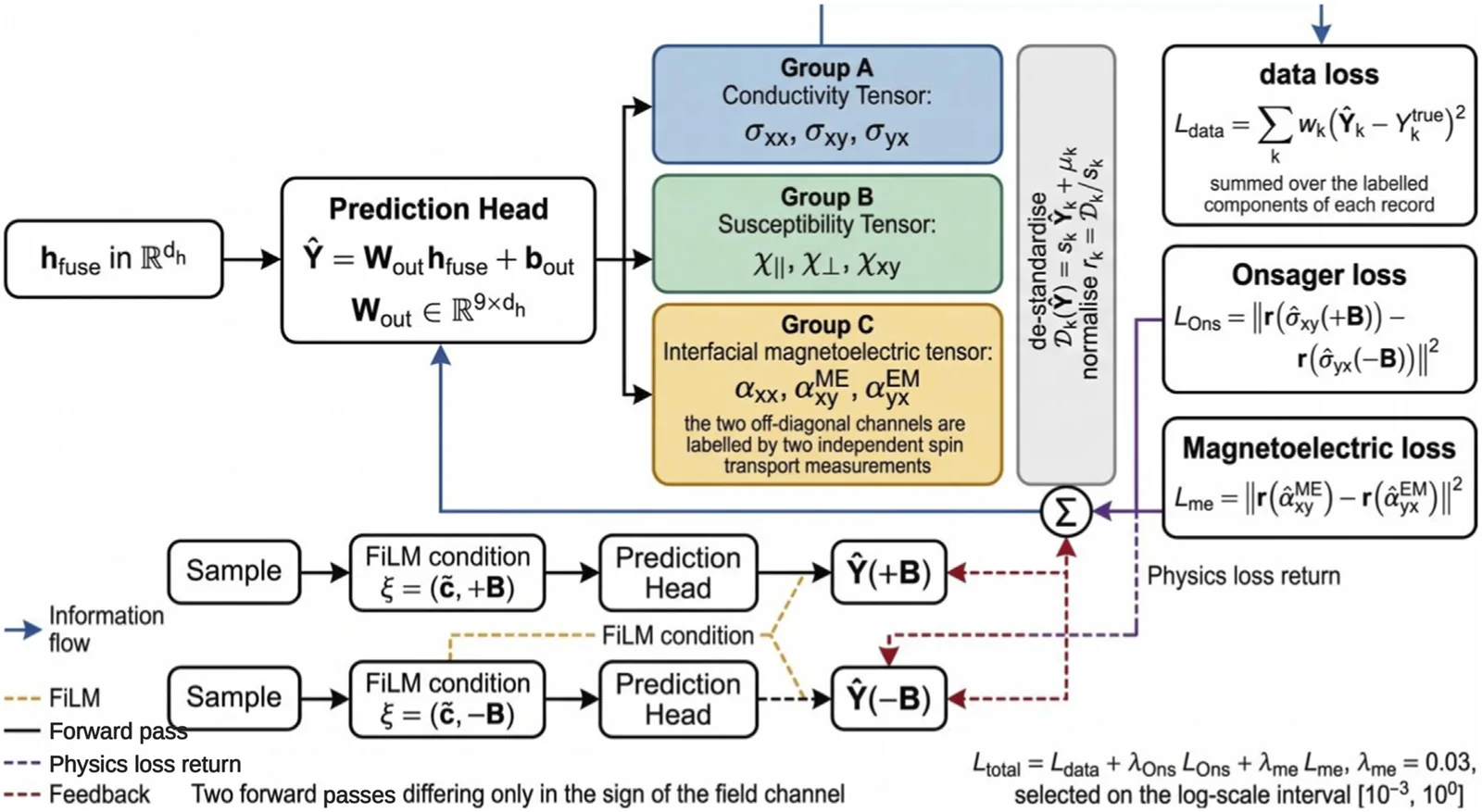
Joint prediction head with Onsager reciprocity and magnetoelectric reciprocity soft constraints.

Compared with hard constraints, the soft formulation neither alters the architecture nor requires redesigned equivariant layers, yet it makes the output of any backbone satisfy the symmetries, and the strength of physical consistency remains adjustable through $\lambda_{Ons}$ and $\lambda_{me}$ . This avoids compressing the search space into a zero measure subset while retaining physical support in extrapolation regions, and it remedies the separate prediction and concatenation paradigm criticized in Section 2.3. Together the four design elements give auditable input semantics, bidirectional information flow among scales, differentiable channel level process modulation, and physically consistent outputs, which support the validation and process inversion that follow.

## 4 Experiments and results

### 4.1 Dataset construction and experimental settings

The training data consist of electromagnetic response tensors from density functional calculations and from measurements on hot pressed samples. The DFT branch covers nine In:Pb ratios from 0.25:0.75 to 0.75:0.25, three interface orientations, (100)/(100), (110)/(110) and (111)/(111), four reaction layer thicknesses of 0, 2, 4 and 6 nm, and three defect density levels, which define 324 base configurations. Each is instantiated with three independent random realizations of the defect arrangement, giving 972 candidate structures, and structures that failed to converge or relaxed away from the intended geometry were discarded. The discard rate rises with reaction layer thickness, so the retained set of 800 records is weighted towards thinner layers. The experimental branch comprises 120 published records and 280 hot pressing experiments conducted by the research group over 100 °C–190 °C, 20–100 MPa, 5–60 min and 5–50 μm. A published record was retained only when the source reports the reaction layer thickness or a micrograph giving the grain boundary density by line intercept, together with a pole figure or XRD texture coefficients giving the texture index. The dataset contains 1,200 samples, as summarized in Table 3. Descriptive statistics and the complete record of the 280 in-house experiments are listed in Supplementary Tables S9 and S10.

**TABLE 3:** Composition, scale provenance and field coverage of the MSCA-EMNet dataset.

Data source	Number of samples	Main variable range	Applied field	Atomic block	Interfacial block	Mesoscale block	Processing block
DFT plus SOC first principles calculations	800	Stoichiometric ratio, interface orientation, reaction layer thickness	External Zeeman term at ±0.5, ±1, ±2, ±3 or ±5 T, assigned in equal proportion	Computed	Computed	Defined by the supercell, $\rho_{gb}$ = 0	Absent, $m_{c}$ = 0
Published experimental data	120	Conventional hot pressing combinations	As reported, binned to the nearest of the five magnitudes	Transferred by orientation weighted interpolation	Transferred by orientation weighted interpolation	Measured or recovered from reported micrographs	Reported
Hot pressing experiments conducted by the research group	280	T ∈[100,190] °C, P ∈[20,100] MPa	Swept from −5 to +5 T, record taken at the assigned magnitude	Transferred by orientation weighted interpolation	Transferred by orientation weighted interpolation	Measured	Measured
Total	1,200	—	—	—	—	—	—

All first principles calculations used the plane wave total energy scheme (Kresse and Furthmüller, 1996) with projector augmented wave potentials (Blöchl, 1994), the PBE generalized gradient approximation (Perdew et al., 1996), a 500 eV cutoff, a Gamma centred 12 × 12 × 12 mesh for bulk cells and 12 × 12 × 1 for interface slabs, convergence criteria of 1 × 10^−6^ eV and 0.01 eV·Å^‒1^, and non collinear second variational spin-orbit coupling. Interface slabs are coincidence site lattice supercells of 128–192 atoms with a lattice mismatch below 3%, a 15 Å vacuum layer and a dipole correction. The full protocol is listed in Supplementary Table S12 and the input file templates are reproduced in Supplementary Section S8.

Every record is defined at one assigned field magnitude from 0.5, 1, 2, 3 and 5 T, assigned in equal proportion within the first principles and in-house branches, while published records take the reported field binned to the nearest value, so the model is conditioned on a field varying in magnitude as well as sign. For hot pressed specimens the longitudinal conductivity is measured at zero field, the susceptibility at 0.05 T, and the transverse components over the full sweep from −5 to +5 T under the standard field reversal protocol, which confirms linearity; only the antisymmetrized pair at the assigned magnitude enters the record, so the pair required by Equation 22 comes from measurement rather than assumption. For first principles records the field enters as an external Zeeman term $\mu_{B}B$ in the non collinear Hamiltonian, and reversing its sign reverses the field induced spin polarization, so the two members of a pair are distinct self consistent solutions. An applied field is indispensable because In-Pb is nonmagnetic and a zero field calculation carries no time reversal breaking and returns a vanishing transverse conductivity. The transverse conductivity is the sum of the intrinsic Berry curvature term under the Zeeman field and the ordinary Lorentz term from semiclassical Boltzmann transport on the same Wannier interpolated bands, both odd in the field. A configuration evaluated at the two opposite fields counts as one record.

The direct and converse magnetoelectric coefficients are labelled by separate measurements, so the two channels compared in Equation 23 carry independent information. On the first principles side a finite electric field of 0.01 V·Å^‒1^ normal to the interface with a dipole correction gives the converse coefficient from the induced magnetization integrated within 1 nm of the boundary, and the polarization induced by the Zeeman perturbation gives the direct coefficient by the Berry phase route. On the experimental side a 5 nm Ni_80_Fe_20_ detection layer is sputtered onto each polished bar; the converse coefficient follows from the field-like effective field measured by second harmonic Hall analysis, which probes charge to spin conversion, calibrated once on a Pt (5 nm)/Ni_80_Fe_20_(5 nm) reference stack from the same run (Kim et al., 2023), and the direct coefficient from the charge voltage generated by spin pumping under ferromagnetic resonance, which probes the reverse process on the same specimen. The two routes agree to within about 5%, which sets the noise floor for the reciprocity residual. The magnetoelectric channels of the 120 published records are unlabelled, so a component level label mask accompanies each record and the data term of Equation 24 is summed only over labelled components, leaving 1,080 labelled records.

The two branches do not carry identical scale coverage, and the alignment follows an explicit procedure. The atomic and interfacial descriptors of the 400 experimental records are transferred from the first principles grid by orientation weighted interpolation rather than a nearest neighbour lookup, because a hot pressed specimen carries an orientation distribution rather than one interface plane. EBSD and STEM return the volume fractions of the three orientation archetypes, and the descriptor is their fraction weighted average, each value obtained by linear interpolation in composition ratio and reaction layer thickness and nearest neighbour assignment in defect level, with composition measured by ICP and reaction layer thickness by HAADF imaging, so every entry traces back to a measured quantity. The mesoscale block of the first principles records is defined by the supercell itself, since the texture index follows from the orientation pair, the thickness is a construction parameter, and the grain boundary density of a defect free bicrystal is zero. The processing block is the only genuinely absent block; these records carry $m_{c}$ = 0 and their four processing components are replaced by the learnable absent process embedding of Equation 2, so they contribute no gradient to the processing dependent parameters. Four hundred records therefore carry a value in every block and eight hundred in three of them, with the provenance listed in Table 3.

A stratified random split gives 840 training, 180 validation and 120 interpolation test samples, with a further 60 samples at sparsely populated processing edges retained as an extrapolation subset. Stratification has two levels. The data source defines the first, because the branches differ in their available stratification variables. Within the experimental branch the second level is the hot pressing temperature interval, which prevents dilution of the high temperature region, and within the first principles branch it is the interface orientation, since those records carry no temperature label. The test subsets are drawn only from the first principles records and the in-house experiments, because the published records carry less complete metadata and unlabelled magnetoelectric channels. Stratum counts are in Supplementary Table S11. The extrapolation combinations are absent from training and validation. They are defined by $D_{50}$ <8 μm, T >180 °C or P >90 MPa, inside the outer bounds of the processing space but in sparsely sampled regions, so the subset measures generalization to held out sparse regions rather than to conditions beyond the equipment range.

Seven baselines are used: CGCNN, ALIGNN, CrabNet, MEGNet, an equivariant tensor graph network, Equiv-GNN, a plain multilayer perceptron and a gradient boosted tree ensemble. The last two are included because the input space reduces to thirteen scalars plus the applied field, a regime in which tree ensembles remain competitive with deep architectures (Shwartz-Ziv and Armon, 2022), so a comparison restricted to graph and composition models would leave open whether any advantage comes from architecture or from input availability. The graph baselines receive a crystal graph of the representative supercell plus the three mesoscale scalars, the four processing scalars and the field, concatenated with the pooled embedding before the readout layer or placed in the global state vector; CrabNet receives the composition embedding with the same vector; the perceptron and the tree ensemble receive all fourteen scalars directly. Every model therefore sees the same information, and Supplementary Table S13 records the delivery of each block. All baselines are retrained on the same dataset with hyperparameters optimized by Optuna with the tree structured Parzen estimator over 200 trials. Metrics are nMAE, $R^{2}$ , and the Onsager and magnetoelectric reciprocity residuals defined by Equations 22 and 23 on destandardized values. Training uses one NVIDIA A100 GPU, PyTorch 2.1, batch size 64, Adam at 1 × 10^−3^, cosine annealing and 150 epochs; the perceptron has hidden layers of 512, 256 and 128 with ReLU and dropout 0.1, and the tree ensemble 800 trees of depth 6 at a learning rate of 0.05. All experiments are repeated five times with different seeds and both mean and standard deviation are reported, with differences assessed by a paired two sided t-test on the per sample absolute errors of the 120 interpolation test samples and a Wilcoxon signed rank check at the 0.05 level. Each component is standardized separately on the training set before nMAE is computed.

### 4.2 Comparison of joint prediction performance for the electromagnetic coupling tensor

MSCA-EMNet is substantially more accurate than the baselines. The overall nMAE across the nine components is 0.041, about 43% below the 0.072 of the strongest baseline, Equiv-GNN, and the overall $R^{2}$ rises from 0.884 to 0.948. The gradient boosted tree ensemble reaches 0.073 on exactly the same fourteen scalars, on a par with the strongest graph baseline, so the advantage does not come from input availability. Figure 8 compares predicted and true values for all eight models. MSCA-EMNet lies almost entirely along the y=x band whereas the baselines underestimate systematically in the high value region, the main deviations following from the large dynamic range of the longitudinal conductivity rather than from instability.

**FIGURE 8:**
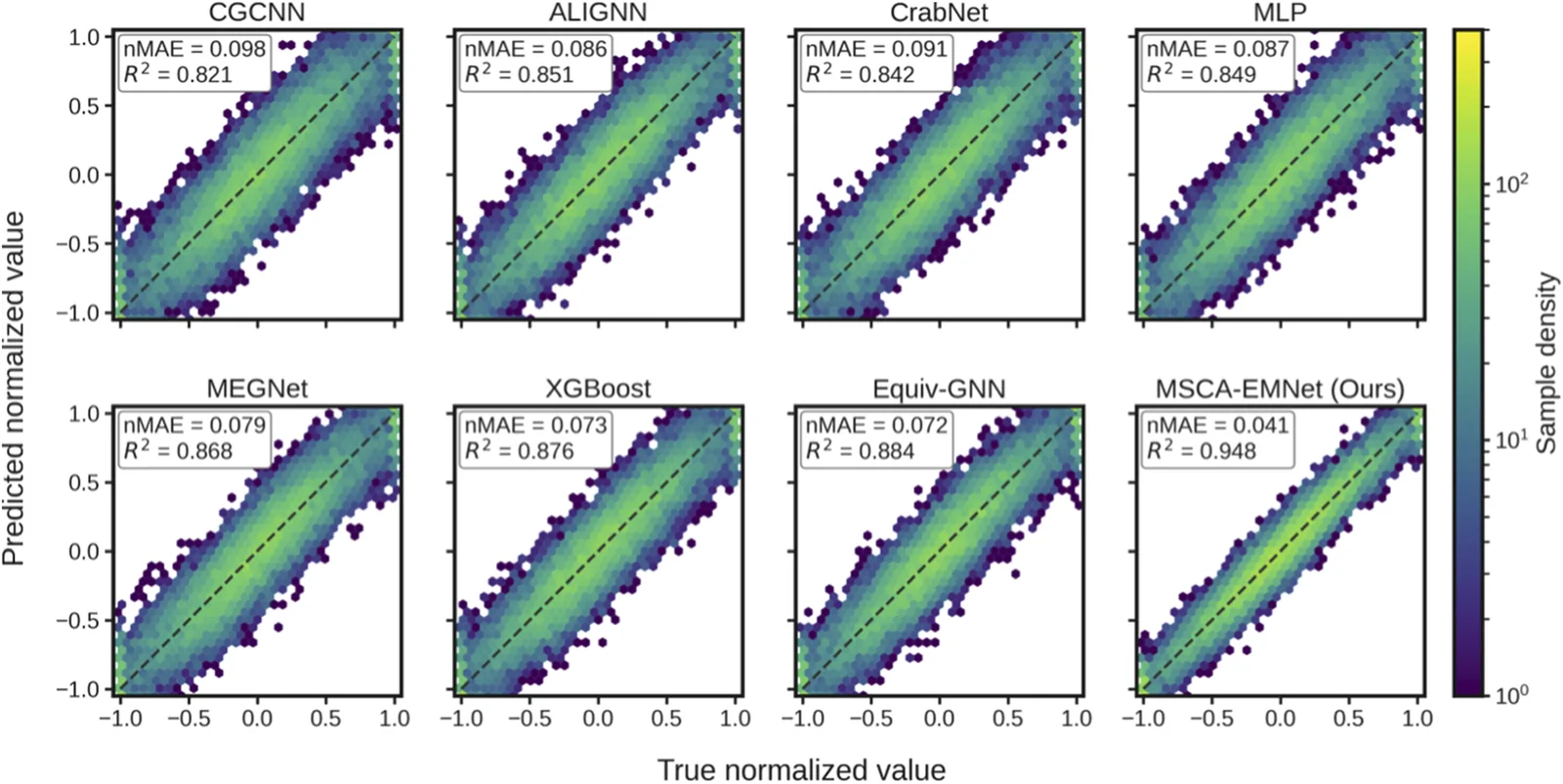
Hexagonal density scatter plots of nine tensor components predicted by MSCA-EMNet and the baseline models on the test set.

Component level results are given in Table 4. The distribution of $\sigma_{xx}$ is the most concentrated, so all models do well and MSCA-EMNet reaches 0.029, whereas $\sigma_{xy}$ depends on strong SOC and the anomalous Hall mechanism and the baseline errors rise markedly while MSCA-EMNet holds 0.043. The three interfacial magnetoelectric components are the hardest family: baseline $R^{2}$ for $\alpha_{xy}^{ME}$ ranges from 0.757 to 0.842 while MSCA-EMNet reaches 0.929 through the magnetoelectric reciprocity soft constraint, so the advantage grows as the coupling becomes stronger. The standard deviation over five runs does not exceed 0.007 in nMAE and 0.015 in $R^{2}$ for any component of any model, and the paired t-test gives p <0.001 for MSCA-EMNet against Equiv-GNN and against XGBoost on all nine components, with the Wilcoxon test agreeing; per component values are listed in Supplementary Tables S1 and S2. All test samples carry magnetoelectric labels, since the unlabelled published records are confined to training and validation.

**TABLE 4:** Prediction performance of MSCA-EMNet and the baseline models on the test set, nMAE/ $R^{2}$ .

Model	$\sigma_{xx}$	$\sigma_{xy}$	$\sigma_{yx}$	$\chi_{∥}$	$\chi_{⊥}$	$\chi_{xy}$	$\alpha_{xx}$	$\alpha_{xy}^{ME}$	$\alpha_{yx}^{EM}$	Overall
CGCNN	0.069/0.896	0.103/0.803	0.100/0.810	0.078/0.860	0.081/0.853	0.093/0.832	0.116/0.782	0.125/0.757	0.117/0.796	0.098 ± 0.005/0.821 ± 0.011
ALIGNN	0.060/0.914	0.090/0.836	0.088/0.842	0.069/0.884	0.071/0.878	0.082/0.860	0.101/0.818	0.110/0.797	0.102/0.830	0.086 ± 0.004/0.851 ± 0.009
CrabNet	0.064/0.908	0.096/0.826	0.093/0.833	0.073/0.877	0.076/0.870	0.086/0.851	0.107/0.807	0.117/0.785	0.108/0.820	0.091 ± 0.004/0.842 ± 0.010
MLP	0.061/0.912	0.091/0.834	0.089/0.840	0.070/0.882	0.072/0.876	0.083/0.858	0.103/0.816	0.111/0.795	0.104/0.828	0.087 ± 0.005/0.849 ± 0.012
MEGNet	0.055/0.923	0.083/0.855	0.081/0.860	0.063/0.897	0.066/0.892	0.075/0.876	0.093/0.839	0.101/0.820	0.094/0.850	0.079 ± 0.003/0.868 ± 0.008
XGBoost	0.051/0.928	0.077/0.864	0.074/0.869	0.058/0.903	0.061/0.898	0.069/0.883	0.086/0.849	0.093/0.831	0.087/0.859	0.073 ± 0.002/0.876 ± 0.006
Equiv-GNN	0.050/0.933	0.076/0.872	0.073/0.877	0.058/0.910	0.060/0.905	0.068/0.891	0.085/0.858	0.092/0.842	0.086/0.868	0.072 ± 0.003/0.884 ± 0.007
MSCA-EMNet	0.029/0.970	0.043/0.943	0.042/0.945	0.033/0.959	0.034/0.957	0.039/0.951	0.048/0.937	0.053/0.929	0.049/0.941	0.041 ± 0.002/0.948 ± 0.004

Table 4 also shows that the baselines differ little between conductivity and susceptibility components but decline sharply for the magnetoelectric family, reflecting the flattening of their internal electrical and magnetic channels. The decline is much smaller for MSCA-EMNet, so the bidirectional coupling of BS-CrossAttn connects the two families, and the $R^{2}$ advantage over the baselines widens for $\sigma_{xy}$ and $\alpha_{xy}^{ME}$ , the components tied to SOC and the anomalous Hall effect. For the 27 test samples with $\delta_{rl}$ of 4 nm or more the baselines give residuals above three times the test set mean, whereas MSCA-EMNet limits these to no more than three samples.

### 4.3 Ablation experiments on key modules

To quantify the contribution of each module, ten ablation variants are built: removal of BS-CrossAttn, which leaves a unidirectional upward model; removal of FiLM, with processing parameters concatenated to the input; removal of the hierarchical encoder, with all four categories sharing one MLP; removal of the Onsager and magnetoelectric reciprocity constraints; removal of the macroscopic token from the downward branch; removal of the first principles branch, which restricts training to the 200 hot pressing records of the training partition; three depth variants with $L_{bs}$ = 1, 3 and 4; and simultaneous removal of all four components as the minimal baseline. All variants keep the same parameter count and training strategy except the depth variants, whose count scales with $L_{bs}$ . Results are in Table 5.

**TABLE 5:** Ablation experiments on key modules using the test set.

Ablation variant	Overall nMAE	$R^{2}$	$\varepsilon_{Ons}$	$\varepsilon_{me}$	p against the full model
Full MSCA-EMNet model	0.041 ± 0.002	0.948 ± 0.004	0.024 ± 0.002	0.031 ± 0.003	—
Without BS-CrossAttn, upward aggregation only	0.055 ± 0.003	0.912 ± 0.007	0.036 ± 0.003	0.044 ± 0.004	<0.001
Without FiLM, process concatenation	0.063 ± 0.004	0.897 ± 0.009	0.041 ± 0.003	0.049 ± 0.004	<0.001
Without hierarchical encoder, unified MLP	0.058 ± 0.003	0.902 ± 0.008	0.038 ± 0.003	0.046 ± 0.004	<0.001
Without physical constraints, data term only	0.046 ± 0.002	0.938 ± 0.005	0.152 ± 0.011	0.187 ± 0.014	0.004
Without the macroscopic token in the downward branch	0.048 ± 0.003	0.933 ± 0.006	0.029 ± 0.002	0.036 ± 0.003	0.006
Without the first principles branch, hot pressing records only	0.058 ± 0.004	0.903 ± 0.010	0.033 ± 0.003	0.041 ± 0.004	<0.001
BS-CrossAttn with $L_{bs}$ = 1	0.047 ± 0.003	0.936 ± 0.006	0.028 ± 0.002	0.035 ± 0.003	0.009
BS-CrossAttn with $L_{bs}$ = 3	0.042 ± 0.002	0.946 ± 0.004	0.025 ± 0.002	0.032 ± 0.003	0.31
BS-CrossAttn with $L_{bs}$ = 4	0.044 ± 0.003	0.943 ± 0.005	0.026 ± 0.002	0.033 ± 0.003	0.12
Minimal baseline, all four components removed	0.089 ± 0.005	0.834 ± 0.011	0.191 ± 0.013	0.226 ± 0.016	<0.001

Three conclusions follow. Removing FiLM causes the largest loss in overall nMAE, an increase of 54%, so processing conditions are the central bridge of the framework and reducing them to an ordinary input vector costs the ability to modulate mesoscale texture. Removing the physical constraints raises nMAE by only 12% but increases $\varepsilon_{Ons}$ about 6.3 times and $\varepsilon_{me}$ about 6 times, confirming that the soft constraints govern physical consistency rather than data fitting. A unidirectional BS-CrossAttn raises nMAE by 34% with simultaneous increases in both residuals, so bidirectional interaction maintains coordination among tensor components.

Two further variants address the two data branches. Removing the macroscopic token raises the overall nMAE from 0.041 to 0.048, a moderate loss because FiLM still carries the processing signal, confirming that the downward attention path supplies information channel level modulation does not. Removing the first principles branch raises it to 0.058, an increase of 41%, so the 640 calculated training records contribute a measurable gain rather than merely enlarging the sample. Perturbing the transferred descriptors with uniform relative noise gives 0.042 at ±5%, 0.044 at ±10% and 0.049 at ±20%, so the cross scale result degrades smoothly and does not depend on their exact values. One BS-CrossAttn layer is insufficient while three and four give no significant improvement over two at p = 0.31 and p = 0.12, justifying $L_{bs}$ = 2. Complete results are in Supplementary Table S4.

Figure 9 compares the variants across overall nMAE, $R^{2}$ , $\varepsilon_{Ons}$ , $\varepsilon_{me}$ and extrapolation robustness. The robustness axis uses each variant’s degradation rate on the 60 extrapolation samples, listed in Supplementary Table S3. The five indicators differ in unit and direction, so each is mapped to a score: for a lower is better indicator the score is the distance from the worst variant divided by the best to worst spread, and the definition is reversed otherwise, so every axis runs from 0 at the worst to 1 at the best and a larger radius always denotes better performance. The reference set is the full model and the ten variants of Table 5. The full model is outermost on all five axes. The variant without physical constraints keeps accuracy close to the full model but contracts sharply on the consistency axes, and among the single module removals the variant without FiLM contracts most on extrapolation robustness.

**FIGURE 9:**
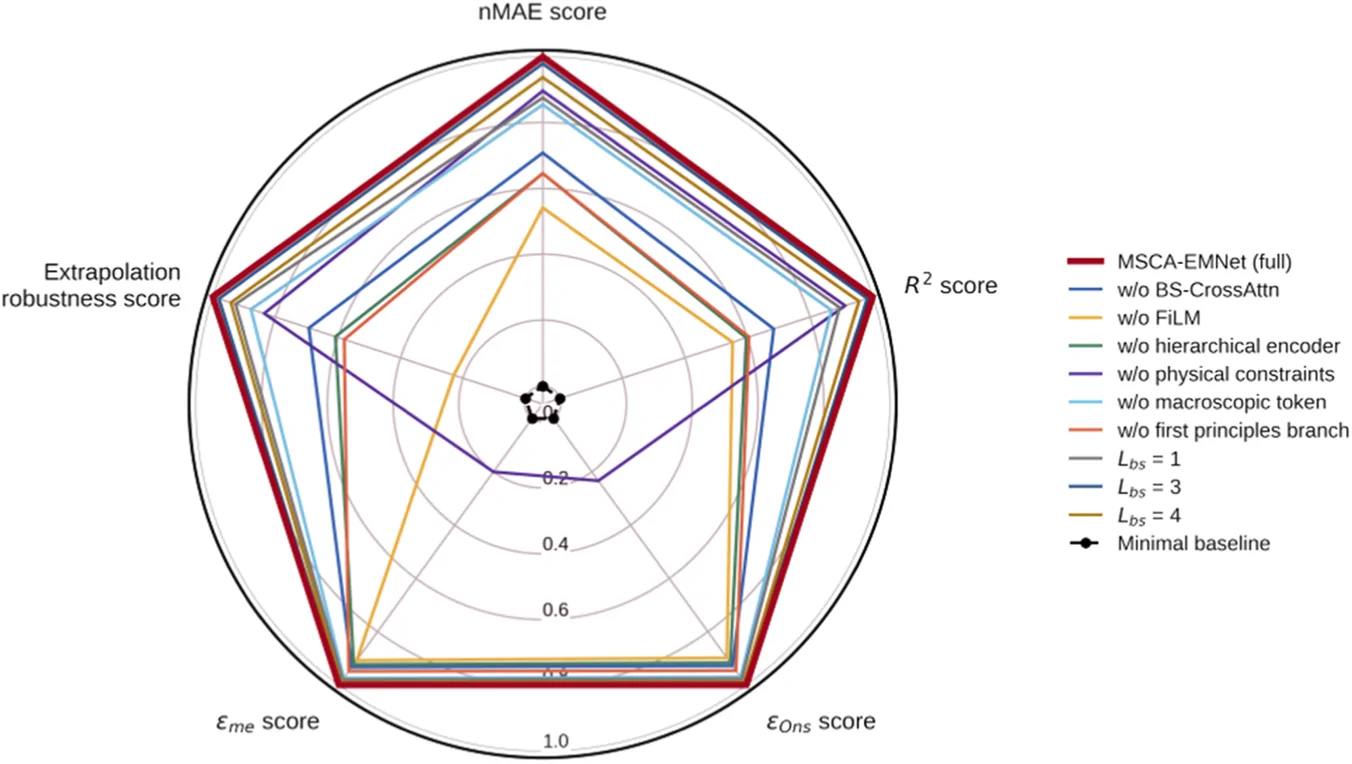
Radar chart comparison of the full model and the ten ablation variants across five normalized score indicators.

Figure 4 presents attention maps for a representative sample at layer 2. Rows are query tokens, columns key tokens, and every row sums to unity because the softmax of Equation 9 acts on the key token axis. Because Key and Value are restricted to adjacent scales, the figure separates the two adjacent maps from the two composed maps of Equation 15: the 3 × 3 upward map between interfacial and atomic tokens, the 3 × 5 downward map from mesoscale queries onto macroscopic tokens, the composed 3 × 3 atomic to mesoscale map, and the composed 3 × 5 macroscopic to atomic map. The composed panels carry the cross scale reading, because no single entry connects two non adjacent levels. In the composed upward map the weights concentrate between the Pb-6p SOC strength and the EBSD texture index, and in the composed downward map between the hot pressing temperature and Pb-6p SOC, consistent with the observed law that temperature modulates the coupling between atomic layer SOC and interfacial reactions.

### 4.4 Sensitivity analysis and robustness evaluation

Processing parameter sensitivity is analysed with Sobol’ indices. They are estimated by Saltelli sampling on a base sample of 1,024 points, giving 1,024 × ( 2k +2) evaluations for k = 4 at the reference field of 2 T, with confidence intervals from 1,000 bootstrap resamples (Razavi et al., 2021). First order and total order indices are reported together, because first order indices alone do not close to unity when interactions are present and their deficit is exactly the interaction share. Table 6 gives five representative components. Temperature contributes most to $\sigma_{xx}$ , 37.1%, consistent with its control of consolidation density. Particle size contributes more than 32% to both principal susceptibility components through grain boundary density. Pressure contributes evenly, whereas holding time contributes more to $\sigma_{xy}$ (16.0%) than to $\sigma_{xx}$ (11.1%), consistent with reaction layer thickening and enhanced interfacial SOC. First order indices sum to between 87.9% and 91.1% and the remainder is carried by interactions, the two largest listed explicitly. The 95% bootstrap half width does not exceed 1.4 percentage points for any first order index and 2.1 points for any total order index, so the parameter ordering is resolved for every component.

**TABLE 6:** First order, second order and total order Sobol’ indices of processing parameters, %.

Tensor component	$S_{T}$	$S_{P}$	$S_{t}$	$S_{D_{50}}$	Sum of first order	$S_{T,D_{50}}$	$S_{P,t}$	$S_{T}^{tot}$	$S_{P}^{tot}$	$S_{t}^{tot}$	$S_{D_{50}}^{tot}$
$\sigma_{xx}$	37.1	17.0	11.1	24.8	90.0	5.2	1.9	43.9	20.8	14.0	31.3
$\sigma_{xy}$	28.5	18.7	16.0	24.7	87.9	3.1	6.8	32.7	26.8	23.7	28.9
$\chi_{∥}$	22.6	18.3	17.8	32.3	91.0	3.4	2.6	27.3	22.5	21.8	37.4
$\chi_{⊥}$	20.6	19.5	17.2	33.8	91.1	3.0	2.7	24.9	23.9	21.4	38.7
$\alpha_{xy}^{ME}$	26.4	24.0	15.0	23.6	89.0	4.2	4.1	32.0	29.8	20.1	29.1

Figure 10 maps the predicted $\sigma_{xx}$ over the $\left(T,P\right)$ plane. The contours become dense near T = 170 °C, where density rises rapidly as partial melting of the In-rich phase sets in, and a second dense band appears near P = 60 MPa, the threshold at which the powder passes from particle contact to a continuous matrix. The surface saturates above T = 180 °C and P = 80 MPa, consistent with grain coarsening and the subsequent decline in conductivity.

**FIGURE 10:**
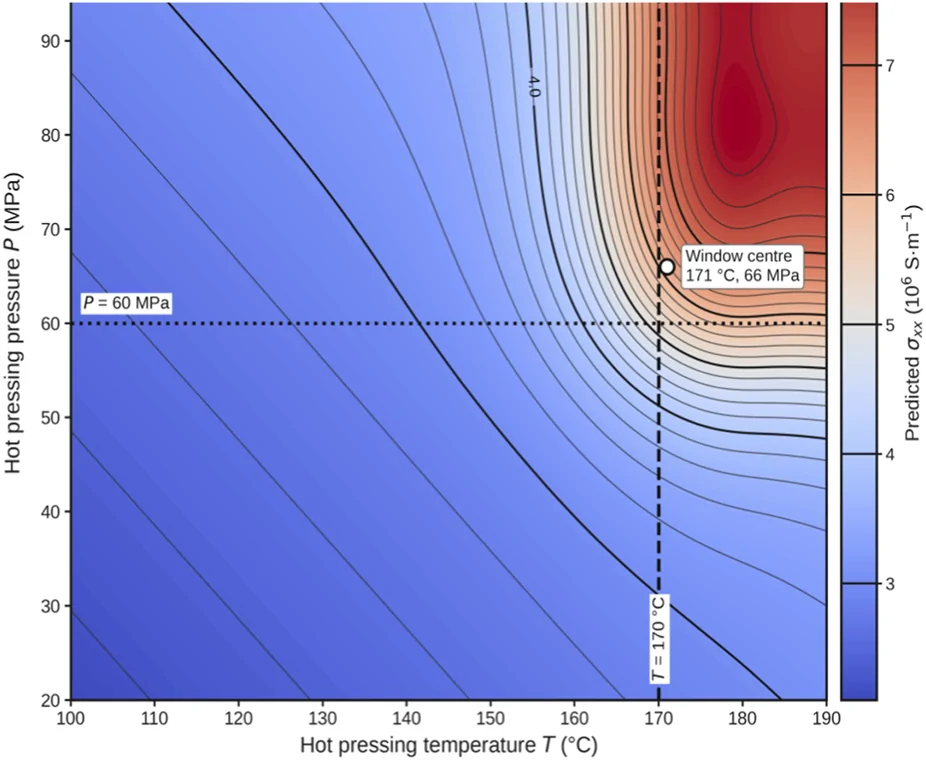
Contour prediction of $\sigma_{xx}$ in the $\left(T,P\right)$ processing plane.

Robustness is evaluated on the 60 extrapolation samples defined by $D_{50}$ <8 μm, T >180 °C or P >90 MPa. Figure 11 compares the nMAE distributions in the interpolation and extrapolation regions. The median extrapolation nMAE of MSCA-EMNet is 0.061, about 48% below the 0.118 of Equiv-GNN, and its distribution is far more compact, because the FiLM channel modulates every layer with the processing conditions and the physical constraints suppress outputs that violate Onsager or magnetoelectric reciprocity outside the training distribution.

**FIGURE 11:**
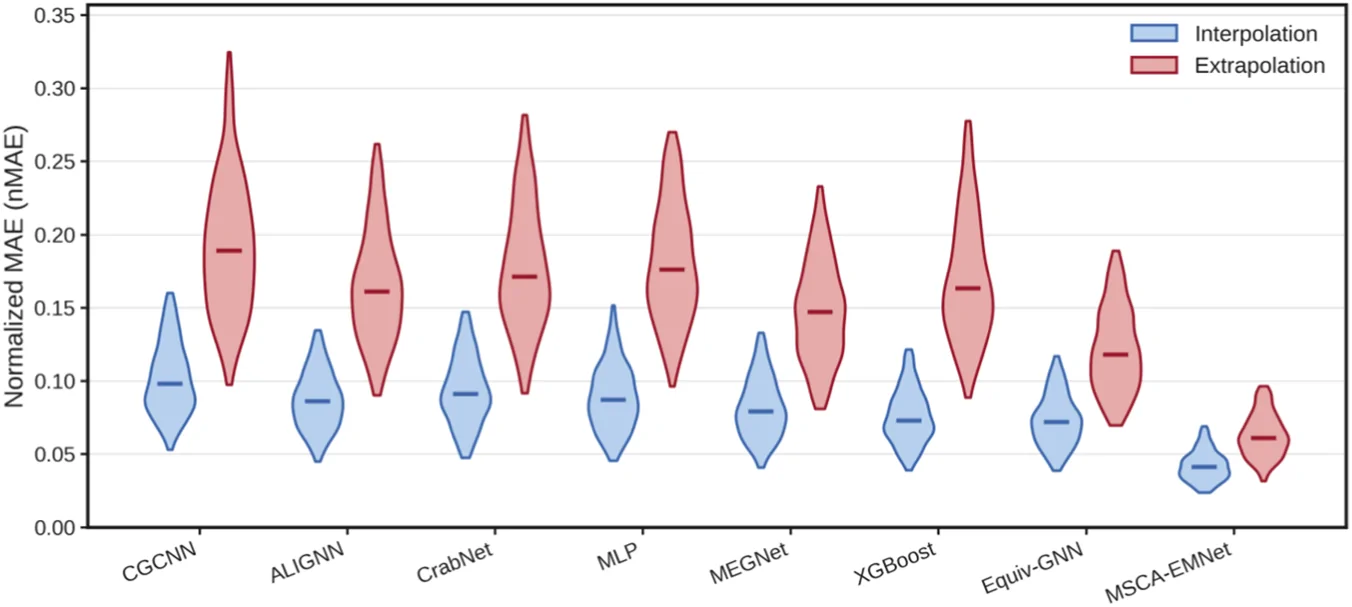
Violin plots of nMAE distributions in the interpolation and extrapolation regions.

The degradation rate in Table 7 is the extrapolation minus the interpolation nMAE divided by the interpolation nMAE. The mean of the five graph and composition based baselines is 83.6% and MSCA-EMNet reaches about 58% of it. The two tabular baselines degrade to 102.3% and 123.3%, so their competitive interpolation accuracy does not survive sparsely sampled regions: a tree ensemble partitions the space into axis aligned cells learned from the training distribution and has no mechanism for a corrected response outside them.

**TABLE 7:** Performance comparison in the extrapolation region using 60 extrapolation samples.

Model	Interpolation nMAE	Extrapolation nMAE	Extrapolation R_2_	Extrapolation degradation rate
CGCNN	0.098 ± 0.005	0.189 ± 0.011	0.628 ± 0.019	92.9%
ALIGNN	0.086 ± 0.004	0.161 ± 0.009	0.684 ± 0.016	87.2%
CrabNet	0.091 ± 0.004	0.171 ± 0.010	0.674 ± 0.018	87.9%
MLP	0.087 ± 0.005	0.176 ± 0.012	0.688 ± 0.021	102.3%
MEGNet	0.079 ± 0.003	0.147 ± 0.008	0.712 ± 0.014	86.1%
XGBoost	0.073 ± 0.002	0.163 ± 0.011	0.706 ± 0.017	123.3%
Equiv-GNN	0.072 ± 0.003	0.118 ± 0.006	0.783 ± 0.011	63.9%
MSCA-EMNet	0.041 ± 0.002	0.061 ± 0.003	0.892 ± 0.007	48.8%

Figure 12 presents a parallel coordinate plot of the nine components in the space of nMAE, $\varepsilon_{Ons}$ and $\varepsilon_{me}$ . The MSCA-EMNet curve group is compact and generally lower, so its outputs are both more accurate and markedly more physically consistent across components and can enter downstream device design without postprocessing.

**FIGURE 12:**
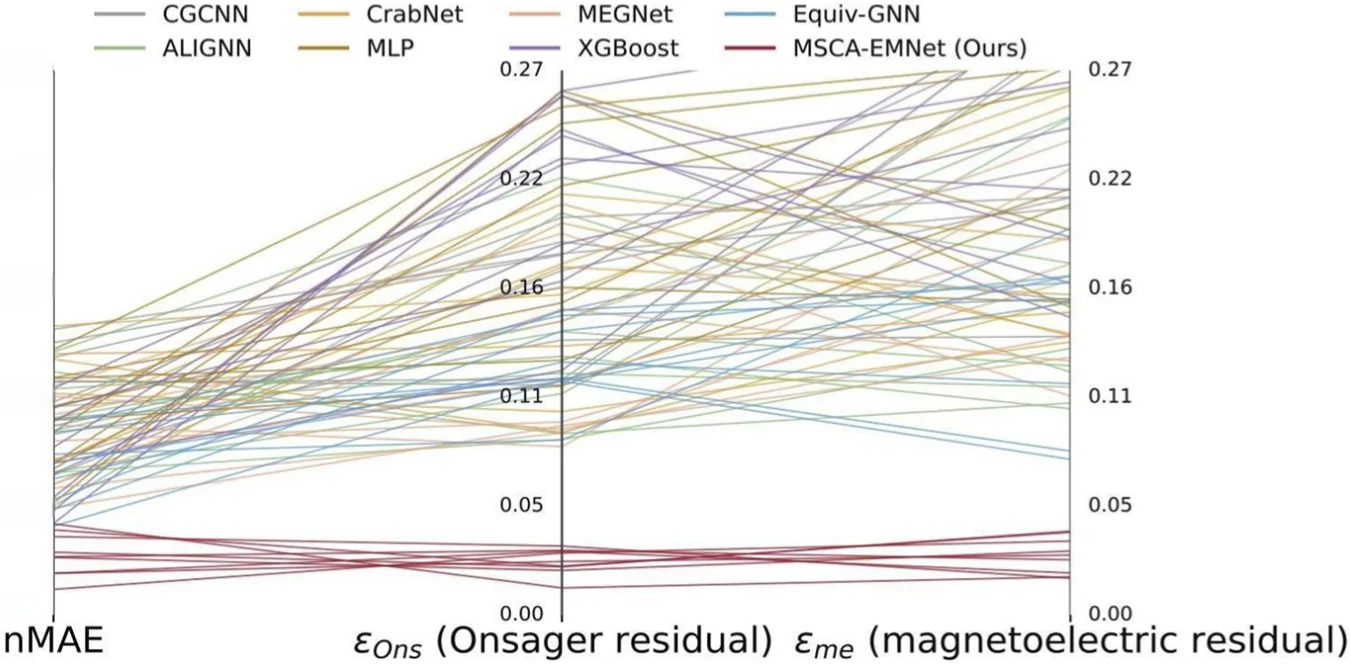
Parallel coordinate plot of the nine component tensors predicted by different methods in the space of nMAE, Onsager residual, and magnetoelectric residual.

Only 1.7% of the MSCA-EMNet test samples violate the Onsager relation, defined as a residual above 0.05, against 12.4% for Equiv-GNN, 21.6% for MEGNet, 33.8% for the multilayer perceptron and 41.2% for the tree ensemble, the tabular models occupying the upper end because neither evaluates the response at +B and −B within a single training step, so nothing couples the two conjugate transverse components. Two coupling patterns also emerge. Temperature and particle size interact significantly, with a second order index of 5.2% for $\sigma_{xx}$ : below 15 μm the derivative of $\sigma_{xx}$ with respect to temperature is much larger than above 30 μm, as smaller particles offer more specific surface area. Pressure and holding time interact most strongly for $\sigma_{xy}$ , reaching 6.8%, reflecting interfacial reaction kinetics set jointly by pressure and time.

The sensitivity to the two constraint weights is examined on the validation set over a logarithmic grid of seven values per weight from 1 × 10^−3^ to 1 with three repetitions per combination; the grid is shown in Figure 13 and its values are listed in Supplementary Table S5. Raising either weight from the lower end suppresses the corresponding residual sharply while leaving the data fit almost unchanged, and the validation nMAE varies by no more than 0.002 from 0.01 to 0.1. Beyond 0.1 the constraints compete with the data term and accuracy falls to 0.062 when both equal 1. The pair $\lambda_{Ons}$ = 0.03 and $\lambda_{me}$ = 0.03 gives the lowest validation nMAE and is adopted throughout. Because both residuals are dimensionless by the construction of Equation 21, the interval means the same for every component, and the broad plateau shows that the reported performance does not rest on a narrowly tuned pair.

**FIGURE 13:**
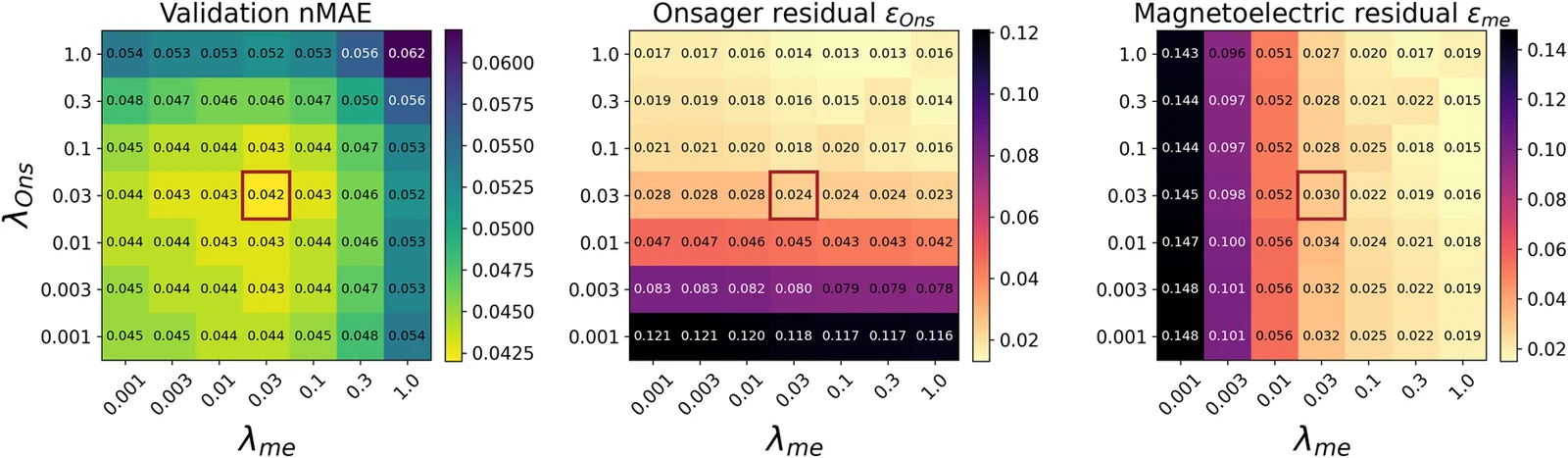
Validation nMAE and physical residuals over the grid of Onsager and magnetoelectric constraint weights.

### 4.5 Inverse design of the hot pressing window and experimental confirmation

The differentiable pathway established by FiLM lets the framework run in reverse. The target is chosen for a flexible electromagnetic shielding application requiring high longitudinal conductivity with a pronounced transverse response: $\sigma_{xx}$ = 6.20 × 10^6^ S·m^‒1^, $\sigma_{xy}$ = 6.50 × 10^3^ S·m^‒1^ and $\sigma_{yx}$ = ‒6.50 × 10^3^ S·m^‒1^ at B = 2 T, $\chi_{∥}$ = ‒1.28 × 10^−5^, $\chi_{⊥}$ = ‒1.14 × 10^−5^, $\chi_{xy}$ = 5.20 × 10^−7^, $\alpha_{xx}$ = 0.140 ps·m^‒1^, $\alpha_{xy}^{ME}$ = 0.680 ps·m^‒1^ and $\alpha_{yx}^{EM}$ = 0.680 ps·m^‒1^. Every entry lies inside the range of Table 2, so the target describes an attainable material.

The network is frozen and the processing vector is the only learnable variable, following Equation 18. The regularizer $R\left(c\right)$ is a squared hinge penalty on violation of T ∈[100,190] °C, P ∈[20,100] MPa and t ∈[5,60] min with $\lambda_{r}$ = 0.1, and $D_{50}$ is optimized continuously and then projected onto the available powder grades of 8, 10, 12, 15, 20, 25, 30, 40 and 50 μm. Optimization uses Adam at a learning rate of 0.01 for 2,000 steps from 100 random starts drawn uniformly in the feasible box.

All 100 starts converge into a single connected cluster rather than scattered minima, so the target corresponds to one contiguous window. It covers T = 168 °C–174 °C, P = 62–70 MPa, t = 25–35 min and $D_{50}$ = 8–12 μm, centred at 171 °C, 66 MPa, 30 min and 10 μm, and agrees with independent evidence collected earlier: the centre temperature sits just below the onset of partial melting of the In-rich phase and inside the dense contour band at T = 170 °C of Figure 10, the pressure lies above the 60 MPa densification threshold, the holding time corresponds to a reaction layer of about 3 nm, and the particle size falls below the 15 μm boundary at which the temperature and particle size interaction becomes pronounced in Table 6. The window therefore reproduces four process physics features never supplied to the optimizer.

Nine confirmatory hot pressings were carried out: six inside the window, at the centre, the four corners and one centre repeat, and three controls outside it at 155 °C, 45 MPa, 15 min and 30 μm and at two neighbouring settings. All were characterized by the in-house procedure and none of them entered the training, validation or test partitions; every specimen is listed in Supplementary Table S7. Table 8 compares the target, the prediction at the window centre and the measured mean of the six window specimens. The relative deviation does not exceed 7.6% on any channel, and the measured direct and converse magnetoelectric coefficients differ by 1.1%, well inside the mutual consistency of the two spin transport measurements, so the reciprocity constraint of Equation 23 is checked against separately acquired quantities. The controls deviate by 28.9% on $\sigma_{xx}$ , 39.1% on $\sigma_{xy}$ and 51.8% on $\alpha_{xy}^{ME}$ , confirming that the window boundary is meaningful. Figure 14 shows the feasible region with the converged solutions and the nine experiments, its colour field being the base ten logarithm of the normalized target mismatch of Equation 18.

**TABLE 8:** Target tensor, inverse solution and experimental confirmation of the predicted processing window.

Component	Target $Y^{*}$	Prediction at the window centre	Measured mean of the six window specimens	Relative deviation
$\sigma_{xx}$ (10^6^ S·m^‒1^)	6.20	6.17	6.04	2.6%
$\sigma_{xy}$ (10^3^ S·m^‒1^, B = 2 T)	6.50	6.44	6.13	5.7%
$\sigma_{yx}$ (10^3^ S·m^‒1^, B = 2 T)	−6.50	−6.41	−6.13	5.7%
$\chi_{∥}$ (10^−5^)	−1.280	−1.271	−1.216	5.0%
$\chi_{⊥}$ (10^−5^)	−1.140	−1.132	−1.085	4.8%
$\chi_{xy}$ (10^−7^)	5.20	5.15	4.83	7.1%
$\alpha_{xx}$ (ps·m^‒1^)	0.140	0.138	0.130	7.1%
$\alpha_{xy}^{ME}$ (ps·m^‒1^)	0.680	0.673	0.628	7.6%
$\alpha_{yx}^{EM}$ (ps·m^‒1^)	0.680	0.671	0.635	6.6%

**FIGURE 14:**
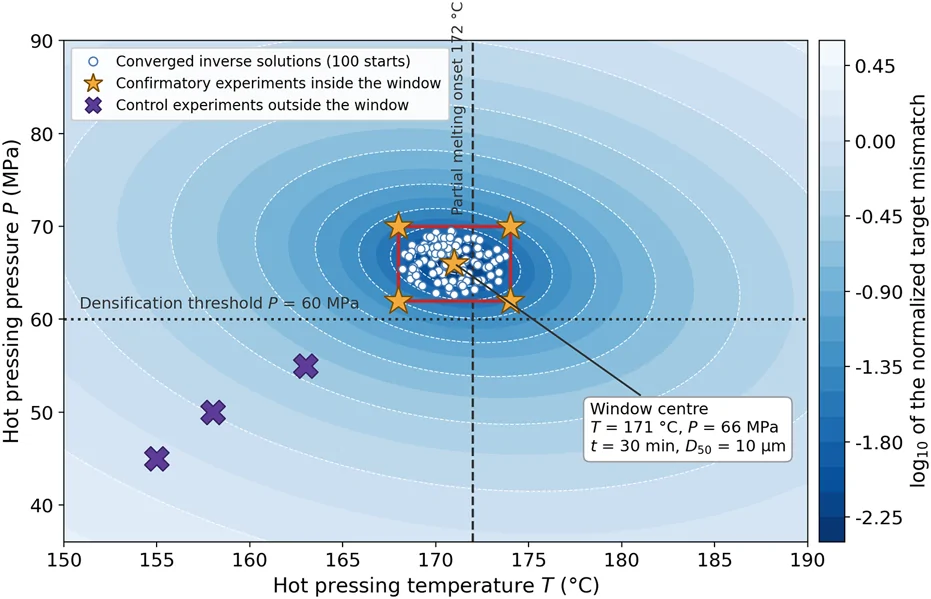
Feasible processing window in the $\left(T,P\right)$ plane with the converged inverse solutions and the confirmatory experiments.

## 5 Discussion

### 5.1 Physical interpretability of cross attention weights

After convergence the attention weights are highly interpretable. Figure 4 shows a pronounced high weight band in the composed upward map between the atomic Pb-6p SOC channel and the mesoscale EBSD texture channel. This is not an adjacent scale entry, since the upward branch connects atomic tokens only to interfacial tokens; it is an element of the composed matrix of Equation 15, so the coupling is a two step path through the interfacial electronic structure. It agrees with the mechanism by which Rashba splitting in low dimensional Pb based systems is sensitive to grain orientation: as grains approach a preferred plane, atomic layer SOC modulates the electronic structure more strongly and the weight concentrates between the two channels. In the downward branch, as the temperature rises from about 100 °C towards the onset of partial melting of the In-rich phase at approximately 172 °C, the weight between T and the Pb-6p SOC channel grows, reflecting indirect modulation of interfacial SOC through the reaction layer thickness. This weight is likewise a composed element, here the product of three adjacent transfers, so the process to atomic coupling necessarily runs through the mesoscale texture and the interfacial electronic structure rather than a direct shortcut.

The degree of concentration is summarized by the attention concentration index η , the normalized entropy deficit of the attention rows, as defined in Equation 26:

$$\eta =1-\frac{1}{L_{bs}N_{q}}\sum_{l=1}^{L_{bs}}\sum_{q=1}^{N_{q}}\frac{H\left(a_{l,q}\right)}{\ln n_{key}},H\left(a_{l,q}\right)=-\sum_{j}a_{l,q,j}\ln a_{l,q,j}$$ (26)

where $a_{l,q}$ is the attention row of query token q at layer l , $n_{key}$ the number of key tokens and $N_{q}$ the total number of query tokens across both branches. The index is 0 for uniform rows and approaches 1 for one-hot rows, and it is evaluated on the adjacent matrices rather than the composed ones because only the adjacent matrices come directly from a softmax and carry a well defined entropy. Averaging over both branches and tracking the index over training epochs gives Figure 15: η starts at 0.14, reaches 0.62 by epoch 50 and 0.71 by epoch 90, then stays flat. The rapid rise and plateau indicate that the model identifies physically correlated channels on its own and concentrates weight on meaningful coupling paths. This gives channel level interpretability relatable to experimental knowledge and explains the advantage over the baselines for the interfacial magnetoelectric component $\alpha_{xy}^{ME}$ , whose representation models the three level chain from process to microstructure to electronic structure instead of compressing all three into one embedding.

**FIGURE 15:**
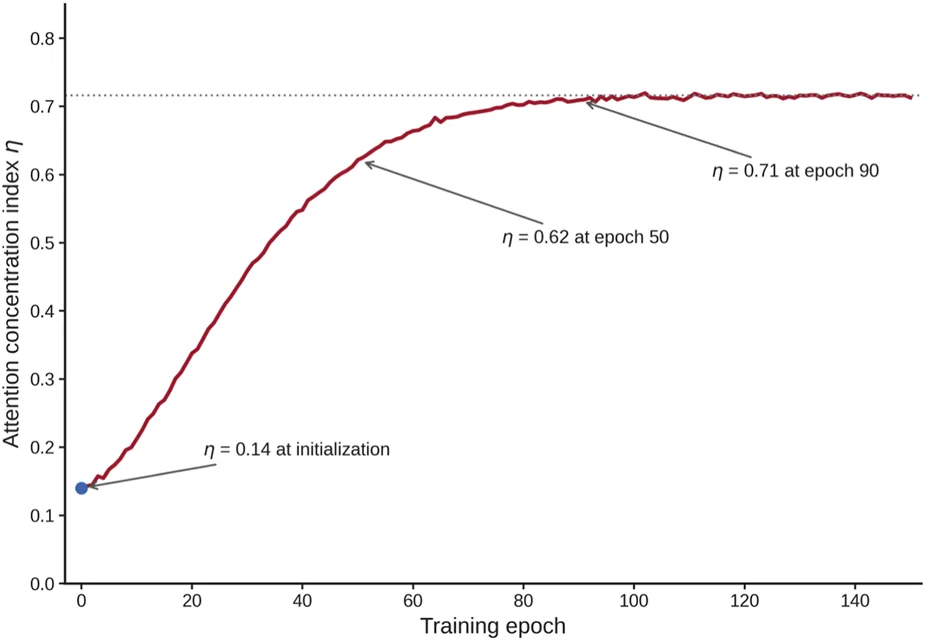
Evolution of the attention concentration index η during training.

### 5.2 Relationship between electrical and magnetic coupling patterns and low dimensional interfacial microstructural features

Combining the Sobol’ analysis of Section 4.4 with the attention weights reveals a hierarchical modulation law. Particle size contributes more than 32% to both principal susceptibility components, consistent with the classical picture in which particle size sets grain boundary density and grain boundary density regulates magnetic scattering, and it corresponds to the high weight coupling between the grain boundary density query and the key in the adjacent downward map of Figure 4. Temperature contributes as much as 37% to the longitudinal conductivity because consolidation density rises rapidly near the onset of partial melting of the In-rich phase, which matches the dense contour band at T = 170 °C in Figure 10, so the model captures the temperature window of the abrupt property change.

The anomalous Hall component behaves differently: the contribution of holding time rises to 16.0% with the particle size contribution at 24.7%, indicating the chain in which the reaction layer thickens with holding time and interfacial SOC is amplified as it thickens. The baselines lose this path because they either flatten the processing parameters into a segment of the input vector or ignore the bridge between mesoscale texture and the reaction layer, whereas MSCA-EMNet binds it into the architecture through the downward branch and the FiLM modulation. The same chain accounts for the interfacial magnetoelectric family, whose first order sensitivity is distributed almost evenly between temperature and pressure, the signature expected of a quantity that depends on how completely individual interface contributions cancel under polycrystalline averaging, since temperature and pressure jointly set the preferred orientation developed during consolidation. Within the dataset the predicted $\alpha_{xy}^{ME}$ grows monotonically with the EBSD texture index at fixed composition and is largest in the strongly textured specimens, in agreement with Section 3.5.

### 5.3 Cross system transfer and extrapolative generalization capability

Once samples enter sparse regions with $D_{50}$ <8 μm or T >180 °C the degradation rate of MSCA-EMNet is 48.8%, whereas the baselines range from 63.9% to 123.3% with a mean of about 83.6% for the five graph and composition based baselines; MSCA-EMNet reaches about 58% of that mean, because channel level affine modulation corrects the representation outside the training distribution and the Onsager and magnetoelectric reciprocity constraints suppress symmetry violations when samples are sparse.

Cross system transfer freezes the encoders and the BS-CrossAttn backbone and fine tunes only the FiLM channel and the prediction head. The transfer sets draw mainly on existing material, since Sn-Pb and In-Sn are long established solder systems: the Sn-Pb set holds 180 records, comprising 60 first principles configurations, 80 published measurements and 40 in-house experiments, and the In-Sn set 150 records comprising 50, 60 and 40, screened by the criteria applied to In-Pb and matched in processing range. Fine tuning runs 60 epochs at 3 × 10^−4^ with batch size 32, reported over five fold cross validation. MSCA-EMNet reaches 0.887 ± 0.012 for Sn-Pb and 0.856 ± 0.015 for In-Sn, whereas CGCNN and MEGNet under the same frozen setting fall to 0.52 to 0.60, as shown in Table 9. The gap is informative: Sn-Pb shares the Pb sublattice and hence the dominant SOC channel, whereas In-Sn contains no heavy element and forces the frozen atomic encoder outside its trained range. Subsets of the Sn-Pb data give 0.62 at 20 records, 0.75 at 50, 0.86 at 100 and 0.88 at 150, so about 100 records suffice to exceed 0.85; both curves are in Supplementary Table S6. This verifies the transferability provided by binding physical hierarchies while decoupling the processing channel.

**TABLE 9:** Datasets and results of cross system transfer with a frozen backbone.

Target system	First principles records	Published records	In-house records	Total	Fine tuned modules	MSCA-EMNet $R^{2}$	CGCNN $R^{2}$	MEGNet $R^{2}$
Sn-Pb	60	80	40	180	FiLM generator and prediction head	0.887 ± 0.012	0.58 ± 0.03	0.60 ± 0.03
In-Sn	50	60	40	150	FiLM generator and prediction head	0.856 ± 0.015	0.52 ± 0.04	0.55 ± 0.03

### 5.4 Research limitations and future work

Several limitations remain. The 800 DFT records derive from static atomic configurations and do not cover *in situ* defect evolution during hot pressing, and the independent experiments use conventional pressure equipment and do not reach ultrahigh pressure, so prediction confidence under extreme combinations is reduced. A further consequence of the scale coverage of Section 4.1 is that the process dependent part of the FiLM modulation is learned from the 200 training records that carry a hot pressing history, since the first principles records enter through the absent process embedding, so the effective sample size of the process channel is smaller than the nominal dataset size. Only two first order symmetries, Onsager reciprocity and magnetoelectric reciprocity, are embedded; higher order relations such as the Wiedemann-Franz law and the fluctuation dissipation theorem remain unconstrained and should be added once the corresponding response channels are available.

Future work follows two routes. The first introduces *in situ* STEM and *in situ* electromagnetic characterization to collect time series during hot pressing, extending the framework into a temporal predictor. The second integrates BS-CrossAttn with an equivariant tensor graph network so that spatial symmetry compresses the search space as a hard constraint. Both routes also provide a transferable basis for other strong SOC systems such as Ga-Sn-Bi and Sb-Bi-In.

## 6 Conclusion

To address the absence of a physically consistent framework supporting inverse process design for the electromagnetic coupling of hot pressed low dimensional In-Pb heterostructures, this study proposes and validates MSCA-EMNet.

1. The hierarchical encoder embeds atomic SOC, interfacial proximity effects, mesoscale texture and macroscopic processing conditions into independent nonoverlapping channels, replacing the degeneration of multiscale into multiresolution representation and making the input semantics auditable layer by layer.
2. BS-CrossAttn places the Query at scale k and the Key and Value at scale k±1 , combining an upward aggregation branch with a downward constraint feedback branch so that macroscopic conditions revise atomic layer embeddings through the composition of adjacent scale attention maps, overcoming the information flow limits of unidirectional hierarchical networks.
3. FiLM maps T , P , t , $D_{50}$ and the applied magnetic field to channel level affine parameters, reducing the overall nMAE to 0.041 and the extrapolation degradation rate from the graph based baseline mean of 83.6% to 48.8%. A worked case in Section 4.5 maps a nine component target back to a window of 168°C–174 °C, 62–70 MPa, 25–35 min and 8–12 μm, and six confirmatory hot pressings reproduce it within 7.6% on every component.
4. The joint prediction head and the Onsager and magnetoelectric reciprocity soft constraints regress electrical, magnetic and interfacial magnetoelectric responses as a whole, reducing the proportion of samples violating the Onsager relation from the baseline range of 12%–41% to 1.7%.
5. A scale availability mask and a component level label mask let first principles and hot pressed records of unequal scale coverage train within one model, the processing channel being driven by a learnable absent process embedding where no hot pressing history exists. The first principles branch improves the overall nMAE from 0.058 to 0.041 relative to training on the hot pressing records alone, and the result degrades smoothly under perturbation of the transferred descriptors, establishing the cross scale coupling as a property of the model rather than a consequence of how the absent block is handled.

Two limitations remain. The data distribution does not fully cover extreme processing combinations, and higher order constraints based on the Wiedemann-Franz law and fluctuation dissipation relations are not yet incorporated. The magnetoelectric channel treated here is the field induced interfacial response of a textured polycrystal, and a spontaneous bulk linear magnetoelectric effect remains outside the present scope. Future work will integrate *in situ* characterization data and equivariant hard constraints and extend the framework to adjacent systems such as Ga-Sn-Bi and Sb-Bi-In.

## Data Availability

The original contributions presented in the study are included in the article/Supplementary Material, further inquiries can be directed to the corresponding author.

## References

[B1] Babatain W. Kim M. S. Hussain M. M. (2024). From droplets to devices: recent advances in liquid metal droplet enabled electronics. Adv. Funct. Mater. 34 (31), 2308116. 10.1002/adfm.202308116

[B2] Batzner S. Musaelian A. Sun L. Geiger M. Mailoa J. P. Kornbluth M. (2022). E(3)-equivariant graph neural networks for data-efficient and accurate interatomic potentials. Nat. Commun. 13 (1), 2453. 10.1038/s41467-022-29939-5 PMC9068614 35508450

[B3] Bihlmayer G. Noël P. Vyalikh D. V. Chulkov E. V. Manchon A. (2022). Rashba-like physics in condensed matter. Nat. Rev. Phys. 4 (10), 642–659. 10.1038/s42254-022-00490-y

[B4] Blöchl P. E. (1994). Projector augmented-wave method. Phys. Rev. B 50 (24), 17953–17979. 10.1103/physrevb.50.17953 9976227

[B5] Bobkov G. A. Bokai K. A. Otrokov M. M. Bobkov A. M. Bobkova I. V. (2024). Gate-controlled proximity effect in superconductor/ferromagnet van der Waals heterostructures. Phys. Rev. Mater. 8 (10), 104801. 10.1103/physrevmaterials.8.104801

[B6] Chen C. Ong S. P. (2022). A universal graph deep learning interatomic potential for the periodic table. Nat. Comput. Sci. 2 (11), 718–728. 10.1038/s43588-022-00349-3 38177366

[B7] Choudhary K. DeCost B. (2021). Atomistic line graph neural network for improved materials property predictions. Npj Comput. Mater. 7 (1), 185. 10.1038/s41524-021-00650-1

[B8] Deng B. Zhong P. Jun K. Riebesell J. Han K. Bartel C. J. (2023). CHGNet as a pretrained universal neural network potential for charge-informed atomistic modelling. Nat. Mach. Intell. 5 (9), 1031–1041. 10.1038/s42256-023-00716-3

[B9] Dong L. Zhang X. Yang Z. Shen L. Lu Y. (2025). Accurate piezoelectric tensor prediction with equivariant attention tensor graph neural network. Npj Comput. Mater. 11 (1), 63. 10.1038/s41524-025-01546-0

[B10] Droghetti A. Tokatly I. V. (2023). Current-induced spin polarization at metallic surfaces from first principles. Phys. Rev. B 107 (17), 174433. 10.1103/physrevb.107.174433

[B11] Flokstra M. Stewart R. Yim C. M. Trainer C. Wahl P. Miller D. (2023). Spin-orbit driven superconducting proximity effects in Pt/Nb thin films. Nat. Commun. 14 (1), 5081. 10.1038/s41467-023-40757-1 37604804 PMC10442328

[B12] Geng W. R. Tang Y. L. Zhu Y. L. Wang Y. Wu B. Yang L. (2022). Magneto-electric-optical coupling in multiferroic BiFeO_3_-based films. Adv. Mater. 34 (29), 2106396. 10.1002/adma.202106396 35730916

[B13] Guo X. Li X. Zhu Z. Behnia K. (2023). Onsager reciprocal relation between anomalous transverse coefficients of an anisotropic antiferromagnet. Phys. Rev. Lett. 131 (24), 246302. 10.1103/physrevlett.131.246302 38181139

[B14] Hillaire K. Elassy K. S. Dickey M. D. Nadimi S. R. Kiani A. (2024). Interfacial tension hysteresis of eutectic gallium-indium. Adv. Funct. Mater. 34 (30), 2311501. 10.1002/adfm.202311501

[B15] Ianovskaia A. S. Bobkov G. A. Bobkov A. M. Bobkova I. V. (2024). Magnetic proximity effect in superconductor/ferromagnet van der Waals heterostructures: dependence on the number of superconducting monolayers. Phys. Rev. B 110 (21), 214503. 10.1103/physrevb.110.214503

[B16] Kang Y. Park H. Smit B. Kim J. (2023). A multi-modal pre-training transformer for universal transfer learning in metal-organic frameworks. Nat. Mach. Intell. 5 (3), 309–318. 10.1038/s42256-023-00628-2

[B17] Kim S. Lee H. W. Choi G. M. (2023). Giant spin-orbit torque in sputter-deposited Bi films. Adv. Sci. 10 (31), 2303831. 10.1002/advs.202303831 PMC10625106 37679062

[B18] Kresse G. Furthmüller J. (1996). Efficiency of *ab-initio* total energy calculations for metals and semiconductors using a plane-wave basis set. Comput. Mater. Sci. 6 (1), 15–50. 10.1016/0927-0256(96)00008-0

[B19] Ma Q. Ai L. Zhang Y. Xiu F. (2025). Hybrid superconducting-magnetic van der Waals heterostructures: physics and application. Adv. Mater. 37, 2507866. 10.1002/adma.202507866 40922711 PMC13549256

[B20] Madani M. Lacivita V. Shin Y. Tarakanova A. (2025). Accelerating materials property prediction via a hybrid transformer graph framework that leverages four body interactions. Npj Comput. Mater. 11 (1), 15. 10.1038/s41524-024-01472-7 39839990 PMC11742729

[B21] Mao Z. Li W. Tan J. (2024). Dielectric tensor prediction for inorganic materials using latent information from preferred potential. Npj Comput. Mater. 10 (1), 265. 10.1038/s41524-024-01450-z

[B22] Massa D. Cieśliński D. Naghdi A. Papanikolaou S. (2024). Substitutional alloying using crystal graph neural networks. AIP Adv. 14 (1), 015023. 10.1063/5.0163765

[B23] Merchant A. Batzner S. Schoenholz S. S. Aykol M. Cheon G. Cubuk E. D. (2023). Scaling deep learning for materials discovery. Nature 624 (7990), 80–85. 10.1038/s41586-023-06735-9 38030720 PMC10700131

[B24] Musaelian A. Batzner S. Johansson A. Sun L. Owen C. J. Kornbluth M. (2023). Learning local equivariant representations for large scale atomistic dynamics. Nat. Commun. 14 (1), 579. 10.1038/s41467-023-36329-y 36737620 PMC9898554

[B25] Perdew J. P. Burke K. Ernzerhof M. (1996). Generalized gradient approximation made simple. Phys. Rev. Lett. 77 (18), 3865–3868. 10.1103/PhysRevLett.77.3865 10062328

[B26] Razavi S. Jakeman A. Saltelli A. Prieur C. Iooss B. Borgonovo E. (2021). The future of sensitivity analysis: an essential discipline for systems modeling and policy support. Environ. Model. Softw. 137, 104954. 10.1016/j.envsoft.2020.104954

[B27] Reiser P. Neubert M. Eberhard A. Torresi L. Zhou C. Shao C. (2022). Graph neural networks for materials science and chemistry. Commun. Mater. 3 (1), 93. 10.1038/s43246-022-00315-6 36468086 PMC9702700

[B28] Schmidt J. Pettersson L. Verdozzi C. Botti S. Marques M. A. L. (2021). Crystal graph attention networks for the prediction of stable materials. Sci. Adv. 7 (49), eabi7948. 10.1126/sciadv.abi7948 34860548 PMC8641929

[B29] Semenov A. S. Trapp J. Nöthe M. Kieback B. Wallmersperger T. (2023). Multiphysics simulation and experimental investigation of the densification of metals by spark plasma sintering. Adv. Eng. Mater. 25 (21), 2300764. 10.1002/adem.202300764

[B30] Shwartz-Ziv R. Armon A. (2022). Tabular data: deep learning is not all you need. Inf. Fusion 81, 84–90. 10.1016/j.inffus.2021.11.011

[B31] Sui F. Jin M. Zhang Y. Qi R. Wu Y. N. Huang R. (2023). Sliding ferroelectricity in van der Waals layered γ-InSe semiconductor. Nat. Commun. 14 (1), 36. 10.1038/s41467-022-35490-0 36596789 PMC9810696

[B32] Wang A. Y. T. Kauwe S. K. Murdock R. J. Sparks T. D. (2021). Compositionally restricted attention-based network for materials property predictions. Npj Comput. Mater. 7 (1), 77. 10.1038/s41524-021-00545-1

[B33] Wang Y. Wang P. Wang H. Xu B. Li H. Cheng M. (2023). Room-temperature magnetoelectric coupling in atomically thin ε-Fe_2_O_3_ . Adv. Mater. 35 (11), 2209465. 10.1002/adma.202209465 36460029

[B34] Xu X. Yang L. (2024). Enhanced magnetoelectric coupling in two-dimensional hybrid multiferroic heterostructures. Phys. Rev. B 110 (16), 165408. 10.1103/physrevb.110.165408

[B35] Xu Y. Deng W. Zheng J. (2024). Study of crystal property prediction based on dual attention mechanism and transfer learning. AIP Adv. 14 (11), 115001. 10.1063/5.0232308

[B36] Yi M. Wang W. Xue M. Gong Q. Xu B. X. (2023). Modeling and simulation of sintering process across scales. Archives Comput. Methods Eng. 30 (5), 3325–3358. 10.1007/s11831-023-09905-0

[B37] Yoshizawa S. Kim H. Kawakami T. Yaji K. Yokota K. Komori F. (2021). Atomic-layer Rashba type superconductor protected by dynamic spin momentum locking. Nat. Commun. 12 (1), 1462. 10.1038/s41467-021-21642-1 33674608 PMC7935850

[B38] Zhang Y. Chen P. Li J. Li B. Yuan Y. Liu Z. (2023). Recent advances in engineering of 2D materials-based heterostructures for electrochemical energy conversion. Adv. Sci. 10 (31), 2302301. 10.1002/advs.202302301 PMC10625098 37743245

[B39] Zhao Z. Zhang X. Zhang H. Tang H. Liang Y. (2022). Numerical investigation into pressure-assisted sintering using fully coupled mechano-diffusional phase field model. Int. J. Solids Struct. 234-235, 111253. 10.1016/j.ijsolstr.2021.111253

[B40] Zhong Y. Liu S. Zhang B. Tao Z. Sun Y. Chu W. (2024). Accelerating the calculation of electron-phonon coupling strength with machine learning. Nat. Comput. Sci. 4 (8), 615–625. 10.1038/s43588-024-00668-7 39117916

[B41] Zollner K. Kurpas M. Gmitra M. Fabian J. (2025). First principles determination of spin-orbit coupling parameters in two-dimensional materials. Nat. Rev. Phys. 7 (5), 255–268. 10.1038/s42254-025-00818-4

