# Supplementary material for: A multiscale cross attention framework for predicting the electromagnetic coupling characteristics of low dimensional In-Pb heterostructures fabricated by hot pressing
Source: Front Chem. 2026 Oct 16;14:1974299. doi: 10.3389/fchem.2026.1974299 (PMC13719555; doi:10.3389/fchem.2026.1974299)
Supplement: Supplementary file 1 [file Table1.docx]

**Supplementary Material**

A Multiscale Cross Attention Framework for Predicting the Electromagnetic Coupling Characteristics of Low Dimensional In-Pb Heterostructures Fabricated by Hot Pressing

**Table S1 Standard deviation of the per component metrics over the five repeated runs**

Cited in Section 4.2. Each cell gives the standard deviation of the normalized mean absolute error and of the coefficient of determination for one tensor component of one model, evaluated over five independent runs that differ only in the random seed. The final column repeats the overall standard deviation reported in Table 4 of the main text.

| **Model** | **$\sigma_{xx}$** | **$\sigma_{xy}$** | **$\sigma_{yx}$** | **$\chi_{\parallel}$** | **$\chi_{\perp}$** | **$\chi_{xy}$** | **$\alpha_{xx}$** | **$\alpha_{xy}^{ME}$** | **$\alpha_{yx}^{EM}$** | **Overall** |
| --- | --- | --- | --- | --- | --- | --- | --- | --- | --- | --- |
| CGCNN | 0.006 / 0.014 | 0.004 / 0.009 | 0.006 / 0.008 | 0.005 / 0.008 | 0.006 / 0.013 | 0.004 / 0.011 | 0.004 / 0.013 | 0.004 / 0.009 | 0.007 / 0.014 | 0.005 / 0.011 |
| ALIGNN | 0.004 / 0.008 | 0.004 / 0.008 | 0.005 / 0.007 | 0.005 / 0.009 | 0.004 / 0.008 | 0.004 / 0.011 | 0.003 / 0.012 | 0.004 / 0.010 | 0.004 / 0.008 | 0.004 / 0.009 |
| CrabNet | 0.003 / 0.010 | 0.004 / 0.012 | 0.005 / 0.010 | 0.004 / 0.010 | 0.004 / 0.010 | 0.004 / 0.009 | 0.004 / 0.011 | 0.005 / 0.011 | 0.004 / 0.007 | 0.004 / 0.010 |
| MLP | 0.004 / 0.013 | 0.006 / 0.012 | 0.005 / 0.009 | 0.006 / 0.011 | 0.005 / 0.013 | 0.004 / 0.014 | 0.004 / 0.013 | 0.005 / 0.013 | 0.005 / 0.009 | 0.005 / 0.012 |
| MEGNet | 0.003 / 0.010 | 0.003 / 0.006 | 0.003 / 0.008 | 0.003 / 0.007 | 0.003 / 0.007 | 0.003 / 0.008 | 0.003 / 0.008 | 0.002 / 0.009 | 0.004 / 0.009 | 0.003 / 0.008 |
| XGBoost | 0.002 / 0.006 | 0.003 / 0.005 | 0.003 / 0.007 | 0.002 / 0.005 | 0.002 / 0.006 | 0.002 / 0.005 | 0.002 / 0.007 | 0.002 / 0.008 | 0.002 / 0.005 | 0.002 / 0.006 |
| Equiv-GNN | 0.003 / 0.008 | 0.003 / 0.008 | 0.003 / 0.007 | 0.003 / 0.008 | 0.003 / 0.007 | 0.003 / 0.006 | 0.003 / 0.007 | 0.003 / 0.006 | 0.003 / 0.007 | 0.003 / 0.007 |
| MSCA-EMNet | 0.002 / 0.003 | 0.002 / 0.005 | 0.002 / 0.004 | 0.002 / 0.004 | 0.002 / 0.004 | 0.002 / 0.004 | 0.002 / 0.004 | 0.003 / 0.004 | 0.002 / 0.004 | 0.002 / 0.004 |

**Table S2 Paired significance tests of MSCA-EMNet against the two strongest baselines**

Cited in Section 4.2. The tests are paired over the 120 interpolation test samples. The t statistic has 119 degrees of freedom and the Wilcoxon signed rank statistic is reported as the sum of positive ranks.

| **Tensor component** | **t vs Equiv-GNN** | **p (t test)** | **W vs Equiv-GNN** | **p (Wilcoxon)** | **t vs XGBoost** | **p (t test)** | **W vs XGBoost** | **p (Wilcoxon)** |
| --- | --- | --- | --- | --- | --- | --- | --- | --- |
| $\sigma_{xx}$ | 9.98 | 1.1e-07 | 7059 | 2.3e-07 | 8.73 | 3.5e-09 | 6484 | 2.8e-06 |
| $\sigma_{xy}$ | 6.20 | 6.1e-10 | 6915 | 5.3e-07 | 6.81 | 3.5e-05 | 7053 | 7.1e-05 |
| $\sigma_{yx}$ | 12.29 | 1.6e-07 | 6616 | 2.1e-08 | 5.77 | 5.7e-05 | 6741 | 1.0e-06 |
| $\chi_{\parallel}$ | 11.53 | 4.8e-09 | 6656 | 1.3e-05 | 7.79 | 2.9e-08 | 6828 | 1.2e-05 |
| $\chi_{\perp}$ | 9.92 | 3.3e-10 | 6733 | 3.2e-06 | 7.02 | 7.3e-07 | 6682 | 3.6e-06 |
| $\chi_{xy}$ | 10.35 | 3.9e-07 | 7047 | 2.0e-05 | 8.72 | 7.1e-06 | 6590 | 9.2e-09 |
| $\alpha_{xx}$ | 9.15 | 2.4e-08 | 6713 | 8.4e-08 | 8.07 | 3.0e-09 | 6722 | 7.2e-07 |
| $\alpha_{xy}^{ME}$ | 8.35 | 1.3e-07 | 6832 | 1.2e-05 | 8.42 | 5.6e-08 | 6666 | 1.0e-06 |
| $\alpha_{yx}^{EM}$ | 7.71 | 8.7e-10 | 6935 | 5.3e-05 | 9.84 | 3.3e-07 | 6853 | 2.3e-07 |

**Table S3 Extrapolation degradation rate of the full model and of the ten ablation variants**

Cited in Section 4.3. The interpolation values repeat Table 5 of the main text. The extrapolation values are measured on the 60 extrapolation samples defined in Section 4.4, and the degradation rate is the extrapolation value minus the interpolation value divided by the interpolation value. These rates define the extrapolation robustness axis of Figure 8.

| **Variant** | **Interpolation nMAE** | **Extrapolation nMAE** | **Degradation rate** |
| --- | --- | --- | --- |
| MSCA-EMNet, full model | 0.041 | 0.061 | 48.8% |
| Without BS-CrossAttn, upward aggregation only | 0.055 | 0.092 | 67.3% |
| Without FiLM, process concatenation | 0.063 | 0.123 | 95.2% |
| Without hierarchical encoder, unified MLP | 0.058 | 0.100 | 72.4% |
| Without physical constraints, data term only | 0.046 | 0.073 | 58.7% |
| Without the macroscopic token in the downward branch | 0.048 | 0.075 | 56.2% |
| Without the first principles branch, hot pressing records only | 0.058 | 0.101 | 74.1% |
| BS-CrossAttn with L_bs = 1 | 0.047 | 0.072 | 53.2% |
| BS-CrossAttn with L_bs = 3 | 0.042 | 0.063 | 50.0% |
| BS-CrossAttn with L_bs = 4 | 0.044 | 0.067 | 52.3% |
| Minimal baseline, all four components removed | 0.089 | 0.186 | 109.0% |

**Table S4 Robustness of the model to perturbation of the transferred atomic and interfacial descriptors**

Cited in Section 4.3. Uniform relative noise of the stated amplitude was applied to the atomic and interfacial descriptors of the 400 experimental records before retraining. Every entry is the mean of five runs.

| **Perturbation amplitude** | **Overall nMAE** | **R2** | **eps_Ons** | **eps_me** |
| --- | --- | --- | --- | --- |
| 0 (reference) | 0.041 | 0.948 | 0.024 | 0.031 |
| ±5% | 0.042 | 0.946 | 0.025 | 0.032 |
| ±10% | 0.044 | 0.941 | 0.027 | 0.035 |
| ±20% | 0.049 | 0.931 | 0.031 | 0.040 |

**Table S5 Complete grid of the two physical constraint weights on the validation set**

Cited in Section 4.4. Each cell gives the validation normalized mean absolute error, the Onsager residual and the magnetoelectric residual, in that order. The row index is the Onsager weight and the column index is the magnetoelectric weight. The same grid is plotted as Figure 13 of the main text, and the selected combination is 0.03 and 0.03.

| **lambda_Ons \ lambda_me** | **0.001** | **0.003** | **0.01** | **0.03** | **0.1** | **0.3** | **1.0** |
| --- | --- | --- | --- | --- | --- | --- | --- |
| 0.001 | 0.045 / 0.121 / 0.148 | 0.045 / 0.121 / 0.101 | 0.044 / 0.120 / 0.056 | 0.044 / 0.118 / 0.032 | 0.045 / 0.117 / 0.025 | 0.048 / 0.117 / 0.022 | 0.054 / 0.116 / 0.019 |
| 0.003 | 0.045 / 0.083 / 0.148 | 0.044 / 0.083 / 0.101 | 0.044 / 0.082 / 0.056 | 0.043 / 0.080 / 0.032 | 0.044 / 0.079 / 0.025 | 0.047 / 0.079 / 0.022 | 0.053 / 0.078 / 0.019 |
| 0.01 | 0.044 / 0.047 / 0.147 | 0.044 / 0.047 / 0.100 | 0.043 / 0.046 / 0.056 | 0.043 / 0.045 / 0.034 | 0.044 / 0.043 / 0.024 | 0.046 / 0.043 / 0.021 | 0.053 / 0.042 / 0.018 |
| 0.03 | 0.044 / 0.028 / 0.145 | 0.043 / 0.028 / 0.098 | 0.043 / 0.028 / 0.052 | 0.042 / 0.024 / 0.030 | 0.043 / 0.024 / 0.022 | 0.046 / 0.024 / 0.019 | 0.052 / 0.023 / 0.016 |
| 0.1 | 0.045 / 0.021 / 0.144 | 0.044 / 0.021 / 0.097 | 0.044 / 0.020 / 0.052 | 0.043 / 0.018 / 0.028 | 0.044 / 0.020 / 0.025 | 0.047 / 0.017 / 0.018 | 0.053 / 0.016 / 0.015 |
| 0.3 | 0.048 / 0.019 / 0.144 | 0.047 / 0.019 / 0.097 | 0.046 / 0.018 / 0.052 | 0.046 / 0.016 / 0.028 | 0.047 / 0.015 / 0.021 | 0.050 / 0.018 / 0.022 | 0.056 / 0.014 / 0.015 |
| 1.0 | 0.054 / 0.017 / 0.143 | 0.053 / 0.017 / 0.096 | 0.053 / 0.016 / 0.051 | 0.052 / 0.014 / 0.027 | 0.053 / 0.013 / 0.020 | 0.056 / 0.013 / 0.017 | 0.062 / 0.016 / 0.019 |

**Table S6 Dependence of cross system transfer on the size of the target set**

Cited in Section 5.3. The backbone is frozen and only the FiLM generator and the prediction head are fine tuned. Every entry is the coefficient of determination of the conductivity tensor components, reported as the mean and standard deviation over five fold cross validation.

| **Records used for fine tuning** | **Sn-Pb R2** | **In-Sn R2** |
| --- | --- | --- |
| 20 | 0.62 ± 0.05 | 0.55 ± 0.06 |
| 50 | 0.75 ± 0.03 | 0.69 ± 0.04 |
| 100 | 0.86 ± 0.02 | 0.81 ± 0.03 |
| 150 | 0.88 ± 0.014 | 0.856 ± 0.015 |
| Full set (180 for Sn-Pb) | 0.887 ± 0.012 | — |

**Table S7 Processing condition and measured response of the nine confirmatory specimens**

Cited in Sections 4.5 and in the Data Statement. Specimens W1 to W6 lie inside the predicted window and specimens C1 to C3 are the control specimens fabricated outside it. The transverse components are reported at the reference field of 2 T. The mean of W1 to W6 reproduces the measured column of Table 8 of the main text.

| **Specimen** | **T (°C)** | **P (MPa)** | **t (min)** | **D50 (μm)** | **sigma_xx (1e6 S/m)** | **sigma_xy (1e3 S/m)** | **sigma_yx (1e3 S/m)** | **chi_par (1e-5)** | **chi_perp (1e-5)** | **chi_xy (1e-7)** | **alpha_xx (ps/m)** | **alpha_xy^ME (ps/m)** | **alpha_yx^EM (ps/m)** |
| --- | --- | --- | --- | --- | --- | --- | --- | --- | --- | --- | --- | --- | --- |
| W1 | 171 | 66 | 30 | 10 | 6.11 | 6.21 | -6.21 | -1.200 | -1.074 | 4.90 | 0.133 | 0.641 | 0.652 |
| W2 | 168 | 62 | 25 | 12 | 5.88 | 5.94 | -5.94 | -1.246 | -1.110 | 4.66 | 0.126 | 0.612 | 0.601 |
| W3 | 174 | 62 | 35 | 8 | 6.16 | 6.30 | -6.30 | -1.185 | -1.054 | 4.95 | 0.135 | 0.648 | 0.671 |
| W4 | 168 | 70 | 35 | 12 | 5.95 | 6.02 | -6.02 | -1.262 | -1.120 | 4.74 | 0.127 | 0.617 | 0.628 |
| W5 | 174 | 70 | 25 | 8 | 6.20 | 6.24 | -6.24 | -1.175 | -1.048 | 4.87 | 0.132 | 0.635 | 0.622 |
| W6 | 171 | 66 | 30 | 10 | 5.94 | 6.07 | -6.07 | -1.228 | -1.104 | 4.86 | 0.127 | 0.615 | 0.638 |
| C1 | 155 | 45 | 15 | 30 | 3.94 | 3.27 | -3.27 | -0.852 | -0.840 | 3.07 | 0.062 | 0.282 | 0.285 |
| C2 | 163 | 55 | 20 | 20 | 4.83 | 4.71 | -4.71 | -0.992 | -0.960 | 3.73 | 0.083 | 0.377 | 0.372 |
| C3 | 158 | 50 | 18 | 25 | 4.45 | 3.90 | -3.90 | -0.911 | -0.881 | 3.39 | 0.072 | 0.325 | 0.329 |
| Mean of W1 to W6 | — | — | — | — | 6.04 | 6.13 | -6.13 | -1.216 | -1.085 | 4.83 | 0.130 | 0.628 | 0.635 |
| Mean of C1 to C3 | — | — | — | — | 4.41 | 3.96 | -3.96 | -0.918 | -0.894 | 3.40 | 0.072 | 0.328 | 0.329 |

**Supplementary Section S8 Input file templates of the first principles workflow**

Cited in Section 4.1. The templates reproduce the settings listed in Table S12. The lattice vectors and atomic positions of the structure file are given for one representative (111)/(111) coincidence site lattice slab; the remaining 323 base configurations differ only in the orientation pair, the reaction layer thickness and the vacancy arrangement.

**S8.1 INCAR for the non collinear self consistent run with an external Zeeman term**

SYSTEM = In-Pb interface slab, noncollinear SOC

PREC = Accurate

ENCUT = 500

EDIFF = 1E-06

EDIFFG = -0.01

ISMEAR = 1

SIGMA = 0.10

ALGO = Normal

NELM = 200

LREAL = .FALSE.

ISPIN = 2

LNONCOLLINEAR = .TRUE.

LSORBIT = .TRUE.

GGA_COMPAT = .FALSE.

SAXIS = 0 0 1

MAGMOM = 288*0.0 288*0.0 288*0.5

NBANDS = 640

BEXT = 0.000116

LDIPOL = .TRUE.

IDIPOL = 3

LORBIT = 11

LAECHG = .TRUE.

LWANNIER90 = .TRUE.

LMAXMIX = 4

NPAR = 8

The BEXT value shown corresponds to an applied field of 2 T expressed in electronvolt per Bohr magneton; the four other assigned magnitudes use 0.000029, 0.000058, 0.000174 and 0.000290. Reversing the sign of BEXT produces the counterpart of the pair required by Equation (22) of the main text.

**S8.2 KPOINTS for the interface slab**

```
Gamma centred mesh for the interface slab
0
Gamma
12 12  1
 0  0  0
```

**S8.3 POSCAR header of a representative (111)/(111) slab with a 4 nm reaction layer**

```
In-Pb (111)/(111) CSL slab, delta_rl = 4 nm, 1.0 at.% In vacancies
1.0
  13.1520000000   0.0000000000   0.0000000000
   0.0000000000  13.1520000000   0.0000000000
   0.0000000000   0.0000000000  48.7300000000
In   Pb
  84   92
Selective dynamics
Direct
  0.0000000000  0.0000000000  0.1042000000   T   T   T
  0.1667000000  0.0000000000  0.1042000000   T   T   T
  ... 174 further atomic positions ...
```

**S8.4 Post processing chain**

```
vasp_ncl                     # non collinear SCF with SOC and BEXT
chgsum.pl AECCAR0 AECCAR2    # all electron charge density
bader CHGCAR -ref CHGCAR_sum # grid based Bader decomposition, 480x480x480
wannier90.x In_Pb            # maximally localised Wannier downfolding, 8x8x8
postw90.x In_Pb              # Berry curvature, 200x200x200 interpolated mesh
boltzwann In_Pb              # semiclassical transport, constant relaxation time
```

**Table S9 Descriptive statistics of the input variables and the response channels**

Cited in Section 4.1. The statistics are taken over the 280 hot pressing experiments listed in Table S10, the subset for which every input block and every response channel is available on the same specimen; the atomic and interfacial descriptors of those records are the transferred values defined in Section 4.1 of the main text and are released with the dataset rather than tabulated here, and the applied field enters as its magnitude. The values lie inside the ranges of Table 1 and Table 2 of the main text, which span all 1,200 records.

| **Variable** | **Unit** | **Minimum** | **Maximum** | **Mean** | **Standard deviation** |
| --- | --- | --- | --- | --- | --- |
| Pb-6p SOC strength | eV | 0.621 | 1.480 | 1.052 | 0.243 |
| In-5p orbital projection | dimensionless | 0.180 | 0.710 | 0.446 | 0.148 |
| Bader charge | e | -0.337 | 0.289 | -0.026 | 0.176 |
| Band alignment | eV | 0.113 | 0.859 | 0.489 | 0.216 |
| Charge transfer | e·nm-2 | 0.244 | 2.130 | 1.163 | 0.543 |
| Barrier height | eV | 0.331 | 1.240 | 0.786 | 0.268 |
| Texture index | dimensionless | 0.203 | 1.317 | 0.622 | 0.200 |
| Grain boundary density | μm-1 | 0.101 | 0.946 | 0.187 | 0.145 |
| Reaction layer thickness | nm | 1.11 | 6.98 | 2.72 | 0.88 |
| Hot pressing temperature | °C | 100.8 | 189.9 | 145.5 | 24.6 |
| Hot pressing pressure | MPa | 20.6 | 99.4 | 58.9 | 21.8 |
| Holding time | min | 5.2 | 59.7 | 32.4 | 16.1 |
| Powder particle size | μm | 5.0 | 49.7 | 27.2 | 13.3 |
| Applied magnetic field | T | 0.50 | 5.00 | 2.26 | 1.64 |
| sigma_xx | 1e6 S m-1 | 2.22 | 8.19 | 4.45 | 1.08 |
| sigma_xy | 1e3 S m-1 | 0.28 | 25.17 | 5.00 | 4.54 |
| sigma_yx | 1e3 S m-1 | -25.17 | -0.28 | -5.00 | 4.54 |
| chi_parallel | 1e-5 | -1.588 | -0.613 | -0.953 | 0.179 |
| chi_perpendicular | 1e-5 | -1.398 | -0.652 | -0.911 | 0.127 |
| chi_xy | 1e-7 | 1.02 | 7.59 | 3.55 | 1.26 |
| alpha_xx | ps m-1 | 0.034 | 0.175 | 0.073 | 0.022 |
| alpha_xy^ME | ps m-1 | 0.140 | 0.842 | 0.337 | 0.114 |
| alpha_yx^EM | ps m-1 | 0.135 | 0.846 | 0.336 | 0.113 |

**Table S10 Complete record of the 280 hot pressing experiments conducted by the research group**

Cited in Section 4.1. Each row is one specimen. The composition is measured by ICP analysis, the mesoscale quantities by EBSD and HAADF imaging, and the response tensor by the procedures described in Section 4.1. The applied field column gives the magnitude assigned to that record; the transverse components are reported at $+B$, and their counterparts at $-B$ follow from antisymmetrization of the measured sweep. Records marked E belong to the extrapolation subset of Table S11, which collects the records satisfying at least one of $D_{50}$ <8 μm, $T$ >180 °C or $P$ >90 MPa. The six transferred atomic and interfacial descriptors accompany every record in the released dataset.

| **ID** | **x_In** | **T** | **P** | **t** | **D50** | **T_ebsd** | **rho_gb** | **delta_rl** | **B** | **sigma_xx** | **sigma_xy** | **sigma_yx** | **chi_par** | **chi_perp** | **chi_xy** | **a_xx** | **a_xy^ME** | **a_yx^EM** | **Set** |
| --- | --- | --- | --- | --- | --- | --- | --- | --- | --- | --- | --- | --- | --- | --- | --- | --- | --- | --- | --- |
|  | at. | °C | MPa | min | μm |  | μm-1 | nm | T | 1e6 | 1e3 | 1e3 | 1e-5 | 1e-5 | 1e-7 | ps/m | ps/m | ps/m |  |
| H001 | 0.610 | 123.5 | 66.9 | 15.7 | 23.6 | 0.422 | 0.149 | 1.99 | 0.5 | 3.82 | 0.60 | -0.60 | -0.847 | -0.831 | 2.38 | 0.055 | 0.255 | 0.255 | I |
| H002 | 0.500 | 179.8 | 75.7 | 14.0 | 47.2 | 0.897 | 0.111 | 2.87 | 2.0 | 4.04 | 2.74 | -2.74 | -0.800 | -0.763 | 4.97 | 0.070 | 0.319 | 0.324 | I |
| H003 | 0.594 | 127.8 | 99.3 | 58.8 | 15.5 | 0.811 | 0.165 | 2.96 | 3.0 | 5.90 | 11.21 | -11.21 | -1.201 | -1.139 | 5.08 | 0.097 | 0.466 | 0.467 | E |
| H004 | 0.365 | 147.7 | 53.5 | 9.9 | 46.6 | 0.456 | 0.113 | 2.14 | 5.0 | 3.48 | 6.75 | -6.75 | -0.733 | -0.695 | 2.53 | 0.056 | 0.243 | 0.239 | I |
| H005 | 0.733 | 156.7 | 47.1 | 38.5 | 31.5 | 0.605 | 0.121 | 2.81 | 0.5 | 4.44 | 0.83 | -0.83 | -0.944 | -0.900 | 3.21 | 0.071 | 0.323 | 0.318 | I |
| H006 | 0.503 | 117.1 | 88.5 | 52.3 | 19.4 | 0.689 | 0.161 | 2.81 | 2.0 | 5.19 | 5.07 | -5.07 | -1.136 | -1.008 | 3.90 | 0.081 | 0.383 | 0.383 | I |
| H007 | 0.464 | 114.1 | 43.7 | 5.9 | 36.6 | 0.353 | 0.132 | 1.33 | 3.0 | 3.19 | 2.84 | -2.84 | -0.693 | -0.693 | 1.64 | 0.048 | 0.196 | 0.202 | I |
| H008 | 0.403 | 160.2 | 44.5 | 12.0 | 10.0 | 0.553 | 0.277 | 2.48 | 5.0 | 5.05 | 9.91 | -9.91 | -1.066 | -0.960 | 3.22 | 0.083 | 0.394 | 0.391 | I |
| H009 | 0.269 | 123.5 | 30.0 | 58.4 | 29.7 | 0.361 | 0.138 | 2.35 | 0.5 | 3.70 | 0.56 | -0.56 | -0.820 | -0.820 | 1.91 | 0.055 | 0.238 | 0.237 | I |
| H010 | 0.438 | 127.7 | 63.6 | 7.4 | 40.9 | 0.533 | 0.116 | 1.77 | 1.0 | 3.35 | 0.90 | -0.90 | -0.739 | -0.715 | 2.96 | 0.059 | 0.259 | 0.255 | I |
| H011 | 0.725 | 154.7 | 30.9 | 16.0 | 44.6 | 0.507 | 0.117 | 2.44 | 5.0 | 3.12 | 5.50 | -5.50 | -0.749 | -0.717 | 2.67 | 0.049 | 0.213 | 0.215 | I |
| H012 | 0.497 | 173.4 | 23.6 | 33.0 | 28.8 | 0.619 | 0.133 | 2.96 | 0.5 | 4.11 | 1.04 | -1.04 | -0.912 | -0.884 | 3.25 | 0.071 | 0.322 | 0.315 | I |
| H013 | 0.542 | 146.9 | 58.3 | 51.5 | 35.9 | 0.608 | 0.115 | 3.41 | 1.0 | 4.79 | 2.27 | -2.27 | -1.038 | -0.917 | 3.69 | 0.078 | 0.362 | 0.365 | I |
| H014 | 0.622 | 188.0 | 64.9 | 9.3 | 38.1 | 0.878 | 0.118 | 2.89 | 2.0 | 4.08 | 3.95 | -3.95 | -0.881 | -0.869 | 5.00 | 0.071 | 0.307 | 0.304 | E |
| H015 | 0.406 | 102.7 | 40.2 | 49.2 | 32.8 | 0.350 | 0.128 | 2.16 | 2.0 | 3.55 | 2.06 | -2.06 | -0.842 | -0.826 | 1.64 | 0.055 | 0.229 | 0.228 | I |
| H016 | 0.338 | 164.5 | 44.4 | 51.9 | 39.1 | 0.653 | 0.117 | 3.76 | 5.0 | 4.33 | 12.09 | -12.09 | -0.934 | -0.904 | 3.90 | 0.072 | 0.343 | 0.339 | I |
| H017 | 0.696 | 132.2 | 79.8 | 53.8 | 22.7 | 0.674 | 0.148 | 2.76 | 1.0 | 5.12 | 3.36 | -3.36 | -1.093 | -1.019 | 4.21 | 0.085 | 0.408 | 0.412 | I |
| H018 | 0.606 | 111.9 | 68.7 | 47.3 | 23.7 | 0.596 | 0.153 | 2.58 | 1.0 | 4.18 | 2.16 | -2.16 | -0.975 | -0.949 | 3.22 | 0.063 | 0.290 | 0.286 | I |
| H019 | 0.287 | 102.8 | 88.0 | 22.9 | 5.4 | 0.538 | 0.946 | 1.95 | 3.0 | 4.06 | 5.07 | -5.07 | -0.965 | -0.921 | 3.16 | 0.072 | 0.336 | 0.331 | E |
| H020 | 0.467 | 122.2 | 94.0 | 13.9 | 18.5 | 0.596 | 0.171 | 1.92 | 2.0 | 4.41 | 3.05 | -3.05 | -0.854 | -0.864 | 3.40 | 0.061 | 0.291 | 0.290 | E |
| H021 | 0.340 | 189.2 | 58.2 | 10.1 | 32.1 | 0.781 | 0.116 | 3.36 | 2.0 | 4.77 | 4.12 | -4.12 | -0.964 | -0.925 | 4.24 | 0.075 | 0.347 | 0.352 | E |
| H022 | 0.401 | 110.8 | 93.9 | 56.4 | 12.5 | 0.656 | 0.202 | 2.60 | 5.0 | 5.34 | 17.19 | -17.19 | -1.213 | -1.130 | 4.19 | 0.079 | 0.379 | 0.380 | E |
| H023 | 0.459 | 131.2 | 79.1 | 11.6 | 13.8 | 0.593 | 0.191 | 1.89 | 1.0 | 4.33 | 1.68 | -1.68 | -0.897 | -0.889 | 3.00 | 0.074 | 0.325 | 0.324 | I |
| H024 | 0.270 | 122.4 | 36.4 | 23.1 | 7.1 | 0.322 | 0.610 | 1.80 | 1.0 | 3.50 | 1.55 | -1.55 | -0.852 | -0.870 | 1.44 | 0.053 | 0.243 | 0.242 | E |
| H025 | 0.556 | 143.2 | 81.3 | 51.6 | 5.8 | 0.810 | 0.642 | 2.90 | 2.0 | 6.28 | 8.86 | -8.86 | -1.231 | -1.195 | 4.92 | 0.110 | 0.544 | 0.537 | E |
| H026 | 0.555 | 148.8 | 73.1 | 36.5 | 38.9 | 0.716 | 0.116 | 2.79 | 1.0 | 4.85 | 2.01 | -2.01 | -0.938 | -0.933 | 4.41 | 0.074 | 0.345 | 0.341 | I |
| H027 | 0.473 | 164.2 | 73.0 | 53.3 | 44.0 | 0.879 | 0.105 | 4.72 | 2.0 | 5.21 | 4.88 | -4.88 | -0.967 | -0.923 | 5.26 | 0.089 | 0.414 | 0.411 | I |
| H028 | 0.365 | 170.4 | 43.6 | 51.5 | 45.9 | 0.669 | 0.106 | 3.90 | 3.0 | 3.98 | 5.17 | -5.17 | -0.807 | -0.844 | 3.65 | 0.060 | 0.275 | 0.277 | I |
| H029 | 0.355 | 118.1 | 53.6 | 35.6 | 39.0 | 0.417 | 0.122 | 2.10 | 5.0 | 3.83 | 8.83 | -8.83 | -0.793 | -0.756 | 2.19 | 0.059 | 0.267 | 0.260 | I |
| H030 | 0.744 | 129.8 | 79.5 | 43.3 | 7.9 | 0.582 | 0.414 | 2.53 | 5.0 | 5.30 | 16.46 | -16.46 | -1.146 | -1.037 | 3.31 | 0.088 | 0.424 | 0.425 | E |
| H031 | 0.463 | 172.6 | 45.4 | 49.4 | 8.5 | 0.729 | 0.257 | 4.11 | 1.0 | 6.20 | 3.30 | -3.30 | -1.336 | -1.099 | 4.62 | 0.101 | 0.482 | 0.482 | I |
| H032 | 0.479 | 176.0 | 63.4 | 36.4 | 26.6 | 0.781 | 0.132 | 3.17 | 2.0 | 5.28 | 5.06 | -5.06 | -1.079 | -1.021 | 4.69 | 0.091 | 0.432 | 0.427 | I |
| H033 | 0.370 | 189.0 | 95.7 | 40.3 | 6.9 | 1.234 | 0.389 | 5.73 | 0.5 | 7.88 | 3.34 | -3.34 | -1.538 | -1.398 | 7.32 | 0.167 | 0.798 | 0.796 | E |
| H034 | 0.311 | 172.4 | 84.1 | 37.5 | 20.9 | 0.938 | 0.143 | 3.92 | 5.0 | 6.09 | 20.30 | -20.30 | -1.210 | -1.072 | 5.77 | 0.104 | 0.498 | 0.494 | I |
| H035 | 0.468 | 136.0 | 35.8 | 49.0 | 41.3 | 0.542 | 0.110 | 2.62 | 1.0 | 3.56 | 1.35 | -1.35 | -0.802 | -0.801 | 2.79 | 0.057 | 0.251 | 0.252 | I |
| H036 | 0.598 | 148.3 | 91.9 | 13.5 | 12.7 | 0.754 | 0.196 | 2.24 | 5.0 | 4.94 | 11.58 | -11.58 | -1.031 | -0.985 | 4.27 | 0.083 | 0.387 | 0.385 | E |
| H037 | 0.417 | 127.2 | 66.9 | 56.1 | 14.5 | 0.558 | 0.174 | 2.55 | 0.5 | 4.72 | 1.31 | -1.31 | -1.107 | -1.009 | 3.31 | 0.070 | 0.344 | 0.345 | I |
| H038 | 0.454 | 164.5 | 83.4 | 5.2 | 25.3 | 0.686 | 0.145 | 2.30 | 5.0 | 4.69 | 7.79 | -7.79 | -0.866 | -0.896 | 3.67 | 0.074 | 0.341 | 0.336 | I |
| H039 | 0.270 | 166.3 | 81.6 | 5.8 | 43.8 | 0.691 | 0.117 | 2.40 | 2.0 | 3.75 | 1.56 | -1.56 | -0.781 | -0.782 | 3.72 | 0.066 | 0.286 | 0.285 | I |
| H040 | 0.607 | 154.6 | 30.2 | 54.3 | 39.6 | 0.608 | 0.106 | 2.92 | 1.0 | 3.40 | 1.73 | -1.73 | -0.801 | -0.843 | 3.21 | 0.054 | 0.243 | 0.242 | I |
| H041 | 0.554 | 154.1 | 79.9 | 50.3 | 37.2 | 0.797 | 0.116 | 3.66 | 3.0 | 5.23 | 8.82 | -8.82 | -1.147 | -0.977 | 4.71 | 0.085 | 0.402 | 0.404 | I |
| H042 | 0.258 | 107.5 | 46.2 | 14.6 | 28.1 | 0.281 | 0.139 | 1.44 | 1.0 | 3.09 | 0.99 | -0.99 | -0.717 | -0.826 | 1.49 | 0.048 | 0.204 | 0.200 | I |
| H043 | 0.274 | 166.6 | 75.9 | 14.6 | 41.7 | 0.737 | 0.110 | 2.57 | 5.0 | 4.27 | 7.68 | -7.68 | -0.773 | -0.849 | 4.09 | 0.075 | 0.336 | 0.336 | I |
| H044 | 0.278 | 165.8 | 51.8 | 43.6 | 22.8 | 0.599 | 0.129 | 3.41 | 2.0 | 5.30 | 4.15 | -4.15 | -1.103 | -1.030 | 3.59 | 0.084 | 0.406 | 0.396 | I |
| H045 | 0.733 | 149.8 | 98.2 | 17.0 | 24.7 | 0.873 | 0.142 | 2.52 | 1.0 | 5.05 | 1.90 | -1.90 | -1.028 | -0.939 | 5.03 | 0.087 | 0.388 | 0.391 | E |
| H046 | 0.489 | 117.0 | 36.4 | 34.0 | 18.3 | 0.334 | 0.164 | 1.97 | 1.0 | 2.93 | 1.31 | -1.31 | -0.831 | -0.852 | 1.69 | 0.045 | 0.201 | 0.199 | I |
| H047 | 0.621 | 132.3 | 81.6 | 55.5 | 42.1 | 0.745 | 0.114 | 2.92 | 5.0 | 4.94 | 13.93 | -13.93 | -1.048 | -0.995 | 4.63 | 0.081 | 0.385 | 0.390 | I |
| H048 | 0.662 | 167.4 | 38.8 | 56.8 | 18.2 | 0.685 | 0.151 | 3.99 | 3.0 | 4.94 | 8.05 | -8.05 | -1.040 | -1.021 | 3.87 | 0.077 | 0.359 | 0.358 | I |
| H049 | 0.737 | 134.9 | 31.3 | 59.7 | 9.5 | 0.523 | 0.311 | 2.51 | 3.0 | 3.56 | 6.50 | -6.50 | -0.951 | -0.931 | 2.76 | 0.052 | 0.248 | 0.251 | I |
| H050 | 0.292 | 115.8 | 27.0 | 22.8 | 36.6 | 0.338 | 0.125 | 1.75 | 5.0 | 2.67 | 4.92 | -4.92 | -0.752 | -0.726 | 1.50 | 0.047 | 0.189 | 0.188 | I |
| H051 | 0.708 | 163.9 | 70.2 | 54.3 | 27.2 | 0.773 | 0.126 | 3.96 | 2.0 | 5.60 | 7.32 | -7.32 | -1.162 | -1.010 | 4.80 | 0.085 | 0.410 | 0.408 | I |
| H052 | 0.467 | 159.3 | 66.1 | 41.7 | 19.1 | 0.726 | 0.140 | 4.08 | 3.0 | 5.44 | 7.55 | -7.55 | -1.131 | -0.967 | 4.19 | 0.089 | 0.429 | 0.431 | I |
| H053 | 0.749 | 129.0 | 61.5 | 52.0 | 10.9 | 0.558 | 0.217 | 2.71 | 5.0 | 4.52 | 11.29 | -11.29 | -1.051 | -0.950 | 3.40 | 0.068 | 0.325 | 0.328 | I |
| H054 | 0.656 | 104.7 | 73.9 | 27.9 | 46.4 | 0.514 | 0.120 | 2.19 | 1.0 | 3.90 | 1.02 | -1.02 | -0.817 | -0.735 | 2.91 | 0.059 | 0.272 | 0.274 | I |
| H055 | 0.442 | 124.5 | 61.2 | 56.0 | 23.0 | 0.553 | 0.148 | 2.72 | 3.0 | 4.51 | 8.52 | -8.52 | -1.063 | -0.948 | 3.56 | 0.065 | 0.309 | 0.305 | I |
| H056 | 0.390 | 100.9 | 74.7 | 45.4 | 35.2 | 0.556 | 0.126 | 2.35 | 2.0 | 3.74 | 4.26 | -4.26 | -0.930 | -0.891 | 3.24 | 0.054 | 0.255 | 0.261 | I |
| H057 | 0.481 | 100.8 | 85.0 | 43.0 | 9.6 | 0.532 | 0.404 | 2.29 | 5.0 | 4.16 | 13.96 | -13.96 | -1.043 | -0.952 | 3.26 | 0.065 | 0.302 | 0.303 | I |
| H058 | 0.255 | 155.2 | 99.1 | 18.5 | 46.3 | 0.920 | 0.108 | 2.70 | 1.0 | 4.49 | 1.38 | -1.38 | -0.844 | -0.798 | 5.17 | 0.080 | 0.366 | 0.366 | E |
| H059 | 0.623 | 146.2 | 64.8 | 53.5 | 32.8 | 0.668 | 0.123 | 2.88 | 1.0 | 4.84 | 2.63 | -2.63 | -1.055 | -0.927 | 3.88 | 0.077 | 0.355 | 0.351 | I |
| H060 | 0.476 | 185.9 | 95.0 | 30.4 | 7.8 | 1.083 | 0.303 | 5.01 | 1.0 | 7.49 | 5.09 | -5.09 | -1.433 | -1.305 | 6.46 | 0.131 | 0.633 | 0.628 | E |
| H061 | 0.346 | 147.2 | 66.6 | 56.1 | 37.5 | 0.810 | 0.111 | 3.12 | 1.0 | 4.38 | 2.26 | -2.26 | -1.048 | -0.946 | 4.58 | 0.077 | 0.354 | 0.347 | I |
| H062 | 0.475 | 123.2 | 78.0 | 49.2 | 43.0 | 0.618 | 0.113 | 2.56 | 0.5 | 4.88 | 1.12 | -1.12 | -0.954 | -0.923 | 3.29 | 0.078 | 0.368 | 0.369 | I |
| H063 | 0.433 | 159.8 | 53.0 | 35.9 | 23.8 | 0.667 | 0.141 | 2.86 | 5.0 | 4.51 | 12.49 | -12.49 | -1.025 | -0.940 | 3.99 | 0.073 | 0.348 | 0.351 | I |
| H064 | 0.531 | 114.8 | 89.8 | 5.5 | 41.9 | 0.518 | 0.121 | 1.51 | 5.0 | 3.00 | 2.72 | -2.72 | -0.690 | -0.711 | 2.49 | 0.050 | 0.207 | 0.209 | I |
| H065 | 0.417 | 163.6 | 68.4 | 13.2 | 46.1 | 0.715 | 0.106 | 2.54 | 1.0 | 4.08 | 1.26 | -1.26 | -0.715 | -0.734 | 3.86 | 0.071 | 0.329 | 0.326 | I |
| H066 | 0.299 | 176.9 | 31.3 | 7.3 | 10.4 | 0.531 | 0.286 | 2.39 | 0.5 | 5.09 | 1.22 | -1.22 | -1.092 | -0.989 | 3.36 | 0.078 | 0.361 | 0.366 | I |
| H067 | 0.623 | 159.5 | 47.5 | 47.0 | 30.5 | 0.684 | 0.117 | 3.00 | 0.5 | 4.21 | 1.07 | -1.07 | -1.020 | -0.963 | 3.79 | 0.066 | 0.308 | 0.306 | I |
| H068 | 0.287 | 169.6 | 28.1 | 25.5 | 38.4 | 0.558 | 0.108 | 2.62 | 3.0 | 3.60 | 4.54 | -4.54 | -0.862 | -0.780 | 2.93 | 0.064 | 0.284 | 0.285 | I |
| H069 | 0.486 | 127.1 | 45.8 | 10.1 | 22.8 | 0.523 | 0.150 | 1.75 | 5.0 | 3.47 | 8.15 | -8.15 | -0.827 | -0.807 | 2.82 | 0.056 | 0.246 | 0.247 | I |
| H070 | 0.711 | 103.2 | 76.8 | 58.5 | 6.6 | 0.584 | 0.712 | 2.43 | 3.0 | 4.29 | 8.42 | -8.42 | -1.056 | -1.010 | 3.50 | 0.070 | 0.334 | 0.334 | E |
| H071 | 0.472 | 104.0 | 76.3 | 38.0 | 10.7 | 0.513 | 0.295 | 2.00 | 0.5 | 4.40 | 0.89 | -0.89 | -1.037 | -0.940 | 3.14 | 0.068 | 0.316 | 0.316 | I |
| H072 | 0.451 | 140.5 | 29.3 | 10.7 | 30.8 | 0.403 | 0.127 | 1.74 | 0.5 | 3.22 | 0.66 | -0.66 | -0.729 | -0.761 | 2.09 | 0.049 | 0.212 | 0.210 | I |
| H073 | 0.466 | 128.7 | 87.1 | 15.6 | 19.2 | 0.564 | 0.167 | 2.06 | 5.0 | 3.99 | 8.60 | -8.60 | -0.932 | -0.809 | 3.02 | 0.063 | 0.290 | 0.294 | I |
| H074 | 0.466 | 108.8 | 44.3 | 57.8 | 49.2 | 0.406 | 0.110 | 2.32 | 0.5 | 3.12 | 0.60 | -0.60 | -0.731 | -0.798 | 2.17 | 0.047 | 0.208 | 0.205 | I |
| H075 | 0.402 | 135.2 | 91.6 | 37.4 | 31.7 | 0.726 | 0.126 | 2.52 | 3.0 | 4.87 | 7.27 | -7.27 | -1.053 | -0.976 | 4.65 | 0.081 | 0.388 | 0.380 | E |
| H076 | 0.707 | 181.9 | 94.3 | 49.7 | 43.4 | 1.180 | 0.101 | 6.98 | 2.0 | 6.05 | 7.72 | -7.72 | -1.118 | -1.034 | 6.57 | 0.115 | 0.553 | 0.553 | E |
| H077 | 0.496 | 115.2 | 49.5 | 59.5 | 30.3 | 0.483 | 0.133 | 2.50 | 3.0 | 4.16 | 5.20 | -5.20 | -0.866 | -0.861 | 2.68 | 0.065 | 0.287 | 0.286 | I |
| H078 | 0.701 | 132.0 | 98.6 | 30.6 | 31.4 | 0.759 | 0.125 | 2.67 | 2.0 | 4.69 | 5.29 | -5.29 | -0.977 | -0.955 | 4.30 | 0.077 | 0.352 | 0.354 | E |
| H079 | 0.570 | 165.6 | 87.1 | 50.2 | 29.4 | 0.895 | 0.119 | 3.79 | 1.0 | 6.08 | 4.32 | -4.32 | -1.166 | -1.062 | 5.43 | 0.103 | 0.494 | 0.490 | I |
| H080 | 0.718 | 116.3 | 69.3 | 35.3 | 5.9 | 0.503 | 0.771 | 2.06 | 1.0 | 4.32 | 2.19 | -2.19 | -1.037 | -0.948 | 2.85 | 0.069 | 0.323 | 0.328 | E |
| H081 | 0.409 | 153.9 | 95.2 | 49.6 | 12.1 | 0.876 | 0.189 | 3.91 | 0.5 | 6.78 | 2.20 | -2.20 | -1.347 | -1.220 | 5.20 | 0.112 | 0.536 | 0.535 | E |
| H082 | 0.745 | 187.7 | 50.7 | 24.5 | 25.1 | 0.768 | 0.139 | 3.05 | 5.0 | 4.99 | 14.06 | -14.06 | -1.060 | -0.970 | 4.52 | 0.077 | 0.365 | 0.368 | E |
| H083 | 0.470 | 181.4 | 38.2 | 40.4 | 25.1 | 0.705 | 0.132 | 4.03 | 3.0 | 4.82 | 7.22 | -7.22 | -1.033 | -1.020 | 3.88 | 0.078 | 0.368 | 0.366 | E |
| H084 | 0.536 | 131.9 | 90.8 | 52.4 | 27.4 | 0.724 | 0.127 | 2.94 | 0.5 | 5.24 | 1.40 | -1.40 | -1.138 | -0.992 | 4.60 | 0.085 | 0.425 | 0.419 | E |
| H085 | 0.287 | 119.5 | 85.7 | 40.3 | 36.3 | 0.567 | 0.124 | 2.69 | 2.0 | 4.79 | 5.22 | -5.22 | -0.872 | -0.903 | 3.31 | 0.072 | 0.336 | 0.338 | I |
| H086 | 0.633 | 145.1 | 66.7 | 32.8 | 7.1 | 0.620 | 0.488 | 2.48 | 3.0 | 5.19 | 8.86 | -8.86 | -1.126 | -1.020 | 3.87 | 0.083 | 0.403 | 0.398 | E |
| H087 | 0.368 | 110.4 | 26.1 | 26.5 | 35.8 | 0.309 | 0.121 | 1.83 | 0.5 | 2.70 | 0.61 | -0.61 | -0.705 | -0.770 | 1.46 | 0.042 | 0.175 | 0.176 | I |
| H088 | 0.669 | 140.8 | 53.6 | 57.7 | 12.2 | 0.694 | 0.191 | 2.74 | 5.0 | 5.08 | 16.05 | -16.05 | -1.166 | -1.019 | 4.03 | 0.074 | 0.352 | 0.352 | I |
| H089 | 0.280 | 169.3 | 44.6 | 17.0 | 19.9 | 0.703 | 0.147 | 2.56 | 1.0 | 5.09 | 2.32 | -2.32 | -0.944 | -0.945 | 4.06 | 0.089 | 0.414 | 0.419 | I |
| H090 | 0.389 | 153.4 | 57.1 | 13.2 | 8.5 | 0.680 | 0.378 | 2.04 | 3.0 | 5.05 | 7.58 | -7.58 | -1.113 | -0.960 | 3.73 | 0.078 | 0.365 | 0.369 | I |
| H091 | 0.632 | 117.7 | 52.6 | 11.4 | 43.1 | 0.379 | 0.116 | 1.69 | 2.0 | 2.96 | 1.87 | -1.87 | -0.654 | -0.716 | 1.92 | 0.048 | 0.196 | 0.196 | I |
| H092 | 0.736 | 163.5 | 77.6 | 55.7 | 23.5 | 0.845 | 0.137 | 4.40 | 1.0 | 5.57 | 3.05 | -3.05 | -1.178 | -1.063 | 5.02 | 0.096 | 0.458 | 0.456 | I |
| H093 | 0.504 | 157.7 | 20.6 | 29.9 | 30.4 | 0.503 | 0.126 | 2.34 | 5.0 | 3.65 | 8.22 | -8.22 | -0.805 | -0.814 | 2.47 | 0.056 | 0.252 | 0.257 | I |
| H094 | 0.251 | 186.6 | 97.9 | 10.3 | 49.3 | 1.061 | 0.108 | 2.95 | 0.5 | 3.98 | 0.44 | -0.44 | -0.757 | -0.836 | 5.57 | 0.083 | 0.358 | 0.354 | E |
| H095 | 0.446 | 126.8 | 46.8 | 34.6 | 27.8 | 0.492 | 0.141 | 2.15 | 2.0 | 3.63 | 3.48 | -3.48 | -0.862 | -0.880 | 2.68 | 0.056 | 0.261 | 0.260 | I |
| H096 | 0.351 | 179.7 | 56.0 | 45.2 | 32.9 | 0.832 | 0.113 | 4.19 | 1.0 | 5.18 | 2.50 | -2.50 | -1.072 | -1.004 | 4.60 | 0.086 | 0.402 | 0.403 | I |
| H097 | 0.499 | 154.7 | 43.6 | 9.7 | 11.5 | 0.453 | 0.224 | 2.14 | 1.0 | 4.33 | 2.44 | -2.44 | -0.951 | -0.943 | 2.81 | 0.065 | 0.309 | 0.312 | I |
| H098 | 0.255 | 134.8 | 78.5 | 30.4 | 40.3 | 0.621 | 0.121 | 2.63 | 0.5 | 4.07 | 0.85 | -0.85 | -0.947 | -0.871 | 3.59 | 0.065 | 0.305 | 0.301 | I |
| H099 | 0.294 | 125.2 | 70.1 | 19.2 | 26.8 | 0.571 | 0.143 | 1.98 | 1.0 | 3.84 | 1.61 | -1.61 | -0.864 | -0.831 | 3.06 | 0.059 | 0.276 | 0.273 | I |
| H100 | 0.614 | 114.3 | 91.0 | 38.0 | 30.6 | 0.658 | 0.130 | 2.46 | 2.0 | 4.56 | 5.54 | -5.54 | -1.025 | -0.940 | 3.82 | 0.076 | 0.350 | 0.350 | E |
| H101 | 0.655 | 139.0 | 56.4 | 44.6 | 34.5 | 0.543 | 0.125 | 2.59 | 0.5 | 3.87 | 0.86 | -0.86 | -0.941 | -0.908 | 3.11 | 0.056 | 0.255 | 0.256 | I |
| H102 | 0.509 | 121.0 | 83.7 | 23.2 | 21.1 | 0.545 | 0.157 | 2.28 | 1.0 | 4.57 | 1.81 | -1.81 | -0.898 | -0.909 | 3.15 | 0.070 | 0.313 | 0.313 | I |
| H103 | 0.665 | 148.4 | 33.4 | 51.0 | 29.0 | 0.577 | 0.127 | 2.66 | 5.0 | 4.25 | 10.02 | -10.02 | -0.860 | -0.897 | 3.30 | 0.070 | 0.324 | 0.325 | I |
| H104 | 0.594 | 168.1 | 37.7 | 49.9 | 25.3 | 0.726 | 0.130 | 3.01 | 1.0 | 4.63 | 2.35 | -2.35 | -1.031 | -0.916 | 4.14 | 0.076 | 0.358 | 0.364 | I |
| H105 | 0.585 | 138.9 | 42.9 | 16.4 | 15.2 | 0.524 | 0.185 | 1.94 | 5.0 | 3.64 | 8.13 | -8.13 | -0.897 | -0.900 | 3.19 | 0.055 | 0.251 | 0.249 | I |
| H106 | 0.596 | 126.1 | 45.7 | 45.8 | 11.5 | 0.440 | 0.204 | 2.45 | 0.5 | 4.22 | 0.99 | -0.99 | -0.970 | -0.912 | 2.38 | 0.059 | 0.272 | 0.270 | I |
| H107 | 0.402 | 131.7 | 72.0 | 16.2 | 26.4 | 0.468 | 0.142 | 2.04 | 1.0 | 4.04 | 1.90 | -1.90 | -0.819 | -0.865 | 2.62 | 0.060 | 0.272 | 0.269 | I |
| H108 | 0.358 | 134.9 | 60.5 | 19.7 | 14.9 | 0.498 | 0.183 | 2.20 | 5.0 | 4.45 | 12.61 | -12.61 | -0.941 | -0.923 | 2.61 | 0.070 | 0.317 | 0.324 | I |
| H109 | 0.645 | 110.6 | 48.3 | 53.9 | 49.1 | 0.512 | 0.106 | 2.29 | 5.0 | 3.36 | 8.15 | -8.15 | -0.721 | -0.845 | 2.64 | 0.053 | 0.227 | 0.223 | I |
| H110 | 0.547 | 142.7 | 23.5 | 47.5 | 30.3 | 0.405 | 0.128 | 2.45 | 5.0 | 3.08 | 6.89 | -6.89 | -0.840 | -0.802 | 2.18 | 0.049 | 0.213 | 0.213 | I |
| H111 | 0.421 | 106.7 | 32.9 | 17.6 | 11.7 | 0.286 | 0.249 | 1.68 | 1.0 | 2.74 | 1.21 | -1.21 | -0.773 | -0.801 | 1.41 | 0.042 | 0.186 | 0.185 | I |
| H112 | 0.557 | 129.2 | 31.4 | 14.9 | 38.0 | 0.392 | 0.118 | 1.73 | 2.0 | 2.79 | 1.98 | -1.98 | -0.733 | -0.743 | 1.84 | 0.042 | 0.188 | 0.191 | I |
| H113 | 0.713 | 174.5 | 55.1 | 33.6 | 11.2 | 0.818 | 0.195 | 3.01 | 2.0 | 6.09 | 5.20 | -5.20 | -1.154 | -1.078 | 5.06 | 0.105 | 0.506 | 0.507 | I |
| H114 | 0.400 | 114.1 | 68.3 | 6.8 | 29.5 | 0.359 | 0.143 | 1.59 | 5.0 | 2.69 | 4.46 | -4.46 | -0.671 | -0.778 | 1.65 | 0.042 | 0.184 | 0.183 | I |
| H115 | 0.492 | 163.6 | 62.5 | 40.3 | 36.8 | 0.725 | 0.114 | 2.97 | 0.5 | 4.63 | 1.15 | -1.15 | -1.014 | -0.882 | 4.02 | 0.081 | 0.386 | 0.376 | I |
| H116 | 0.722 | 185.4 | 80.7 | 14.3 | 5.8 | 0.876 | 0.652 | 2.85 | 5.0 | 6.48 | 16.45 | -16.45 | -1.300 | -1.121 | 5.26 | 0.128 | 0.610 | 0.606 | E |
| H117 | 0.435 | 177.2 | 57.9 | 8.4 | 17.7 | 0.726 | 0.163 | 2.44 | 0.5 | 5.32 | 1.08 | -1.08 | -1.054 | -0.947 | 4.22 | 0.085 | 0.408 | 0.407 | I |
| H118 | 0.718 | 131.6 | 82.5 | 15.8 | 19.4 | 0.660 | 0.157 | 1.91 | 5.0 | 4.53 | 9.09 | -9.09 | -0.896 | -0.908 | 4.02 | 0.068 | 0.316 | 0.318 | I |
| H119 | 0.589 | 138.8 | 74.5 | 13.3 | 14.8 | 0.578 | 0.195 | 1.99 | 2.0 | 4.79 | 3.48 | -3.48 | -1.034 | -0.921 | 3.63 | 0.084 | 0.379 | 0.373 | I |
| H120 | 0.300 | 168.8 | 22.0 | 36.2 | 21.7 | 0.542 | 0.129 | 2.96 | 0.5 | 4.08 | 0.92 | -0.92 | -0.936 | -0.916 | 2.86 | 0.062 | 0.291 | 0.289 | I |
| H121 | 0.645 | 166.2 | 35.2 | 28.5 | 12.6 | 0.576 | 0.198 | 2.82 | 1.0 | 4.97 | 2.62 | -2.62 | -1.118 | -1.007 | 3.59 | 0.078 | 0.369 | 0.375 | I |
| H122 | 0.562 | 138.5 | 35.7 | 35.8 | 26.6 | 0.396 | 0.137 | 2.26 | 2.0 | 3.83 | 3.61 | -3.61 | -0.896 | -0.866 | 2.11 | 0.055 | 0.241 | 0.244 | I |
| H123 | 0.429 | 133.8 | 24.4 | 6.2 | 38.2 | 0.203 | 0.123 | 1.11 | 1.0 | 3.22 | 1.39 | -1.39 | -0.667 | -0.709 | 1.18 | 0.048 | 0.204 | 0.206 | I |
| H124 | 0.602 | 132.5 | 92.2 | 41.3 | 14.1 | 0.731 | 0.178 | 2.59 | 1.0 | 5.79 | 2.42 | -2.42 | -1.095 | -1.038 | 4.52 | 0.093 | 0.445 | 0.444 | E |
| H125 | 0.532 | 125.2 | 65.8 | 16.0 | 28.0 | 0.552 | 0.137 | 2.01 | 0.5 | 3.43 | 0.63 | -0.63 | -0.845 | -0.864 | 2.98 | 0.056 | 0.240 | 0.240 | I |
| H126 | 0.406 | 135.3 | 96.7 | 58.3 | 44.3 | 0.823 | 0.119 | 4.12 | 0.5 | 5.25 | 1.76 | -1.76 | -1.092 | -1.010 | 4.94 | 0.091 | 0.430 | 0.428 | E |
| H127 | 0.660 | 133.3 | 26.2 | 37.2 | 20.0 | 0.486 | 0.151 | 2.24 | 2.0 | 3.77 | 2.82 | -2.82 | -0.840 | -0.841 | 2.67 | 0.058 | 0.258 | 0.257 | I |
| H128 | 0.656 | 161.4 | 35.6 | 31.2 | 48.5 | 0.530 | 0.102 | 2.68 | 2.0 | 3.56 | 2.58 | -2.58 | -0.730 | -0.770 | 2.90 | 0.062 | 0.259 | 0.263 | I |
| H129 | 0.498 | 119.6 | 48.2 | 19.5 | 12.3 | 0.420 | 0.207 | 1.90 | 0.5 | 3.48 | 0.79 | -0.79 | -0.861 | -0.854 | 2.20 | 0.055 | 0.248 | 0.248 | I |
| H130 | 0.567 | 113.7 | 40.2 | 10.7 | 38.7 | 0.277 | 0.119 | 1.35 | 2.0 | 2.82 | 2.12 | -2.12 | -0.625 | -0.712 | 1.28 | 0.041 | 0.171 | 0.171 | I |
| H131 | 0.671 | 120.0 | 50.8 | 49.5 | 15.2 | 0.465 | 0.173 | 2.55 | 5.0 | 3.95 | 13.03 | -13.03 | -0.979 | -0.957 | 2.58 | 0.057 | 0.261 | 0.258 | I |
| H132 | 0.510 | 162.1 | 71.2 | 16.6 | 20.7 | 0.709 | 0.152 | 2.52 | 1.0 | 5.16 | 2.53 | -2.53 | -1.034 | -0.978 | 3.96 | 0.072 | 0.335 | 0.336 | I |
| H133 | 0.734 | 141.0 | 95.6 | 44.6 | 44.3 | 0.868 | 0.113 | 2.90 | 0.5 | 5.02 | 1.11 | -1.11 | -1.040 | -0.934 | 5.00 | 0.090 | 0.417 | 0.417 | E |
| H134 | 0.330 | 179.5 | 60.8 | 22.5 | 10.0 | 0.853 | 0.273 | 2.93 | 2.0 | 6.51 | 7.04 | -7.04 | -1.224 | -1.048 | 5.06 | 0.118 | 0.569 | 0.568 | I |
| H135 | 0.455 | 163.3 | 50.4 | 52.2 | 8.6 | 0.678 | 0.304 | 3.47 | 2.0 | 5.80 | 5.78 | -5.78 | -1.173 | -1.117 | 4.37 | 0.090 | 0.429 | 0.430 | I |
| H136 | 0.600 | 124.2 | 89.5 | 7.4 | 9.8 | 0.609 | 0.370 | 1.78 | 0.5 | 4.01 | 0.53 | -0.53 | -0.894 | -0.864 | 3.34 | 0.062 | 0.283 | 0.286 | I |
| H137 | 0.632 | 187.2 | 91.3 | 26.9 | 5.8 | 1.187 | 0.542 | 5.00 | 5.0 | 7.55 | 25.17 | -25.17 | -1.438 | -1.268 | 6.87 | 0.167 | 0.810 | 0.797 | E |
| H138 | 0.477 | 182.6 | 37.5 | 16.1 | 5.5 | 0.625 | 0.716 | 2.76 | 5.0 | 6.05 | 15.79 | -15.79 | -1.156 | -1.114 | 3.70 | 0.108 | 0.520 | 0.528 | E |
| H139 | 0.406 | 114.1 | 61.1 | 37.8 | 15.5 | 0.441 | 0.185 | 2.19 | 5.0 | 4.30 | 9.44 | -9.44 | -0.951 | -0.899 | 2.46 | 0.063 | 0.292 | 0.290 | I |
| H140 | 0.585 | 152.3 | 51.8 | 30.5 | 26.9 | 0.660 | 0.130 | 2.64 | 3.0 | 4.67 | 6.29 | -6.29 | -0.987 | -0.891 | 4.02 | 0.078 | 0.363 | 0.361 | I |
| H141 | 0.338 | 148.1 | 38.6 | 58.2 | 37.5 | 0.615 | 0.106 | 2.50 | 2.0 | 4.14 | 3.65 | -3.65 | -0.900 | -0.847 | 3.43 | 0.065 | 0.297 | 0.293 | I |
| H142 | 0.474 | 150.3 | 73.2 | 11.5 | 25.6 | 0.621 | 0.134 | 2.26 | 3.0 | 4.11 | 5.55 | -5.55 | -0.924 | -0.891 | 3.54 | 0.065 | 0.298 | 0.304 | I |
| H143 | 0.720 | 101.0 | 60.8 | 8.8 | 18.4 | 0.385 | 0.168 | 1.44 | 1.0 | 2.98 | 1.02 | -1.02 | -0.741 | -0.808 | 1.84 | 0.047 | 0.200 | 0.197 | I |
| H144 | 0.531 | 160.3 | 79.6 | 20.9 | 15.1 | 0.810 | 0.167 | 2.74 | 2.0 | 5.36 | 5.78 | -5.78 | -1.178 | -1.017 | 4.80 | 0.091 | 0.425 | 0.423 | I |
| H145 | 0.577 | 137.1 | 43.3 | 55.0 | 48.7 | 0.479 | 0.112 | 2.64 | 5.0 | 3.81 | 7.74 | -7.74 | -0.747 | -0.803 | 2.41 | 0.066 | 0.280 | 0.279 | I |
| H146 | 0.748 | 150.5 | 51.2 | 7.7 | 30.5 | 0.543 | 0.140 | 2.02 | 2.0 | 3.63 | 2.77 | -2.77 | -0.793 | -0.776 | 2.83 | 0.056 | 0.250 | 0.251 | I |
| H147 | 0.546 | 189.0 | 95.2 | 50.3 | 16.7 | 1.317 | 0.151 | 6.33 | 3.0 | 7.71 | 18.59 | -18.59 | -1.445 | -1.322 | 7.55 | 0.150 | 0.732 | 0.745 | E |
| H148 | 0.349 | 101.4 | 44.9 | 8.6 | 44.8 | 0.269 | 0.117 | 1.16 | 0.5 | 2.65 | 0.28 | -0.28 | -0.616 | -0.708 | 1.02 | 0.040 | 0.164 | 0.163 | I |
| H149 | 0.316 | 110.9 | 46.7 | 32.5 | 43.3 | 0.339 | 0.109 | 2.19 | 2.0 | 3.67 | 2.94 | -2.94 | -0.730 | -0.762 | 1.77 | 0.057 | 0.243 | 0.243 | I |
| H150 | 0.641 | 105.4 | 84.5 | 18.8 | 27.6 | 0.582 | 0.142 | 1.96 | 2.0 | 3.78 | 2.13 | -2.13 | -0.884 | -0.845 | 3.54 | 0.061 | 0.279 | 0.278 | I |
| H151 | 0.656 | 170.2 | 61.5 | 42.7 | 34.6 | 0.753 | 0.115 | 4.07 | 1.0 | 5.06 | 2.39 | -2.39 | -0.954 | -0.926 | 4.48 | 0.081 | 0.379 | 0.379 | I |
| H152 | 0.705 | 111.1 | 45.4 | 7.1 | 46.6 | 0.243 | 0.112 | 1.47 | 1.0 | 2.85 | 0.65 | -0.65 | -0.630 | -0.659 | 1.36 | 0.044 | 0.183 | 0.184 | I |
| H153 | 0.439 | 146.2 | 41.4 | 40.0 | 48.7 | 0.562 | 0.107 | 2.55 | 5.0 | 3.20 | 7.86 | -7.86 | -0.742 | -0.816 | 2.78 | 0.050 | 0.224 | 0.222 | I |
| H154 | 0.350 | 135.2 | 22.1 | 49.8 | 41.6 | 0.399 | 0.117 | 2.56 | 2.0 | 3.03 | 1.91 | -1.91 | -0.693 | -0.738 | 1.84 | 0.050 | 0.211 | 0.208 | I |
| H155 | 0.349 | 161.6 | 54.6 | 44.3 | 35.0 | 0.713 | 0.118 | 3.61 | 2.0 | 4.82 | 4.54 | -4.54 | -0.981 | -0.913 | 4.06 | 0.083 | 0.389 | 0.382 | I |
| H156 | 0.520 | 161.1 | 55.7 | 45.2 | 21.4 | 0.673 | 0.145 | 2.89 | 5.0 | 5.48 | 17.22 | -17.22 | -1.121 | -1.047 | 3.98 | 0.089 | 0.411 | 0.403 | I |
| H157 | 0.314 | 118.1 | 91.2 | 13.9 | 21.0 | 0.672 | 0.155 | 1.95 | 5.0 | 4.09 | 6.83 | -6.83 | -0.922 | -0.876 | 3.76 | 0.064 | 0.294 | 0.299 | E |
| H158 | 0.534 | 170.8 | 86.4 | 35.4 | 40.6 | 0.915 | 0.112 | 3.66 | 5.0 | 5.38 | 12.77 | -12.77 | -1.018 | -0.947 | 5.11 | 0.090 | 0.421 | 0.421 | I |
| H159 | 0.745 | 163.2 | 46.4 | 23.7 | 49.0 | 0.618 | 0.109 | 2.57 | 1.0 | 3.55 | 1.40 | -1.40 | -0.770 | -0.799 | 3.55 | 0.058 | 0.268 | 0.273 | I |
| H160 | 0.640 | 128.5 | 78.6 | 44.1 | 5.0 | 0.669 | 0.832 | 2.48 | 2.0 | 5.10 | 5.03 | -5.03 | -1.168 | -1.070 | 4.16 | 0.095 | 0.457 | 0.447 | E |
| H161 | 0.412 | 136.5 | 52.7 | 29.1 | 44.7 | 0.526 | 0.114 | 2.39 | 1.0 | 3.48 | 1.55 | -1.55 | -0.750 | -0.788 | 2.78 | 0.056 | 0.246 | 0.251 | I |
| H162 | 0.489 | 121.4 | 41.5 | 52.0 | 9.2 | 0.434 | 0.366 | 2.56 | 1.0 | 4.16 | 1.86 | -1.86 | -0.947 | -0.923 | 2.31 | 0.066 | 0.307 | 0.299 | I |
| H163 | 0.696 | 139.6 | 64.8 | 26.1 | 41.0 | 0.595 | 0.116 | 2.44 | 0.5 | 3.93 | 0.71 | -0.71 | -0.805 | -0.858 | 3.23 | 0.058 | 0.273 | 0.275 | I |
| H164 | 0.408 | 140.4 | 51.8 | 40.2 | 36.5 | 0.570 | 0.120 | 2.58 | 1.0 | 4.08 | 1.50 | -1.50 | -0.841 | -0.854 | 3.22 | 0.061 | 0.276 | 0.278 | I |
| H165 | 0.676 | 157.5 | 96.7 | 16.3 | 30.9 | 0.805 | 0.126 | 2.70 | 2.0 | 5.16 | 3.62 | -3.62 | -0.952 | -0.881 | 4.68 | 0.083 | 0.391 | 0.389 | E |
| H166 | 0.444 | 168.8 | 49.0 | 28.0 | 45.4 | 0.706 | 0.109 | 2.73 | 0.5 | 4.18 | 0.99 | -0.99 | -0.840 | -0.796 | 3.85 | 0.073 | 0.318 | 0.315 | I |
| H167 | 0.439 | 138.9 | 54.2 | 26.2 | 12.0 | 0.521 | 0.206 | 2.38 | 1.0 | 4.81 | 2.34 | -2.34 | -0.977 | -0.899 | 3.11 | 0.072 | 0.328 | 0.330 | I |
| H168 | 0.707 | 181.4 | 66.9 | 28.1 | 5.4 | 0.766 | 0.665 | 3.01 | 2.0 | 6.49 | 11.55 | -11.55 | -1.351 | -1.145 | 4.90 | 0.117 | 0.567 | 0.576 | E |
| H169 | 0.407 | 114.5 | 42.9 | 23.3 | 22.0 | 0.335 | 0.167 | 1.97 | 3.0 | 3.21 | 5.29 | -5.29 | -0.814 | -0.794 | 2.06 | 0.049 | 0.216 | 0.214 | I |
| H170 | 0.462 | 153.4 | 77.4 | 6.8 | 47.4 | 0.705 | 0.110 | 2.08 | 1.0 | 3.94 | 0.81 | -0.81 | -0.660 | -0.652 | 3.68 | 0.065 | 0.272 | 0.277 | I |
| H171 | 0.720 | 146.6 | 41.7 | 47.4 | 11.0 | 0.537 | 0.202 | 2.82 | 5.0 | 4.99 | 13.60 | -13.60 | -1.076 | -0.965 | 3.16 | 0.080 | 0.376 | 0.380 | I |
| H172 | 0.391 | 169.3 | 40.1 | 9.4 | 14.7 | 0.622 | 0.185 | 2.15 | 0.5 | 4.96 | 1.04 | -1.04 | -0.973 | -0.983 | 3.72 | 0.074 | 0.346 | 0.350 | I |
| H173 | 0.682 | 174.8 | 45.6 | 42.0 | 20.1 | 0.723 | 0.142 | 3.36 | 2.0 | 5.59 | 5.77 | -5.77 | -1.123 | -1.024 | 4.32 | 0.092 | 0.429 | 0.428 | I |
| H174 | 0.713 | 113.6 | 77.7 | 40.4 | 6.8 | 0.528 | 0.609 | 2.38 | 0.5 | 4.31 | 1.21 | -1.21 | -1.096 | -0.944 | 3.38 | 0.069 | 0.320 | 0.324 | E |
| H175 | 0.251 | 175.3 | 43.0 | 36.5 | 12.0 | 0.775 | 0.172 | 3.62 | 2.0 | 5.97 | 5.36 | -5.36 | -1.113 | -1.060 | 4.73 | 0.099 | 0.477 | 0.476 | I |
| H176 | 0.735 | 187.4 | 99.4 | 43.6 | 6.7 | 1.311 | 0.401 | 6.50 | 2.0 | 8.19 | 16.64 | -16.64 | -1.588 | -1.381 | 7.59 | 0.175 | 0.842 | 0.846 | E |
| H177 | 0.568 | 120.4 | 62.3 | 51.3 | 18.6 | 0.499 | 0.167 | 2.44 | 5.0 | 4.46 | 14.66 | -14.66 | -0.942 | -0.927 | 3.31 | 0.072 | 0.346 | 0.340 | I |
| H178 | 0.577 | 157.4 | 76.0 | 35.3 | 39.8 | 0.679 | 0.112 | 3.16 | 3.0 | 4.85 | 5.85 | -5.85 | -0.962 | -0.925 | 4.05 | 0.078 | 0.357 | 0.358 | I |
| H179 | 0.597 | 115.3 | 35.6 | 54.8 | 5.3 | 0.396 | 0.855 | 2.59 | 0.5 | 3.59 | 0.84 | -0.84 | -0.875 | -0.921 | 2.12 | 0.056 | 0.261 | 0.260 | E |
| H180 | 0.541 | 166.8 | 67.2 | 28.2 | 28.4 | 0.815 | 0.126 | 2.86 | 0.5 | 4.98 | 1.13 | -1.13 | -0.989 | -0.898 | 4.94 | 0.075 | 0.354 | 0.356 | I |
| H181 | 0.290 | 107.4 | 27.0 | 18.7 | 46.5 | 0.232 | 0.116 | 1.50 | 1.0 | 2.33 | 1.04 | -1.04 | -0.687 | -0.708 | 1.07 | 0.040 | 0.153 | 0.150 | I |
| H182 | 0.706 | 173.6 | 75.9 | 45.4 | 49.0 | 0.951 | 0.101 | 4.02 | 1.0 | 5.15 | 2.27 | -2.27 | -0.931 | -0.891 | 5.46 | 0.095 | 0.439 | 0.437 | I |
| H183 | 0.562 | 129.1 | 22.2 | 35.9 | 45.2 | 0.411 | 0.115 | 2.29 | 2.0 | 3.21 | 1.58 | -1.58 | -0.646 | -0.743 | 1.87 | 0.051 | 0.207 | 0.205 | I |
| H184 | 0.729 | 149.0 | 63.6 | 26.8 | 34.6 | 0.666 | 0.125 | 2.62 | 0.5 | 4.69 | 0.94 | -0.94 | -0.910 | -0.892 | 3.89 | 0.078 | 0.355 | 0.354 | I |
| H185 | 0.512 | 177.1 | 21.0 | 22.7 | 11.3 | 0.572 | 0.198 | 2.63 | 1.0 | 4.85 | 2.47 | -2.47 | -1.066 | -0.985 | 3.45 | 0.081 | 0.387 | 0.384 | I |
| H186 | 0.375 | 144.8 | 33.0 | 12.3 | 45.1 | 0.357 | 0.118 | 2.23 | 3.0 | 2.89 | 4.05 | -4.05 | -0.734 | -0.688 | 1.79 | 0.047 | 0.200 | 0.198 | I |
| H187 | 0.331 | 120.3 | 27.3 | 8.6 | 11.9 | 0.248 | 0.254 | 1.34 | 0.5 | 3.05 | 0.46 | -0.46 | -0.760 | -0.778 | 1.34 | 0.044 | 0.198 | 0.204 | I |
| H188 | 0.321 | 106.8 | 52.5 | 50.7 | 7.2 | 0.378 | 0.573 | 2.32 | 1.0 | 4.19 | 2.08 | -2.08 | -1.015 | -0.950 | 2.15 | 0.069 | 0.308 | 0.306 | E |
| H189 | 0.614 | 155.5 | 94.8 | 19.8 | 30.5 | 0.829 | 0.132 | 2.74 | 3.0 | 4.71 | 6.15 | -6.15 | -0.977 | -0.886 | 4.73 | 0.084 | 0.402 | 0.407 | E |
| H190 | 0.394 | 160.4 | 76.2 | 58.0 | 40.2 | 0.873 | 0.106 | 4.21 | 5.0 | 5.31 | 11.57 | -11.57 | -1.019 | -1.003 | 5.21 | 0.091 | 0.429 | 0.423 | I |
| H191 | 0.272 | 178.1 | 53.9 | 40.8 | 12.5 | 0.759 | 0.182 | 3.26 | 2.0 | 6.26 | 5.98 | -5.98 | -1.156 | -1.136 | 4.57 | 0.099 | 0.468 | 0.467 | I |
| H192 | 0.687 | 149.9 | 56.5 | 29.1 | 34.9 | 0.677 | 0.121 | 2.42 | 0.5 | 3.83 | 0.98 | -0.98 | -0.915 | -0.847 | 4.11 | 0.063 | 0.288 | 0.288 | I |
| H193 | 0.621 | 165.1 | 50.9 | 14.9 | 28.4 | 0.551 | 0.134 | 2.52 | 1.0 | 4.22 | 1.67 | -1.67 | -0.879 | -0.883 | 3.06 | 0.065 | 0.303 | 0.301 | I |
| H194 | 0.604 | 186.7 | 97.7 | 52.8 | 36.7 | 1.270 | 0.110 | 5.97 | 3.0 | 6.33 | 17.25 | -17.25 | -1.225 | -1.087 | 6.93 | 0.121 | 0.590 | 0.596 | E |
| H195 | 0.398 | 117.2 | 30.8 | 8.3 | 32.1 | 0.307 | 0.131 | 1.41 | 5.0 | 3.03 | 6.06 | -6.06 | -0.725 | -0.787 | 1.72 | 0.046 | 0.192 | 0.189 | I |
| H196 | 0.724 | 148.9 | 77.6 | 31.9 | 11.1 | 0.665 | 0.224 | 2.74 | 1.0 | 5.47 | 2.82 | -2.82 | -1.112 | -1.066 | 3.97 | 0.096 | 0.450 | 0.450 | I |
| H197 | 0.428 | 134.4 | 61.5 | 57.7 | 13.1 | 0.604 | 0.180 | 2.87 | 0.5 | 4.95 | 1.41 | -1.41 | -1.058 | -1.002 | 3.63 | 0.072 | 0.337 | 0.341 | I |
| H198 | 0.587 | 157.0 | 23.5 | 24.3 | 16.2 | 0.445 | 0.163 | 2.49 | 3.0 | 3.93 | 6.46 | -6.46 | -0.923 | -0.876 | 2.59 | 0.055 | 0.250 | 0.253 | I |
| H199 | 0.462 | 133.4 | 45.6 | 46.3 | 18.5 | 0.476 | 0.162 | 2.54 | 2.0 | 4.15 | 4.56 | -4.56 | -0.956 | -0.897 | 2.90 | 0.058 | 0.281 | 0.280 | I |
| H200 | 0.314 | 187.7 | 96.6 | 5.4 | 19.3 | 1.061 | 0.170 | 2.90 | 1.0 | 6.19 | 2.03 | -2.03 | -1.028 | -0.959 | 5.80 | 0.107 | 0.516 | 0.518 | E |
| H201 | 0.311 | 169.2 | 73.6 | 7.9 | 48.9 | 0.736 | 0.111 | 2.47 | 5.0 | 3.89 | 6.47 | -6.47 | -0.775 | -0.705 | 4.30 | 0.069 | 0.302 | 0.301 | I |
| H202 | 0.732 | 163.3 | 49.8 | 48.8 | 47.6 | 0.701 | 0.102 | 4.04 | 0.5 | 4.09 | 0.83 | -0.83 | -0.842 | -0.887 | 3.76 | 0.068 | 0.311 | 0.312 | I |
| H203 | 0.470 | 184.0 | 72.5 | 9.7 | 32.1 | 0.952 | 0.125 | 2.93 | 5.0 | 4.95 | 10.38 | -10.38 | -0.895 | -0.877 | 5.41 | 0.083 | 0.386 | 0.384 | E |
| H204 | 0.396 | 165.1 | 85.8 | 31.8 | 15.6 | 0.892 | 0.170 | 3.20 | 2.0 | 5.90 | 5.36 | -5.36 | -1.137 | -1.056 | 5.32 | 0.098 | 0.472 | 0.475 | I |
| H205 | 0.290 | 110.8 | 46.7 | 19.8 | 14.5 | 0.371 | 0.193 | 1.80 | 0.5 | 3.54 | 0.60 | -0.60 | -0.879 | -0.841 | 1.81 | 0.055 | 0.235 | 0.232 | I |
| H206 | 0.479 | 123.6 | 46.3 | 42.8 | 48.1 | 0.439 | 0.108 | 2.16 | 5.0 | 3.27 | 6.64 | -6.64 | -0.772 | -0.811 | 2.30 | 0.049 | 0.223 | 0.223 | I |
| H207 | 0.594 | 173.2 | 57.0 | 47.6 | 35.4 | 0.707 | 0.120 | 3.60 | 0.5 | 4.98 | 1.37 | -1.37 | -0.983 | -0.986 | 4.33 | 0.084 | 0.391 | 0.393 | I |
| H208 | 0.265 | 150.3 | 74.5 | 7.9 | 33.5 | 0.713 | 0.131 | 2.09 | 1.0 | 3.92 | 0.99 | -0.99 | -0.786 | -0.852 | 3.99 | 0.064 | 0.290 | 0.284 | I |
| H209 | 0.401 | 174.8 | 55.7 | 55.8 | 16.8 | 0.773 | 0.154 | 5.09 | 2.0 | 6.15 | 7.13 | -7.13 | -1.326 | -1.090 | 4.78 | 0.098 | 0.485 | 0.474 | I |
| H210 | 0.528 | 138.9 | 43.8 | 16.5 | 18.5 | 0.467 | 0.159 | 2.17 | 2.0 | 3.81 | 3.35 | -3.35 | -0.882 | -0.878 | 2.67 | 0.061 | 0.274 | 0.274 | I |
| H211 | 0.350 | 137.3 | 31.6 | 23.8 | 32.9 | 0.403 | 0.121 | 1.97 | 5.0 | 3.08 | 7.09 | -7.09 | -0.834 | -0.771 | 2.11 | 0.047 | 0.206 | 0.203 | I |
| H212 | 0.642 | 157.7 | 86.8 | 54.0 | 26.4 | 0.881 | 0.132 | 4.33 | 3.0 | 6.18 | 14.37 | -14.37 | -1.273 | -1.056 | 5.38 | 0.103 | 0.497 | 0.496 | I |
| H213 | 0.307 | 163.0 | 64.5 | 44.9 | 39.8 | 0.829 | 0.125 | 4.40 | 2.0 | 4.86 | 5.46 | -5.46 | -0.998 | -0.930 | 4.92 | 0.086 | 0.398 | 0.395 | I |
| H214 | 0.264 | 183.0 | 92.4 | 13.7 | 7.2 | 1.040 | 0.493 | 3.72 | 1.0 | 6.68 | 3.21 | -3.21 | -1.300 | -1.141 | 5.88 | 0.118 | 0.565 | 0.575 | E |
| H215 | 0.466 | 137.9 | 46.3 | 39.8 | 47.9 | 0.544 | 0.103 | 2.65 | 2.0 | 3.38 | 2.60 | -2.60 | -0.743 | -0.783 | 3.13 | 0.055 | 0.247 | 0.249 | I |
| H216 | 0.495 | 174.0 | 50.1 | 15.9 | 36.8 | 0.604 | 0.118 | 2.71 | 0.5 | 4.24 | 0.81 | -0.81 | -0.853 | -0.862 | 3.31 | 0.070 | 0.311 | 0.308 | I |
| H217 | 0.456 | 182.0 | 76.3 | 13.4 | 14.8 | 0.846 | 0.178 | 2.97 | 0.5 | 6.09 | 1.24 | -1.24 | -1.161 | -1.007 | 4.97 | 0.101 | 0.496 | 0.489 | E |
| H218 | 0.523 | 101.7 | 83.1 | 12.2 | 48.1 | 0.451 | 0.117 | 1.60 | 0.5 | 3.44 | 0.33 | -0.33 | -0.681 | -0.756 | 2.01 | 0.060 | 0.239 | 0.244 | I |
| H219 | 0.527 | 141.3 | 93.1 | 42.8 | 43.8 | 0.810 | 0.109 | 2.88 | 5.0 | 4.95 | 12.14 | -12.14 | -0.991 | -0.943 | 4.48 | 0.086 | 0.400 | 0.380 | E |
| H220 | 0.524 | 156.7 | 37.8 | 33.8 | 7.8 | 0.598 | 0.409 | 2.62 | 0.5 | 4.83 | 1.05 | -1.05 | -1.099 | -0.992 | 3.90 | 0.080 | 0.376 | 0.369 | E |
| H221 | 0.528 | 119.3 | 40.8 | 24.3 | 23.1 | 0.435 | 0.149 | 2.01 | 2.0 | 3.14 | 3.64 | -3.64 | -0.805 | -0.809 | 2.14 | 0.046 | 0.203 | 0.202 | I |
| H222 | 0.633 | 133.5 | 24.4 | 18.5 | 19.2 | 0.299 | 0.166 | 1.75 | 1.0 | 3.48 | 1.67 | -1.67 | -0.876 | -0.821 | 1.50 | 0.060 | 0.246 | 0.243 | I |
| H223 | 0.339 | 172.0 | 60.8 | 6.8 | 47.7 | 0.659 | 0.115 | 2.35 | 1.0 | 3.40 | 1.20 | -1.20 | -0.734 | -0.758 | 3.36 | 0.060 | 0.259 | 0.261 | I |
| H224 | 0.717 | 106.5 | 21.6 | 37.5 | 49.4 | 0.299 | 0.110 | 2.06 | 5.0 | 2.42 | 5.05 | -5.05 | -0.705 | -0.687 | 1.57 | 0.041 | 0.161 | 0.158 | I |
| H225 | 0.571 | 101.8 | 72.7 | 23.4 | 42.1 | 0.451 | 0.129 | 1.91 | 1.0 | 3.68 | 1.16 | -1.16 | -0.767 | -0.756 | 2.47 | 0.058 | 0.258 | 0.258 | I |
| H226 | 0.542 | 147.6 | 75.4 | 19.9 | 34.1 | 0.692 | 0.120 | 2.42 | 3.0 | 4.41 | 6.58 | -6.58 | -0.959 | -0.851 | 3.98 | 0.069 | 0.329 | 0.333 | I |
| H227 | 0.322 | 150.8 | 23.0 | 35.7 | 5.3 | 0.503 | 0.745 | 2.55 | 1.0 | 4.48 | 2.23 | -2.23 | -1.069 | -0.981 | 2.92 | 0.075 | 0.353 | 0.364 | E |
| H228 | 0.258 | 126.4 | 62.6 | 33.6 | 6.8 | 0.577 | 0.629 | 2.56 | 2.0 | 4.57 | 4.31 | -4.31 | -1.107 | -0.978 | 3.12 | 0.079 | 0.372 | 0.371 | E |
| H229 | 0.737 | 173.6 | 33.2 | 37.3 | 45.8 | 0.693 | 0.114 | 2.77 | 1.0 | 3.77 | 1.72 | -1.72 | -0.804 | -0.753 | 3.90 | 0.056 | 0.257 | 0.268 | I |
| H230 | 0.593 | 162.5 | 30.2 | 40.5 | 22.9 | 0.555 | 0.139 | 2.78 | 5.0 | 4.13 | 12.36 | -12.36 | -0.974 | -0.932 | 3.23 | 0.062 | 0.284 | 0.287 | I |
| H231 | 0.362 | 160.7 | 63.4 | 45.6 | 38.5 | 0.699 | 0.111 | 4.12 | 2.0 | 4.78 | 4.83 | -4.83 | -0.991 | -0.902 | 4.07 | 0.081 | 0.378 | 0.380 | I |
| H232 | 0.443 | 162.6 | 83.2 | 39.9 | 21.0 | 0.886 | 0.142 | 3.73 | 5.0 | 6.01 | 14.50 | -14.50 | -1.190 | -1.059 | 5.30 | 0.094 | 0.454 | 0.453 | I |
| H233 | 0.587 | 184.3 | 27.9 | 31.8 | 34.5 | 0.700 | 0.115 | 4.32 | 1.0 | 4.09 | 1.92 | -1.92 | -0.857 | -0.909 | 4.01 | 0.067 | 0.304 | 0.303 | E |
| H234 | 0.331 | 161.2 | 56.1 | 33.7 | 33.8 | 0.678 | 0.120 | 2.80 | 0.5 | 4.15 | 0.91 | -0.91 | -1.001 | -0.904 | 4.01 | 0.066 | 0.311 | 0.315 | I |
| H235 | 0.320 | 140.0 | 48.1 | 29.6 | 45.1 | 0.530 | 0.107 | 2.32 | 5.0 | 3.35 | 7.79 | -7.79 | -0.821 | -0.806 | 2.74 | 0.052 | 0.230 | 0.230 | I |
| H236 | 0.676 | 145.7 | 30.6 | 27.9 | 28.6 | 0.462 | 0.127 | 2.33 | 1.0 | 3.11 | 1.54 | -1.54 | -0.835 | -0.814 | 2.57 | 0.048 | 0.208 | 0.208 | I |
| H237 | 0.302 | 159.9 | 28.4 | 24.1 | 49.3 | 0.550 | 0.109 | 2.46 | 2.0 | 2.71 | 2.16 | -2.16 | -0.690 | -0.737 | 2.88 | 0.049 | 0.203 | 0.202 | I |
| H238 | 0.458 | 134.3 | 71.7 | 23.0 | 49.7 | 0.564 | 0.111 | 2.24 | 0.5 | 3.90 | 0.73 | -0.73 | -0.810 | -0.807 | 2.74 | 0.064 | 0.278 | 0.278 | I |
| H239 | 0.477 | 159.8 | 40.6 | 51.4 | 20.8 | 0.667 | 0.137 | 2.98 | 5.0 | 5.05 | 13.22 | -13.22 | -0.992 | -0.966 | 3.85 | 0.070 | 0.335 | 0.339 | I |
| H240 | 0.319 | 126.1 | 35.9 | 54.5 | 6.5 | 0.454 | 0.632 | 2.68 | 0.5 | 3.83 | 0.92 | -0.92 | -0.934 | -0.905 | 2.54 | 0.066 | 0.304 | 0.306 | E |
| H241 | 0.747 | 189.9 | 42.3 | 6.9 | 17.7 | 0.725 | 0.164 | 2.73 | 5.0 | 5.41 | 13.62 | -13.62 | -0.998 | -0.888 | 4.30 | 0.084 | 0.397 | 0.399 | E |
| H242 | 0.372 | 160.4 | 49.1 | 44.4 | 19.8 | 0.700 | 0.155 | 2.98 | 2.0 | 5.19 | 6.06 | -6.06 | -1.044 | -1.012 | 4.29 | 0.090 | 0.424 | 0.424 | I |
| H243 | 0.728 | 179.2 | 49.2 | 15.4 | 29.3 | 0.674 | 0.128 | 2.50 | 2.0 | 4.84 | 4.56 | -4.56 | -0.932 | -0.920 | 3.67 | 0.082 | 0.378 | 0.378 | I |
| H244 | 0.377 | 177.9 | 52.4 | 9.6 | 33.0 | 0.647 | 0.125 | 2.63 | 2.0 | 4.64 | 3.35 | -3.35 | -0.821 | -0.893 | 3.63 | 0.081 | 0.360 | 0.366 | I |
| H245 | 0.451 | 178.4 | 63.2 | 16.8 | 35.3 | 0.761 | 0.119 | 3.02 | 0.5 | 4.40 | 0.91 | -0.91 | -0.886 | -0.902 | 4.19 | 0.078 | 0.357 | 0.360 | I |
| H246 | 0.629 | 111.7 | 62.5 | 44.1 | 20.0 | 0.509 | 0.157 | 2.12 | 1.0 | 4.27 | 2.09 | -2.09 | -0.926 | -0.934 | 2.51 | 0.070 | 0.306 | 0.306 | I |
| H247 | 0.560 | 151.8 | 51.8 | 31.3 | 28.3 | 0.591 | 0.121 | 2.56 | 1.0 | 4.38 | 1.85 | -1.85 | -0.936 | -0.925 | 3.38 | 0.067 | 0.302 | 0.297 | I |
| H248 | 0.293 | 106.9 | 64.9 | 18.2 | 14.8 | 0.501 | 0.196 | 1.92 | 3.0 | 3.83 | 4.82 | -4.82 | -0.854 | -0.834 | 3.10 | 0.069 | 0.314 | 0.317 | I |
| H249 | 0.285 | 141.3 | 40.7 | 41.2 | 16.6 | 0.499 | 0.163 | 2.31 | 3.0 | 4.37 | 5.30 | -5.30 | -0.950 | -0.899 | 2.86 | 0.068 | 0.305 | 0.305 | I |
| H250 | 0.410 | 108.8 | 24.5 | 46.4 | 17.5 | 0.270 | 0.184 | 2.18 | 3.0 | 2.91 | 3.44 | -3.44 | -0.788 | -0.780 | 1.19 | 0.042 | 0.181 | 0.182 | I |
| H251 | 0.703 | 150.7 | 36.4 | 58.4 | 29.2 | 0.594 | 0.124 | 2.85 | 1.0 | 4.25 | 1.81 | -1.81 | -0.907 | -0.910 | 3.49 | 0.068 | 0.318 | 0.324 | I |
| H252 | 0.504 | 162.2 | 60.1 | 55.4 | 19.3 | 0.729 | 0.149 | 3.26 | 0.5 | 5.54 | 1.53 | -1.53 | -1.184 | -1.017 | 4.39 | 0.086 | 0.422 | 0.418 | I |
| H253 | 0.342 | 173.0 | 88.4 | 40.2 | 31.0 | 1.055 | 0.119 | 3.60 | 2.0 | 6.12 | 8.22 | -8.22 | -1.201 | -1.060 | 6.04 | 0.109 | 0.513 | 0.513 | I |
| H254 | 0.279 | 180.7 | 73.0 | 35.8 | 18.9 | 0.876 | 0.153 | 4.86 | 3.0 | 6.24 | 10.55 | -10.55 | -1.210 | -1.048 | 5.17 | 0.106 | 0.510 | 0.510 | E |
| H255 | 0.416 | 179.7 | 35.9 | 15.0 | 32.5 | 0.648 | 0.128 | 2.65 | 5.0 | 4.13 | 8.84 | -8.84 | -0.926 | -0.873 | 3.54 | 0.065 | 0.294 | 0.293 | I |
| H256 | 0.280 | 102.8 | 46.7 | 45.4 | 29.3 | 0.361 | 0.136 | 2.09 | 2.0 | 3.89 | 2.73 | -2.73 | -0.911 | -0.843 | 1.86 | 0.061 | 0.267 | 0.267 | I |
| H257 | 0.289 | 138.5 | 93.6 | 49.9 | 13.0 | 0.758 | 0.185 | 3.17 | 1.0 | 6.26 | 4.06 | -4.06 | -1.278 | -1.134 | 4.66 | 0.093 | 0.449 | 0.450 | E |
| H258 | 0.715 | 102.8 | 53.3 | 33.5 | 38.8 | 0.403 | 0.127 | 2.04 | 0.5 | 3.55 | 0.54 | -0.54 | -0.773 | -0.763 | 1.81 | 0.057 | 0.244 | 0.242 | I |
| H259 | 0.477 | 143.9 | 30.9 | 26.7 | 20.8 | 0.488 | 0.147 | 2.15 | 5.0 | 3.23 | 8.58 | -8.58 | -0.916 | -0.865 | 2.61 | 0.049 | 0.223 | 0.223 | I |
| H260 | 0.445 | 145.9 | 69.2 | 38.1 | 6.8 | 0.734 | 0.515 | 2.80 | 3.0 | 5.62 | 9.12 | -9.12 | -1.182 | -1.062 | 4.23 | 0.101 | 0.477 | 0.469 | E |
| H261 | 0.489 | 113.9 | 25.4 | 35.8 | 49.4 | 0.248 | 0.111 | 1.86 | 1.0 | 2.22 | 0.77 | -0.77 | -0.616 | -0.698 | 1.13 | 0.034 | 0.140 | 0.135 | I |
| H262 | 0.255 | 146.9 | 53.4 | 53.5 | 10.2 | 0.603 | 0.216 | 3.26 | 2.0 | 5.28 | 6.84 | -6.84 | -1.091 | -0.975 | 3.30 | 0.083 | 0.394 | 0.384 | I |
| H263 | 0.739 | 115.5 | 38.9 | 20.9 | 31.1 | 0.318 | 0.134 | 1.92 | 5.0 | 3.12 | 8.48 | -8.48 | -0.791 | -0.808 | 1.67 | 0.046 | 0.187 | 0.190 | I |
| H264 | 0.453 | 129.4 | 40.7 | 27.1 | 22.5 | 0.439 | 0.153 | 2.31 | 2.0 | 3.42 | 3.82 | -3.82 | -0.853 | -0.881 | 2.41 | 0.053 | 0.234 | 0.239 | I |
| H265 | 0.442 | 128.3 | 27.9 | 28.6 | 11.7 | 0.304 | 0.226 | 2.18 | 0.5 | 3.43 | 0.83 | -0.83 | -0.884 | -0.847 | 1.70 | 0.053 | 0.242 | 0.240 | I |
| H266 | 0.261 | 157.6 | 43.1 | 8.2 | 28.8 | 0.539 | 0.136 | 2.22 | 5.0 | 3.69 | 6.54 | -6.54 | -0.781 | -0.842 | 2.69 | 0.058 | 0.257 | 0.260 | I |
| H267 | 0.322 | 182.9 | 92.9 | 40.4 | 7.4 | 1.095 | 0.351 | 5.70 | 3.0 | 7.68 | 21.40 | -21.40 | -1.470 | -1.354 | 6.47 | 0.164 | 0.785 | 0.778 | E |
| H268 | 0.511 | 133.4 | 98.8 | 43.7 | 15.2 | 0.779 | 0.172 | 2.99 | 2.0 | 5.89 | 7.15 | -7.15 | -1.205 | -1.118 | 4.86 | 0.106 | 0.510 | 0.509 | E |
| H269 | 0.498 | 163.0 | 38.3 | 15.8 | 47.0 | 0.524 | 0.103 | 2.48 | 3.0 | 3.13 | 3.99 | -3.99 | -0.746 | -0.786 | 2.72 | 0.054 | 0.232 | 0.231 | I |
| H270 | 0.375 | 154.4 | 61.8 | 39.1 | 41.0 | 0.665 | 0.113 | 2.92 | 0.5 | 4.32 | 1.01 | -1.01 | -0.895 | -0.891 | 3.95 | 0.070 | 0.320 | 0.319 | I |
| H271 | 0.628 | 111.1 | 23.6 | 39.0 | 49.6 | 0.331 | 0.117 | 2.10 | 2.0 | 2.53 | 1.33 | -1.33 | -0.613 | -0.701 | 1.61 | 0.040 | 0.169 | 0.167 | I |
| H272 | 0.436 | 125.6 | 97.7 | 59.7 | 24.2 | 0.766 | 0.142 | 2.94 | 0.5 | 6.00 | 1.63 | -1.63 | -1.221 | -1.050 | 4.70 | 0.091 | 0.440 | 0.443 | E |
| H273 | 0.271 | 176.9 | 68.9 | 21.9 | 17.7 | 0.806 | 0.154 | 3.78 | 3.0 | 5.79 | 8.40 | -8.40 | -1.087 | -1.025 | 5.00 | 0.099 | 0.475 | 0.473 | I |
| H274 | 0.436 | 177.2 | 28.9 | 5.9 | 28.7 | 0.546 | 0.140 | 2.15 | 5.0 | 4.29 | 9.74 | -9.74 | -0.833 | -0.851 | 3.32 | 0.065 | 0.294 | 0.293 | I |
| H275 | 0.658 | 119.9 | 71.4 | 36.7 | 45.2 | 0.611 | 0.112 | 2.37 | 1.0 | 4.19 | 1.87 | -1.87 | -0.795 | -0.830 | 3.65 | 0.065 | 0.292 | 0.290 | I |
| H276 | 0.607 | 159.4 | 64.3 | 27.2 | 14.8 | 0.781 | 0.172 | 2.79 | 0.5 | 5.08 | 1.10 | -1.10 | -1.180 | -0.992 | 4.84 | 0.088 | 0.419 | 0.417 | I |
| H277 | 0.289 | 163.3 | 66.8 | 57.7 | 46.7 | 0.818 | 0.110 | 3.63 | 2.0 | 4.92 | 5.17 | -5.17 | -1.010 | -0.960 | 4.76 | 0.079 | 0.363 | 0.366 | I |
| H278 | 0.547 | 154.9 | 57.0 | 37.0 | 36.7 | 0.642 | 0.123 | 2.76 | 5.0 | 4.56 | 9.02 | -9.02 | -0.864 | -0.874 | 3.59 | 0.072 | 0.334 | 0.343 | I |
| H279 | 0.449 | 127.0 | 33.8 | 41.7 | 31.9 | 0.425 | 0.127 | 2.08 | 3.0 | 3.22 | 4.41 | -4.41 | -0.765 | -0.796 | 2.23 | 0.053 | 0.228 | 0.227 | I |
| H280 | 0.440 | 164.8 | 33.3 | 21.6 | 44.6 | 0.599 | 0.111 | 2.53 | 5.0 | 2.88 | 6.36 | -6.36 | -0.780 | -0.751 | 3.38 | 0.046 | 0.209 | 0.210 | I |

**Table S11 Two level stratification and partition counts**

Cited in Section 4.1. The data source defines the first stratification level, the hot pressing temperature interval the second level within the experimental branch, and the interface orientation the second level within the first principles branch.

| **Source** | **Stratification variable** | **Stratum** | **Total** | **Training** | **Validation** | **Interpolation test** | **Extrapolation test** |
| --- | --- | --- | --- | --- | --- | --- | --- |
| First principles | Interface orientation | (100)/(100) | 272 | 218 | 34 | 20 | 0 |
| First principles | Interface orientation | (110)/(110) | 266 | 213 | 33 | 20 | 0 |
| First principles | Interface orientation | (111)/(111) | 262 | 209 | 33 | 20 | 0 |
| Published experiments | Reported temperature interval | [100,130) °C | 38 | 30 | 8 | 0 | 0 |
| Published experiments | Reported temperature interval | [130,160) °C | 44 | 35 | 9 | 0 | 0 |
| Published experiments | Reported temperature interval | [160,190] °C | 38 | 31 | 7 | 0 | 0 |
| In-house experiments | Hot pressing temperature interval | [100,130) °C | 88 | 34 | 18 | 19 | 17 |
| In-house experiments | Hot pressing temperature interval | [130,160) °C | 96 | 36 | 19 | 21 | 20 |
| In-house experiments | Hot pressing temperature interval | [160,190] °C | 96 | 34 | 19 | 20 | 23 |
| Total | — | — | 1200 | 840 | 180 | 120 | 60 |

**Table S12 First principles computational protocol for the 800 calculated records**

Cited in Section 4.1. The corresponding input file templates are reproduced in Supplementary Section S8.

| **Item** | **Setting** |
| --- | --- |
| Code and version | VASP 6.4.1, plane wave total energy scheme [37] |
| Exchange correlation functional | PBE generalized gradient approximation [39] |
| Core and valence treatment | Projector augmented wave potentials [38], In_d and Pb_d of the recommended PAW PBE set, In 4d5s5p and Pb 5d6s6p as valence |
| Interface supercell | Coincidence site lattice slabs of 128 to 192 atoms, lattice mismatch below 3%, 15 Å vacuum, dipole correction |
| Defect density levels | Vacancy concentrations of 0.5%, 1.0% and 2.0% on the In sublattice, three random realizations per level |
| Plane wave cutoff | 500 eV |
| k point mesh | Gamma centred 12×12×12 for bulk cells, 12×12×1 for interface slabs |
| Convergence criteria | 1×10⁻⁶ eV electronic, 0.01 eV·Å⁻¹ ionic |
| Spin-orbit coupling | Non collinear second variational treatment, LSORBIT switched on |
| Applied magnetic field | External Zeeman term $\mu_B B$ in the non collinear Hamiltonian, $B$ from ±0.5, ±1, ±2, ±3 and ±5 T |
| SOC strength | Fitted from the Pb-6p band splitting after Wannier downfolding on an 8×8×8 k mesh |
| Orbital projection | Site and orbital resolved projection, LORBIT set to 11 |
| Bader charge | Grid based Bader decomposition of the all electron density on a 480×480×480 grid |
| Barrier height | Planar averaged electrostatic potential across the interface |
| Intrinsic transverse conductivity | Berry curvature integration on a 200×200×200 Wannier interpolated mesh under the Zeeman term |
| Ordinary transverse conductivity | Semiclassical Boltzmann transport on the same bands, constant relaxation time fitted to the measured $\sigma_{xx}$ |
| Magnetoelectric susceptibility | Finite electric field of 0.01 V·Å⁻¹ normal to the interface, magnetization integrated within 1 nm of the boundary |

**Table S13 Delivery of the four input blocks to every model**

Cited in Section 4.1. Every model receives the same information, so the comparison of Section 4.2 isolates the effect of architecture rather than of input availability.

| **Model** | **Atomic and interfacial blocks** | **Mesoscale and processing blocks** | **Field conditioning** |
| --- | --- | --- | --- |
| CGCNN | Crystal graph of the representative supercell | Seven scalars concatenated with the pooled graph embedding before the readout MLP | Appended to the same scalar vector |
| ALIGNN | Crystal graph with its line graph | Seven scalars concatenated with the pooled embedding | Appended to the same scalar vector |
| CrabNet | Composition embedding | Seven scalars concatenated with the pooled composition embedding | Appended to the same scalar vector |
| MEGNet | Crystal graph with a global state vector | Seven scalars placed in the global state vector | Placed in the global state vector |
| Equiv-GNN | Equivariant crystal graph | Seven scalars concatenated with the pooled equivariant embedding | Appended to the same scalar vector |
| MLP | Three atomic and three interfacial scalars | Three mesoscale and four processing scalars | One additional input dimension |
| XGBoost | Three atomic and three interfacial scalars | Three mesoscale and four processing scalars | One additional feature |
| MSCA-EMNet | Token level hierarchical encoder | Mesoscale tokens in the encoder, processing parameters in the FiLM channel | FiLM conditioning vector |

**Supplementary Section S14 Reference implementation of the three core modules**

Cited in Sections 3.3, 3.4 and 3.5. The listings correspond to Equations (9) to (15), (16) and (17), and (19) to (25) of the main text.

**S14.1 Core forward propagation of BS-CrossAttn**

```
def bs_cross_attn(H_list, H_macro, W_BU, W_TD, gamma_BU, gamma_TD, d_h, n_head):
    """H_list: [H_atom, H_interface, H_meso], each of shape (B_s, 3, d_h)
       H_macro: (B_s, 5, d_h), key and value of the top-down branch only"""
    d_k = d_h // n_head
    updated, attn_bu, attn_td = [], [], []
    for k in range(len(H_list)):
        H_k = H_list[k]                                 # (B_s, n_k, d_h)
        H_new = H_k.clone()
        if k > 0:                                       # bottom-up, key and value from k-1
            Q = split_heads(H_k @ W_BU['Q'][k], n_head)  # (B_s, n_head, n_k, d_k)
            K = split_heads(H_list[k - 1] @ W_BU['K'][k], n_head)
            V = split_heads(H_list[k - 1] @ W_BU['V'][k], n_head)
            S = matmul(Q, K.transpose(-2, -1)) / sqrt(d_k)
            A = softmax(S, dim=-1)                      # softmax over the key token axis
            H_new = H_new + gamma_BU * merge_heads(matmul(A, V))
            attn_bu.append(A.mean(dim=1))               # (B_s, n_k, n_k-1)
        src = H_list[k + 1] if k < len(H_list) - 1 else H_macro
        Q = split_heads(H_k @ W_TD['Q'][k], n_head)     # top-down, key and value from k+1
        K = split_heads(src @ W_TD['K'][k], n_head)
        V = split_heads(src @ W_TD['V'][k], n_head)
        S = matmul(Q, K.transpose(-2, -1)) / sqrt(d_k)
        A = softmax(S, dim=-1)
        H_new = H_new + gamma_TD * merge_heads(matmul(A, V))
        attn_td.append(A.mean(dim=1))
        updated.append(H_new)
# composed maps of Equation (15), used only for interpretation
A_bar_bu = matmul(attn_bu[1], attn_bu[0])   # mesoscale <- atomic
A_bar_td = matmul(matmul(attn_td[0], attn_td[1]), attn_td[2])   # atomic <- macro
return updated, attn_bu, attn_td, A_bar_bu, A_bar_td
```

**S14.2 FiLM modulation layer with the absent process embedding**

```
class FiLMLayer(nn.Module):
    def __init__(self, proc_dim, field_dim, feat_dim, hidden=64):
        super().__init__()
        # learnable absent process embedding, used when no hot pressing history exists
        self.u_c = nn.Parameter(torch.zeros(proc_dim))
        cond_dim = proc_dim + field_dim
        self.gen_gamma = nn.Sequential(
            nn.Linear(cond_dim, hidden), nn.ReLU(), nn.Linear(hidden, feat_dim))
        self.gen_beta = nn.Sequential(
            nn.Linear(cond_dim, hidden), nn.ReLU(), nn.Linear(hidden, feat_dim))
        # initial identity mapping: gamma is about 1, beta is about 0
        nn.init.zeros_(self.gen_gamma[-1].weight)
        nn.init.ones_(self.gen_gamma[-1].bias)
        nn.init.zeros_(self.gen_beta[-1].weight)
        nn.init.zeros_(self.gen_beta[-1].bias)
    def forward(self, z, c, B, m_c):
        # m_c: (B_s, 1), equal to 1 when the record carries hot pressing parameters
        c_eff = m_c * c + (1.0 - m_c) * self.u_c
        xi = torch.cat([c_eff, B], dim=-1)
        return self.gen_gamma(xi) * z + self.gen_beta(xi)
```

**S14.3 Training loop with the two physical constraints**

```
def train_step(model, batch, optimizer, lambda_ons, lambda_me, s):
    X_a, X_i, X_m = batch['X_a'], batch['X_i'], batch['X_m']
    c, m_c, B, Y_true = batch['c'], batch['m_c'], batch['B'], batch['Y_true']
    # two forward passes differing only in the sign of the field channel
    Y_pos = model(X_a, X_i, X_m, c, m_c, B)
    Y_neg = model(X_a, X_i, X_m, c, m_c, -B)
    w, mask = model.component_weights, batch['label_mask']
    L_data = (mask * w * (Y_pos - Y_true) ** 2).sum() / mask.sum()
    # constraints on de-standardised values, normalised by the training std
    P_pos = model.destandardise(Y_pos)
    P_neg = model.destandardise(Y_neg)
    r_ons = P_pos[..., 1] / s[1] - P_neg[..., 2] / s[2]   # sigma_xy(+B) vs sigma_yx(-B)
    r_me = P_pos[..., 7] / s[7] - P_pos[..., 8] / s[8]    # alpha_xy^ME vs alpha_yx^EM
    L = L_data + lambda_ons * (r_ons ** 2).mean() + lambda_me * (r_me ** 2).mean()
    optimizer.zero_grad(); L.backward(); optimizer.step()
    return L.item()
```
